# Nrf2 induces malignant transformation of hepatic progenitor cells by inducing β-catenin expression

**DOI:** 10.1016/j.redox.2022.102453

**Published:** 2022-09-13

**Authors:** Athanassios Fragoulis, Julia Schenkel, Nicole Schröder, Elisa Fabiana Brandt, Mathias Weiand, Tabita Neu, Pierluigi Ramadori, Tim Caspers, Sebastian Kant, Thomas Pufe, Antje Mohs, Christian Trautwein, Thomas Longerich, Konrad Ludwig Streetz, Christoph Jan Wruck

**Affiliations:** a Department of Anatomy and Cell Biology, Uniklinik RWTH Aachen, Germany; b Department of Medicine III, Uniklinik RWTH Aachen, Germany; c Division of Chronic Inflammation and Cancer, German Cancer Research Center (DKFZ), Heidelberg, Germany; d Institute of Molecular and Cellular Anatomy, Uniklinik RWTH Aachen, Germany; e Institute of Pathology, University Hospital Heidelberg, Germany

## Abstract

The Nrf2 signaling pathway prevents cancer initiation, but genetic mutations that activate this pathway are found in various types of cancer. The molecular mechanisms underlying this Janus-headed character are still not understood. Here, we show that sustained Nrf2 activation induces proliferation and dedifferentiation of a Wnt-responsive perivenular hepatic progenitor cell population, transforming them into metastatic cancer cells. The neoplastic lesions display many histological features known from human hepatoblastoma. We describe an Nrf2-induced upregulation of β-catenin expression and its activation as the underlying mechanism for the observed malignant transformation. Thus, we have identified the Nrf2–β-catenin axis promoting proliferation of hepatic stem cells and triggering tumorigenesis. These findings support the concept that different functional levels of Nrf2 control both the protection against various toxins as well as liver regeneration by activating hepatic stem cells. Activation of the hepatic stem cell compartment confers the observation that unbridled Nrf2 activation may trigger tumorigenesis.

## 1 Introduction

It is well established that the transcription factor Nrf2, also called nuclear factor (erythroid-derived 2)-like 2 or NFE2L2, is the main regulator of the defense against oxidative stress. Nrf2 is constitutively and ubiquitously expressed. Its turnover is mainly controlled at the level of protein stability. Under basal conditions, the proteasome rapidly degrades Nrf2 via a Kelch-like ECH associated protein 1 (Keap1)–E3 ubiquitin-ligase complex interaction. However, reactive molecules such as reactive oxygen species (ROS) modify Keap1 to prevent degradation, resulting in Nrf2 accumulation. In addition, the phosphatidylinositol-4,5-bisphosphate 3-kinase (PI3K)–serine/threonine kinase (AKT) signaling pathway can also induce Nrf2 stabilization by inhibiting GSK3-β-TrCP-induced proteasomal degradation of Nrf2. After accumulation and nuclear translocation, Nrf2 binds to the antioxidant (or electrophilic) response element ARE/EpRE in the promoter region of its target genes—which code for antioxidative as well as detoxifying enzymes and proteins—to induce their expression [1]. Moreover, Nrf2 is activated by various cytokines and growth factors; and it regulates the expression of genes that influence tissue regeneration, such as myoblast determination protein 1 (MyoD), Notch, amphiregulin, vascular endothelial growth factor (VEGF) and Interleulin-6 (IL-6), indicating that Nrf2 activity reaches beyond oxidative stress defense [[2], [3], [4], [5]]. In fact, as we have shown in murine muscle and liver regeneration models, Nrf2 is required for stem cell proliferation in response to injury [6,7]. This dual role of Nrf2 in protecting tissue—by defending against oxidative stress on one hand and on the other by inducing tissue stem cells to induce regeneration—is reflected by its dual role in cancer, especially in lung, bladder and head and neck [8]. Although Nrf2 acts to prevent cancer by defending against oxidative stress, gain-of-function mutations in genes that encode the Nrf2 signaling pathway are found in many cancers, causing cell survival, metabolic reprogramming and tumor growth [[9], [10], [11], [12], [13], [14], [15], [16], [17]].

β-catenin is a structural protein in the cadherin-mediated cell-cell adhesive system. After activation, it acts as a transcription factor. In this function it plays an important role in cell differentiation and tissue repair, but also in the carcinogenesis [18]. In the liver, the Wnt/β-catenin signaling pathway has been shown to be the main regulator of liver zonation. β-catenin activity is highest around the central vein of the liver lobules. Here, β-catenin is involved particularly in glutamine synthesis, drug metabolism, and bile acid and heme synthesis [18]. Interestingly, the Nusse laboratory described a Wnt-responsive hepatocyte subpopulation surrounding the central vein that may play a role in homeostatic hepatocyte renewal and liver regeneration [19,20].

There is growing evidence that gain-of-function mutations in the gene that encodes for Nrf2 is often associated with mutations of the *CTNNB1* gene encoding β-catenin in liver cancer, suggesting an association between these two pathways in liver tumorigenesis [[21], [22], [23], [24], [25]]. Indeed, Nrf2 and β-catenin share several regulatory pathways [26]. Both β-catenin and Nrf2 are activated by Wnt3a, which prevents Axin1/GSK-3-dependent phosphorylation and subsequent β-TrCP-dependent degradation of both factors [27]. Moreover, E-cadherin recruits Nrf2 to the cell membrane by facilitating Nrf2/β-catenin interaction preventing NRF2 activation [28]. Besides, Nrf2 and β-catenin share the same regulatory pathways, Nrf2 and β-catenin upregulate common target genes such as amphiregulin, VEGF and Sox9 [3,4,[29], [30], [31]]. On the cellular level, it is noteworthy that both transcription factors can activate liver stem cells and are important for liver regeneration [7,20,32,33].

In this study, we investigated whether persistent Nrf2 activation triggers liver cancer. We used a transgenic mouse strain that allows permanent, liver-specific Nrf2 activation and investigated its liver phenotype over the course of up to 90 weeks. We were able to show that Nrf2 upregulates the expression of β-catenin directly by binding to a functional ARE sequence in its promoter. Nrf2 operated synergistically with β-catenin to induce a robust proliferation, dedifferentiation and malignant transformation in a specific Wnt-reactive hepatocyte population surrounding the central veins. We have thus discovered a previously unknown link between Nrf2 and β-catenin in hepatic progenitor cells which is also associated with tumorigenesis.

## 2 Results

### 2.1 Nrf2 activation induces hepatomegaly and metastatic cancer

Since gain-of-function mutations in genes that encode for the Nrf2 pathway are found in various cancers, we hypothesized that constitutive activation of Nrf2 by hepatic Keap1 deletion spontaneously induces liver carcinogenesis. To test this hypothesis, we used a transgenic mouse line that allows permanent, liver-specific Nrf2 activation (Keap1:Alb-Cre). Alb-Cre negative Keap1 littermates (WT) served as controls for all Keap1:Alb-Cre positive mice experiments and were housed together during the experiments. It should be noted that the floxing of the gene encoding Keap1 has resulted in a hypomorphic mutation, leading to overall higher baseline activation of Nrf2 than in WT mice [34]. Keap1:Alb-Cre mice that also lacked Nrf2 (Keap1:Alb-Cre:Nrf2^−/−^) served as controls to verify a Nrf2-dependency of observed effects. These mouse lines were observed for at least 90 weeks.

**Fig. S1:**
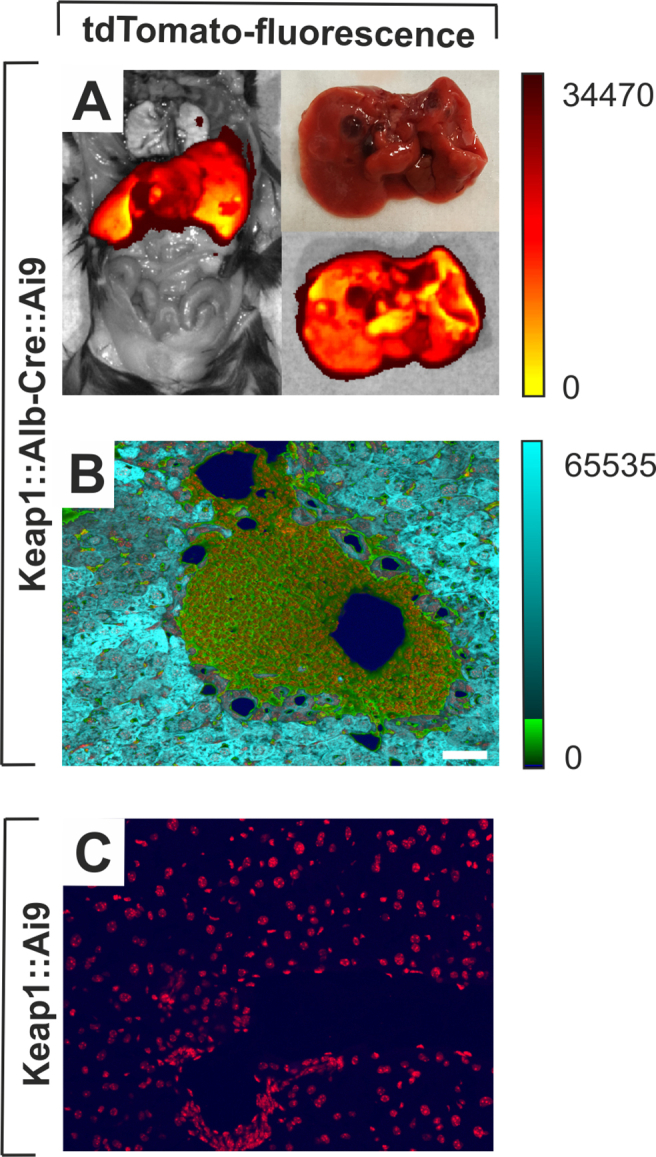
(*A*) Representative image of *post mortem* tdTomato fluorescence measurements from Keap1::Alb-Cre::Ai9 mice after opening the thoracic and abdominal cavities as well as after preparation of the liver. (n=3). Fluorescence intensities (relative fluorescence units = RFU) are shown using a false color scale. These analyses confirmed that Cre recombinase is expressed exclusively in mouse liver, as no tdTomato signal was detected in other tissues. (*B*) Representative image of fluorescence measurements of liver tissue sections derived from Keap1::Alb-Cre::Ai9 mice (n=3). Resulting confocal images were color-coded blue for no, green for low and cyan for high tdTomato fluorescence. Nuclei are shown in red. Please note that all hepatocytes show high intensity of tdTomato fluorescence and HLN-cells presented a low intensity of tdTomato fluorescence. This indicates at least some Alb-Cre activity within HLN-cells. (Scale bar = 50μm). (*C*) Fluorescence imaging of liver tissue sections derived from Keap1::Ai9 mice without Cre-expression (Cre-negative). Color-coding and imaging parameters were identical to parameters in B. Please note the lack of dtTomato fluorescence in this control image.

A Cre-recombinase-dependent fluorescence reporter gene (tdTomato, Exc/Em = 554nm/581nm) has been crossed into the Keap1:Alb-Cre mouse line (Keap1:Alb-Cre:Ai9) to test whether the albumin-promoter driven Cre-recombinase had been active in the liver. Imaging demonstrated that tdTomato expression in the liver was several orders of magnitude higher than background tissue autofluorescence (Fig. S1). For further examination, we extracted and separately measured the livers in the device. These images show the strong, liver-specific fluorescence signal caused by the Alb-Cre driven recombinase (Fig. S1A). Since the Alb-Cre allele was highly active in the liver, the question arose if the cells forming the HLN are derived from cells expressing Alb-Cre. Therefore, liver tissue sections derived from Keap1:Alb-Cre:Ai9 mice underwent fluorescence imaging for tdTomato and resulting images were color-coded as described in the materials and methods. As anticipated, all hepatocytes showed a high intensity of tdTomato fluorescence validating Alb-Cre expression of those hepatocytes (Fig. S1B). In contrast, HLN-cells presented only a low intensity of tdTomato fluorescence, which in contrast was completely absent in Cre-negative controls (Fig. S1C), indicating at least that Alb-Cre was active during HLN-cell development. One can only speculate why fluorescence intensity of HLN cells was lower. Since once the Cre activates tdTomato expression, the intensity of the fluorescence does not necessarily correlate with Cre-mediated DNA recombination. For this reason, we hypothesize that the observed lower fluorescence signal is due to a reduced nuclear-to-cytoplasmic ratio of HLN cells compared to hepatocytes.

**Fig. 1:**
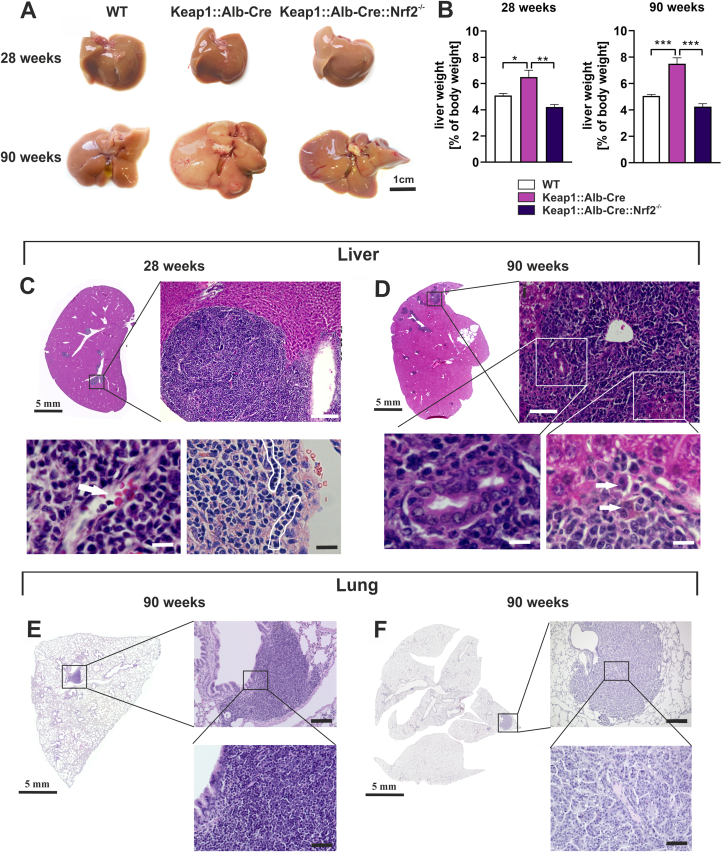
Continuous Nrf2 activation induces hepatomegaly and metastatic hepatoblastoma-like neoplasias. (*A*) Representative macroscopic pictures of livers from 28- and 90-week-old wild-type (WT), Keap1:Alb-Cre and Keap1:Alb-Cre:Nrf2^−/−^ mice are shown. (Scale bar = 1 cm). (*B*) Statistical analysis of the liver-to-body-weight ratios of 28- and 90-week-old wild-type (WT), Keap1:Alb-Cre and Keap1:Alb-Cre:Nrf2^−/−^ mice. Data represent mean ± SEM, one-way ANOVA with the Bonferroni post hoc test; biological replicates were WT mice: n = 21; Keap1:Alb-Cre mice: n = 27; Keap1:Alb-Cre:Nrf2^−/−^ mice: n = 7, *p < 0.05, **p < 0.005 & ***p < 0.001 as indicated. (*C*) Representative microscopic pictures of H&E-stained liver sections from 28-week-old Keap1:Alb-Cre mice. (top left) comprehensive view (Scale bar = 5 mm). (top right) Detail enlargement of an HLN lesion and the adjacent area between tumor and liver parenchyma. (Scale bar = 200 μm). (bottom left) Further enlargement of an HLN lesion; arrow points to a blood vessel containing erythrocytes. (Scale bar = 20 μm). (bottom right) Further enlargement of an HLN lesion; framed area encloses hepatoblastoma-typical, pearl necklace-like structures. (Scale bar = 50 μm). (*D*) Representative microscopic pictures of H&E-stained liver sections from 90-week-old Keap1:Alb-Cre mice. (top left) comprehensive view (Scale bar = 5 mm). (top right) Detail enlargement of an HLN lesion. (Scale bar = 100 μm). (bottom left) Detail enlargement of cholangioblastic re-differentiation among HLN. (Scale bar = 20 μm). (bottom right) Further enlargement of neoplasia-adjacent area showing hepatocytes of different sizes and cytosol/nucleus ratios. (Scale bar = 20 μm). (*E* and *F*) Representative microscopic pictures of H&E-stained lung sections from 90 week-old Keap1:Alb-Cre mice. (*E*) (left) Comprehensive view (Scale bar = 5 mm). (top right) Detail enlargement of lung metastasis with hepatoblastoma phenotype. (Scale bar = 500 μm). (bottom right) Further enlargement of the picture top right. (Scale bar = 50 μm). (*F*) (left) Comprehensive view. (Scale bar = 5 mm). (top right) Detail enlargement of lung metastasis with more differentiated phenotype. (Scale bar = 500 μm). (bottom right) Detail enlargement of picture top right. (Scale bar = 50 μm).

**Fig. S2:**
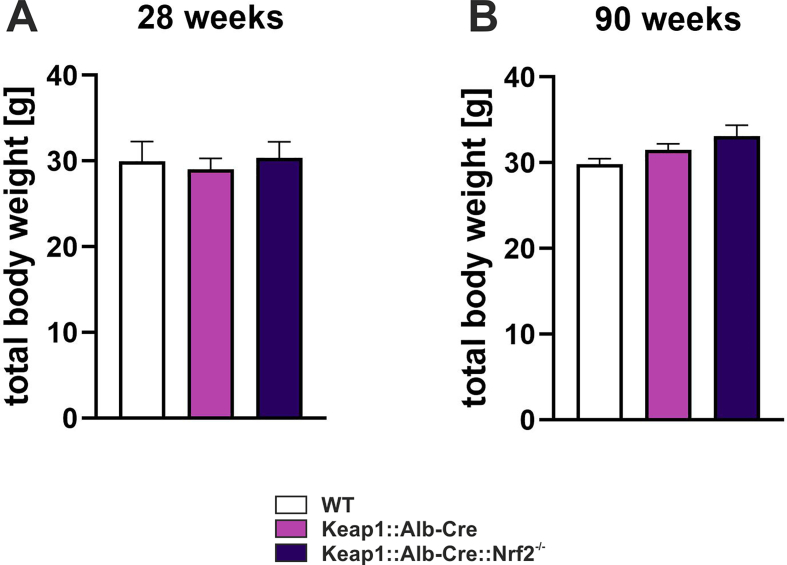
Statistical analysis of the total body-weight of 28 *(A)* - and 90 *(B)* -week-old wild-type (WT), Keap1::Alb-Cre and Keap1::Alb-Cre::Nrf2^-/-^ mice. Data represent mean ± SEM, one-way ANOVA with the Bonferroni post hoc test; biological replicates were WT mice: n = 21; Keap1::Alb-Cre mice: n = 27; Keap1::Alb-Cre:Nrf2^-/-^ mice: n = 7, * p < 0.05, ** p < 0.005 *** p < 0.001 as indicated.

The first apparent result was that 28-week-old Keap1:Alb-Cre mice showed a significant higher liver-to-body weight ratio (5.54% ± 0.88%) compared to WT (4.01% ± 0.88%) littermates and Keap1:Alb-Cre:Nrf2^−/−^ mice (4.25% ± 0.53%) (Fig. 1A (upper row of photographs) and B). At the age of 90 weeks, Keap1:Alb-Cre mice exhibited a remarkable hepatomegaly (liver/body weight 7.84% ± 3.0), which was not found in the control mouse lines (WT 5.32% ± 0.84%; Keap1:Alb-Cre:Nrf2^−/−^ mice 4.27% ± 1.19) (Fig. 1A (lower row of photographs) and c). No significant difference were observed between WT and Keap1:Alb-Cre:Nrf2^−/−^ mice. To ensure that the different body to liver ratios were not due to a difference in body weights, we have presented the body weights of the mice studied in Supplemental Fig. 2. This clearly demonstrates that Nrf2 activity, rather than the deletion of Keap1, was the driving force behind the observed hepatomegaly. Moreover, these data neatly confirm the findings of Wakabayashi et al. [35].

Histological examination of dissected livers from 28-week-old Keap1:Alb-Cre mice (n = 7) revealed that a neoplasia showing features of hepatoblastoma developed in almost all of them (6/7, 86%; Table 1) ; we therefore named it “hepatoblastoma-like neoplasia” (HLN). Interestingly, these neoplasms were found exclusively in close vicinity to central veins (Fig. 1C and D). HLNs could not be found in WT (n = 12) nor in Keap1:Alb-Cre:Nrf2^−/−^ mice (n = 7). HLN were vascularized (Fig. 1C arrow), and the non-cohesive neoplastic cells sometimes formed single-file linear cords typically observed in carcinomas with lack of E-cadherin expression (e.g. lobular carcinoma of the breast or poorly cohesive gastric cancer) (Fig. 1C white bordered).

**Table 1:** Prevalence of HLN development in 28-week-old mice (Number of mice with neoplasia/number of mice screened for neoplasia).

Genotype	HLN occurrence
wild type	0/7 (0%)
Keap1::Alb-Cre::Nrf2^−/−^	0/5 (0%)
Keap1::β-catenin::Alb-Cre	3/7 (43%)
Keap1::Alb-Cre	6/7 (86%)

To document the further course of permanent Nrf2 activation, we examined the livers of 90-week-old mice of all three genotypes. Eighty-eight per cent (22 out of 25) of the 90-week-old Keap1:Alb-Cre mice developed HLN. HLN lesions occurred more frequently, and were bigger in size, in the 90-week-old mice than in those 28 weeks old (Fig. 1D). The HLN were typically composed of small monomorphic cells with scant cytoplasm resembling blastemal liver cells (Fig. 1D). In some HLN cohesive aggregates and the formation of tubular structures consistent with a focal cholangioblastic differentiation was evident (Fig. 1D). Interestingly, some cells at the interface region towards the original liver parenchyma revealed an intermediary hepatobiliary phenotype (Fig. 1D arrows).

Hepatoblastoma most frequently metastasize to the lungs [36]. Therefore, we screened the lungs of 90-week-old Keap1:.Alb-Cre mice for hepatoblastoma manifestations. We found metastases in 64% (12/18) of mice analyzed. Interestingly, two different morphologic variants were seen: 75% (9/12) of metastatic lesions were morphologically similar to the HLN found in the liver (Fig. 1E), whereas the remaining 25% (3/12) revealed a more differentiated phenotype forming cohesive aggregates of tumor cells (Fig. 1F).

### 2.2 Immunohistological characterization of the HLN cells

**Fig. 2:**
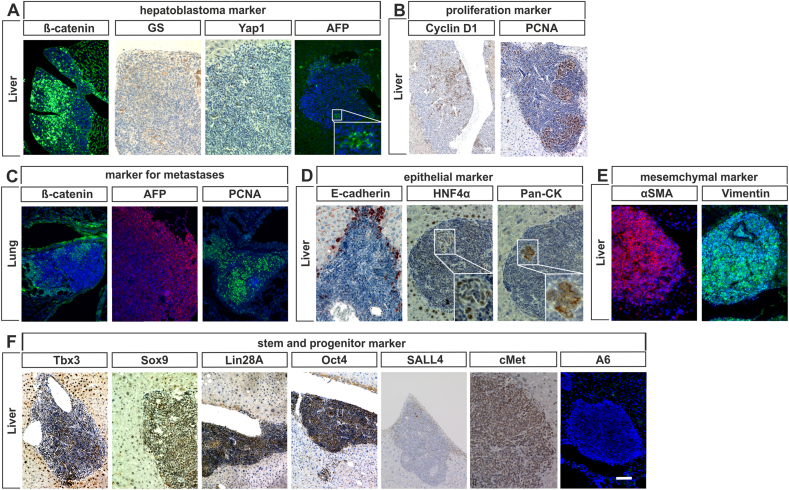
Immunohistochemical characterization of HLN neoplasias. Representative microscopic pictures of liver and lung slices from 90-week-old Keap1:Alb-Cre mice immunostained with antibodies against: (*A*) hepatoblastoma marker; (*B*) proliferation marker; (*C*); marker for metastases (*D*) epithelial marker; (*E*) mesenchymal marker; (*F*) stem and progenitor cell marker. (Scale bar = 50 μm).

The histological appearance suggested that the neoplasms were hepatoblastomas. Therefore, we investigated the expression of β**-**catenin, known to be mutationally activated in the majority of hepatoblastoma [18]. Interestingly, most HLN cells stained positive for β-catenin in both cytosol and nucleus. Also, the β-catenin target gene glutamine synthetase (GS) showed a diffuse upregulation in HLN [37]. Additionally, we detected Yap1 positive cells in the HLN. Wnt/β-catenin is known to act in concert with the Yes-associated protein 1 (Yap1) pathway to induce the development of hepatoblastoma [38]. Moreover, a distinct expression of α-fetoprotein (AFP) was detected in HLN. Together, these findings confirmed the diagnosis of murine hepatoblastoma (Fig. 2A) [38,39].

Next, we performed staining for the cell cycle regulator cyclin D1 and the proliferation marker Proliferating Cell Nuclear Antigen (PCNA) to detect cell proliferation in HLN [40]. Cyclin D1 expression was found in about one third of all HLN cells suggesting that these cells are within the G_1_ phase of the cell cycle. In addition to scattered PCNA-positive cells distributed throughout the neoplasia, we found distinct foci of strongly proliferating cells indicating that tumor heterogeneity may be involved in progression of HLN (Fig. 2B).

As expected the lung metastasis also expressed β-catenin and AFP and a strong PCNA-staining (Fig. 2C).

We assumed that permanent Nrf2 activity might reprogram this WNT-responsive cell population, driving the cells towards an undifferentiated state. Therefore, we analyzed the epithelial-mesenchymal transition (EMT) in HLN, because loss of tissue organization in neoplastic areas was obvious, and lung metastases occurred. One of the earliest stages in, and a hallmark of EMT is the repression of the cell-cell adhesion molecule E-cadherin. While hepatocytes adjacent to HLN exhibited a highly intensified, non-polarized expression of E-cadherin, the hepatoblastoma-like cells were almost entirely negative in the E-cadherin immunostaining (Fig. 2D). To further validate a mesenchymal phenotype two additional mesenchymal markers were analyzed: Vimentin and α-SMA. We found strong expression of both markers in the majority of the HLN cells, indicating a differentiation shift towards a mesenchymal phenotype (Fig. 2E).

Next, we investigated the presence of areas of hepatocellular and cholangiocellular differentiation using consecutive sections. The analysis of hepatocyte nuclear factor 4α (HNF4α) expression, a transcription factor that is essential for maintaining hepatocyte differentiation [41], revealed that Keap1:Alb-Cre hepatocytes showed a nuclear HNF4α staining, with a particularly strong staining at the periphery of HLN. In contrast, almost all of the HLN cells did not express HNF4α. Noteworthy, focal aggregates of cohesively appearing HNF4α positive cells consistent with foci of re-differentiated tumor cells could be detected within HLN (Fig. 2D, center, insert showing a detailed view). To visualize cells with cholangioblastic differentiation among HLN cells, we used an antibody that detects cholangiocyte-specific cytokeratins (pan-CK). Almost all HLN cells were negative for pan-CK, again except for few positive aggregates (Fig. 2D, right side, insert showing a detailed view). Considering that consecutive slices were stained and that the same HLN area was analyzed, we concluded that these were identical aggregates showing expression of both HNF4α and pan-CK thus exhibiting a more differentiated hepatobiliary respectively in tubular structures cholangioblastic phenotype; histological features known from human hepatoblastoma as well.

In addition, we analyzed the expression of stem cell markers in HLN, firstly, we looked at was T-box 3 (Tbx3), an important transcription factor in maintaining pluripotency, which the stem cell population around the central vein is known to express [19]. Interestingly, many of the HLN cells as well as the nearby hepatocytes showed strong Tbx3 staining (Fig. 2F).

Lin-28 Homolog A (Lin28A) is a marker of undifferentiated embryonic stem cells. Its overexpression has been reported to be sufficient to initiate hepatoblastoma development [42]. Immunohistostaining showed a strong expression of Lin28A in HLN while surrounding hepatocytes from the Keap1:Alb-Cre mice were Lin28B-negative (Fig. 2F).

Octamer-binding transcription factor 4 (Oct4, also known as POU5F1) is a transcription factor that is important in maintaining stem cell pluripotency [43]. It also promotes the proliferation of cancer cells [44]. Nrf2 has been shown to inhibit the decrease of Oct4 in embryonic stem cells [45]. Although Keap1:Alb-Cre hepatocytes did not test positive for Oct4, we found strong nuclear expression of it in HLN cells (Fig. 2F).

Next, we looked for cMet, a tyrosine kinase receptor known to be expressed in hepatocytes and hepatic progenitor cells [46]. cMet is strongly expressed in the majority of HLN cells (Fig. 2F).

As previously shown, Nrf2 upregulates the proliferation of oval cells in the liver, we analyzed the expression of the A6 antigen by immunohistochemistry [7]. In HLN no A6-positive cells were detected, suggesting that the undifferentiated state of HLN cells is different from the progenitor state of oval cells (Fig. 2F).

**Fig. S3:**
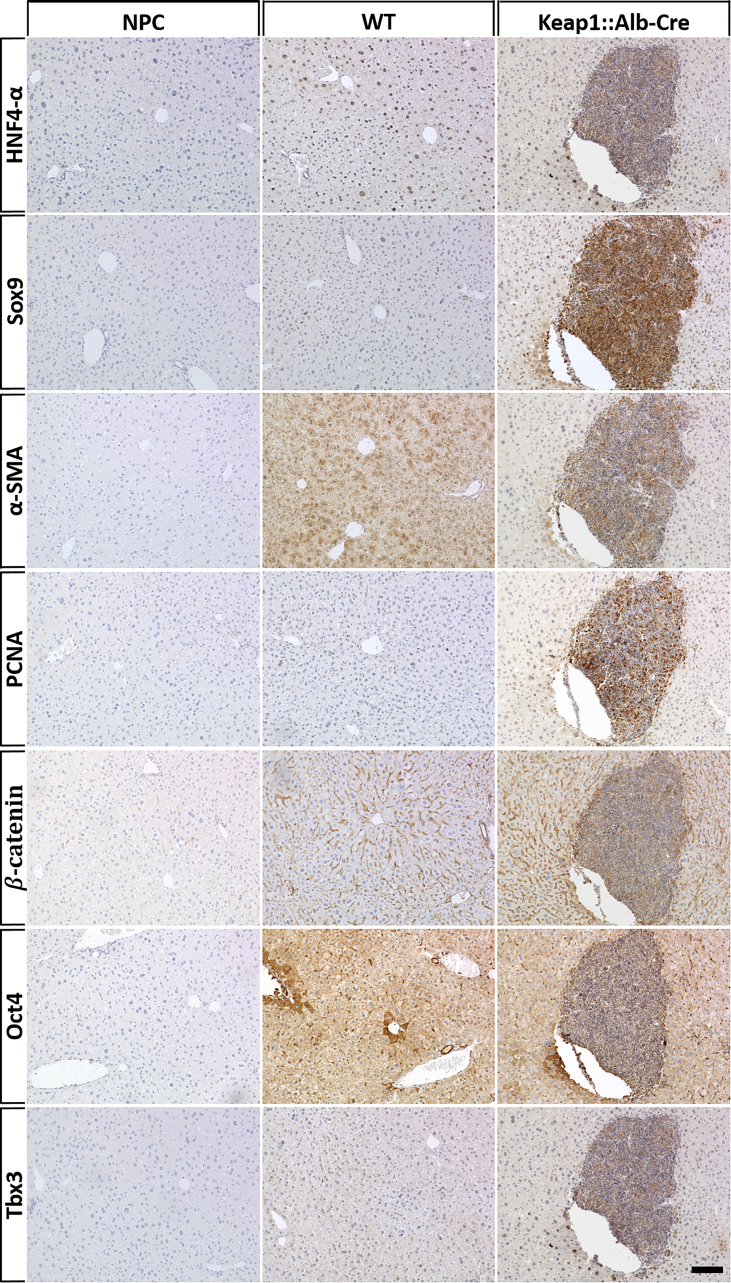
Additional immunohistochemical analyses for representative markers of each category shown in Fig. 2 (epithelia: HNF4-α; stem and progenitor cells: Sox9, Oct4 and Tbx3; mesenchyme: α-SMA; proliferation: PCNA; hepatoblastoma: β-catenin). Due to the use of consecutive sections, these analyses confirm the co-expression of investigated markers in the same HLN.

As mentioned in the material and methods section, it was not possible to demonstrate all investigated markers on one and the same HLN. Therefore, additional IHC staining of representative markers of each category were conducted on consecutive sections of one liver to confirm the co-expression of them in the same HLN (Fig. S3).

In summary, constitutive Nrf2 activation induced proliferation and de- (but also re-) differentiation of a defined stem cell population surrounding the central veins. This process culminated in a malignant transformation. The cells forming HLN show features of various lines of differentiation also present in human hepatoblastoma including undifferentiated, epithelial (embryonal, cholangioblastic), and mesenchymal patterns.

### 2.3 Keap1- and Nrf2-zonation in the liver

**Fig. 3:**
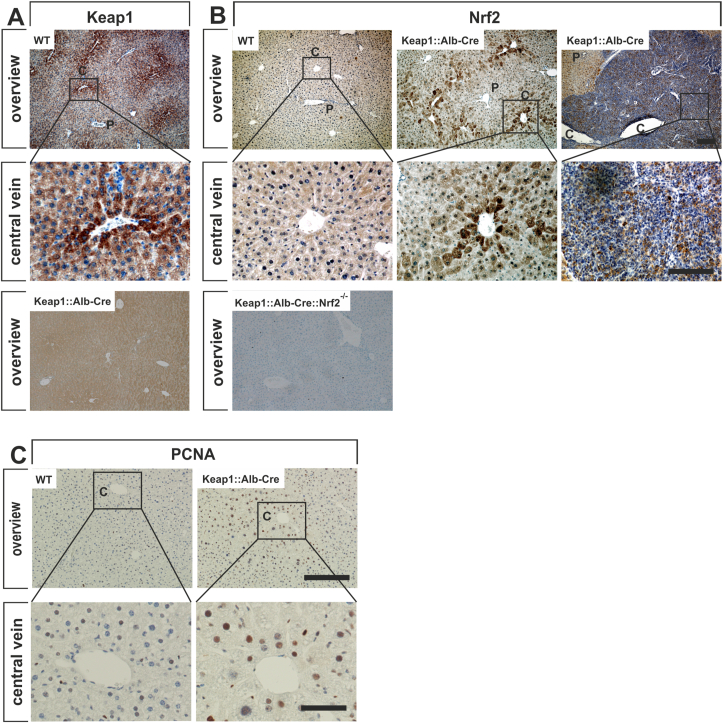
Zonation of Keap1-, Nrf2- and PCNA (proliferation) in the liver. Liver sections from 28-week-old wild-type (WT) and Keap1:Alb-Cre mice were immunohistochemically stained with antibodies against Keap1 (*A*). Liver sections from wild-type (WT), Keap1:Alb-Cre mice and Keap1:Alb-Cre:Nrf2^−/−^ were used for Nrf2 immunohistochemistry (*B*). (Scale bar of top and lower row = 100 μm). Additionally, PCNA expression (proliferation marker) was investigated in livers from WT and Keap1:Alb-Cre mice (*C*). (Scale bar of the top row = 200 μm); lower rows are detail enlargements of the pictures in the upper row. (Scale bar = 50 μm). P = portal field, C = central vein.

The liver lobule is spatially zonated between the portal and central veins. Several crucial signaling pathways exhibit an activity gradient from the portal tract to the central vein [47]. Therefore, we examined the lobular zonation of Keap1 and Nrf2 in WT and Keap1:Alb-Cre mice by immunohistochemistry. As previous studies demonstrated, Nrf2 is stabilized and activated in particular in perivenular hepatocytes, even under unstressed conditions [27]. Surprisingly, we detected a strong Keap1 expression in perivenular hepatocytes, while there was almost none in the periportal parenchyma of WT mouse livers (Fig. 3A). However, Nrf2 expression was below detectable levels in the entire liver lobule of WT mice (Fig. 3B, first column). In the absence of Keap1, Nrf2 expression was clearly detectable in the cytosol and in the nucleus of perivenular hepatocytes, whereas the hepatocytes in the periportal field showed only occasional Nrf2 expression (Fig. 3B, second column). These results clearly indicate a Keap1-independent, mechanism regulating a zonal hepatic Nrf2 expression. As expected, HLN cells were largely immunoreactive for Nrf2. To ensure specificity of the Keap1 and Nrf2 immunostainings, we stained Keap1:AlbCre and Keap1:AlbCre:Nrf2^−/−^ mouse liver sections with antibodies against Keap1 and Nrf2, respectively. As shown in the last row of Fig. 3 A and B, no specific staining of Keap1 or Nrf2 can be seen, indicating a complete knockout of these proteins in both hepatocytes and cholangiocytes.

The increased Nrf2 activity of perivenular hepatocytes had functional consequences. Using PCNA immunostaining, we were able to demonstrate that perivenular hepatocytes proliferate in livers of Keap1:Alb-Cre mice, which was not the case in WT livers (Fig. 3C).

### 2.4 β-catenin expression levels and patterns depend on Nrf2 genotypes

**Fig. 4:**
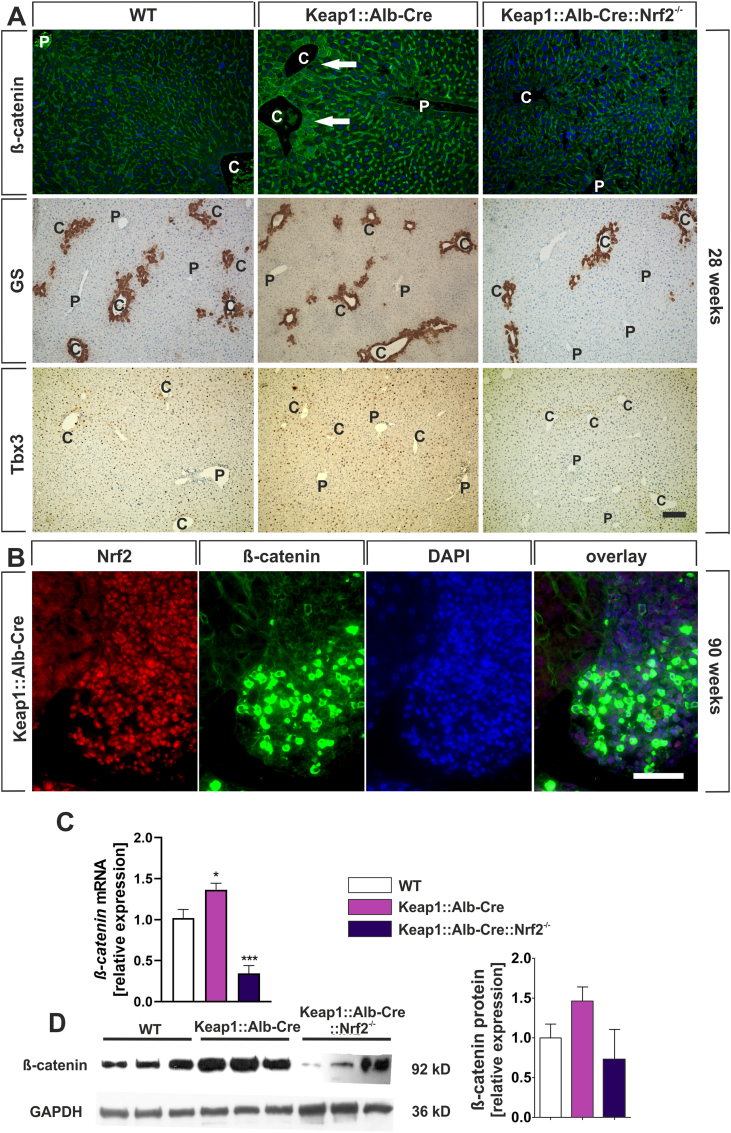
β-catenin expression levels and patterns depend on Nrf2 genotypes. (*A*) Liver sections from 28-week-old wild-type (WT), Keap1:Alb-Cre and Keap1:Alb-Cre:Nrf2^−/−^ mice were stained by immunohistochemistry or -fluorescence using antibodies against β-catenin (upper row), glutamine synthetase (GS, middle row) and Tbx3 (lower row). (Scale bar = 100 μm). Arrows indicate aberrant expression of β-catenin. P = portal field, C = central vein. (*B*) Nrf2 (red) and β-catenin (green) cellular localization was detected using immunofluorence staining performed on liver sections from 28-week-old Keap1:Alb-Cre mice. Nuclei were counterstained with DAPI. (Scale bar on the left row = 50 μm). (*C*) qRT-PCR analysis of *β-catenin* mRNA expression in liver homogenate of 28-week-old wild-type (WT), Keap1:Alb-Cre and Keap1:Alb-Cre:Nrf2^−/−^ mice. Raw expression data were normalized to *Sdha* and *Gapdh* expression data, and data sets were related to WT. Data represent mean ± SEM; (biological replicates n = 5); *p < 0.05, ***p < 0.001 vs. WT; one way ANOVA and Dunnett post hoc test (*D*) Western blot analysis of whole liver extracts for β-catenin (92 kD) protein expression from WT-, Keap1:Alb-Cre-, and Keap1:Alb-Cre:Nrf2^−/−^ mice. GAPDH (36 kD) was used as internal loading control. Quantification of band densities were determined using QuantityOne software and the values of ß-catenin were related to the loading control ß-actin. Optical density values are mean ± SEM, n = 3. (For interpretation of the references to color in this figure legend, the reader is referred to the Web version of this article.)

As dysregulated β-catenin expression is a hallmark of hepatoblastoma [48], we further analyzed the expression and activity of β-catenin in each genotype. β-catenin localization was determined by immunofluorescence in 90-week-old mice. One of the hints for a mechanistic link between Nrf2 and β-catenin signaling emerged from the cellular localization of β-catenin in the liver parenchyma of Keap1:Alb-Cre mice. Under physiological conditions, transcriptionally inactive β-catenin is immobilized at the cell membrane of hepatocytes by E-cadherin, as seen in WT livers (Fig. 4A, first row first image) or as described in the review by Russel and Monga [18]. In contrast, Nrf2 hyper-activated hepatocytes around central veins exhibited cytosolic and nuclear overexpression of β-catenin suggesting Wnt signaling activation (Fig. 4A, first row second image). Knockout of Nrf2 in Keap1:Alb-Cre mice restored the physiological β-catenin expression pattern and further confirmed a possible impact of Nrf2 on β-catenin expression (Fig. 4A, first row third image).

Next, we investigated whether this aberrant expression pattern of catenin affects the expression of catenin target genes. For this purpose we performed immunohistochemical staining for the β-catenin targets GS and Tbx3 (Fig. 4A, second and third row). Contrary to our expectations, we could not detect any significant difference in the expression of these cate β-catenin target genes. This may be due to the fact that our method of immunohistochemistry does not reveal the slight differences in the expression of these β-catenin target genes.

It should be noted that both the HNL cells and the surrounding hepatocytes upregulate Tbx3 expression (Fig. 2F). This allows us to assume that Nrf2 increases the expression of Tbx3, perhaps by modulating β-catenin signaling.

The accumulation of cells with active β-catenin in HLN was particularly pronounced (Fig. 4B). Based on these findings, we explored whether Nrf2 and β-catenin were co-localized in HLN nuclei. Both Nrf2 and β-catenin exhibited nuclear co-localization in nearly all HLN cells. β-catenin exhibited strong accumulation in the cytosol and weak but clear accumulation in the nucleus (Fig. 4B).

**Fig. S4:**
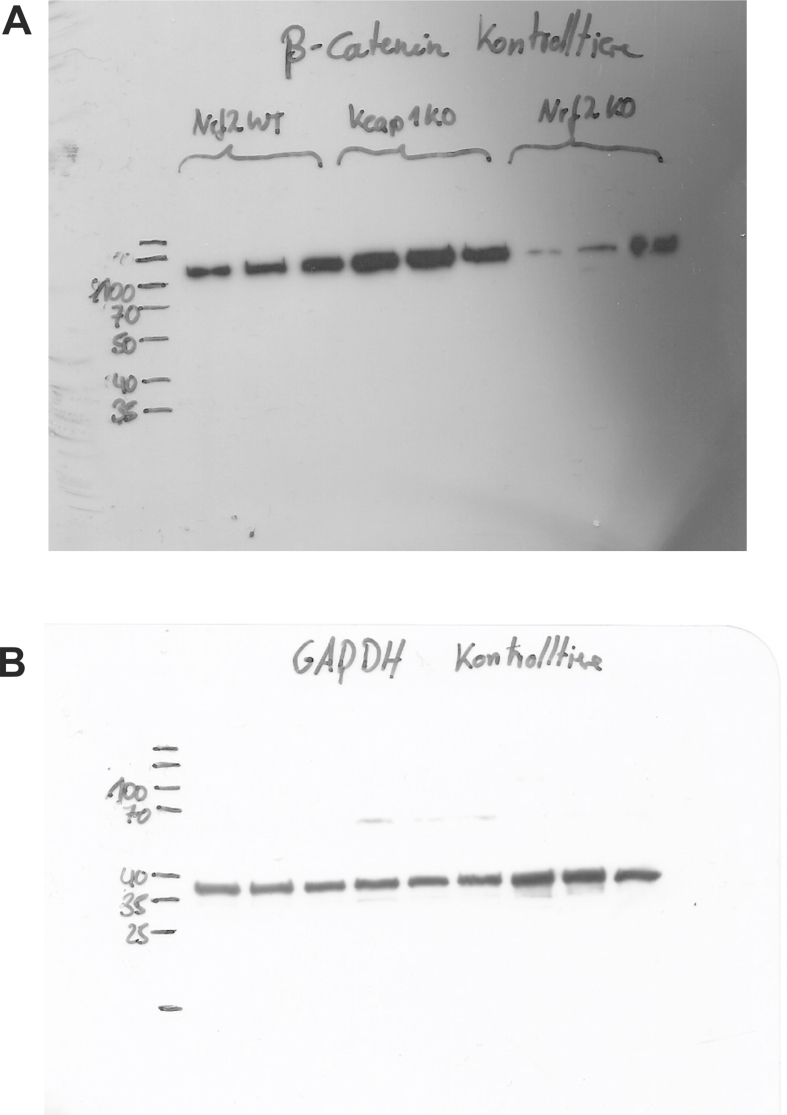
*(A)* Full blot images of whole liver extracts for β‑catenin (92 kD) protein expression from WT (Nrf2 WT), Keap1::Alb-Cre (Keap1 KO), and Keap1::Alb-Cre::Nrf2^-/-^ (Nrf2 KO) mice. (B) Full blot images for GAPDH (36 kD) protein expression used as loading control.

Next, we analyzed β-catenin expression at the mRNA and protein levels from livers of 90-week-old Nrf2-overactive (Keap1:Alb-Cre), Nrf2-knockout (Nrf2^−/−^), and wild-type mice by real time RT-PCR and Western blotting. These analyses showed an Nrf2-dependent expression of β-catenin mRNA. In Keap1:Alb-Cre livers the β-catenin expression was significantly increased (1.4-fold) and significantly decreased in Nrf2^−/−^ livers (0.3-fold) compared to their wild-type littermates (Fig. 4C). This finding was basically confirmed at protein level (Fig. 4D and Fig. S4).

### 2.5 Nrf2 induces β-catenin expression

**Fig. 5:**
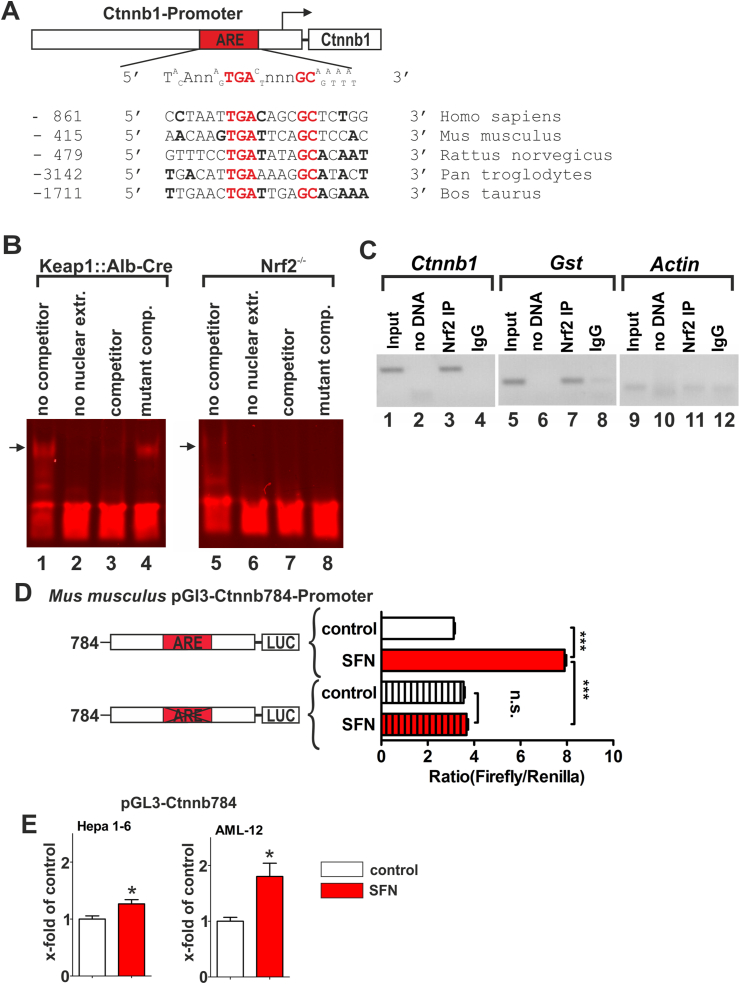
Nrf2 binds the ARE-sequence in the Ctnnb1 promoter. (*A*) *In silico* analysis on ARE sequences in the β-catenin (gene name: Ctnnb1) promoter of different species. Essential ARE core sequences are highlighted in red. Nucleotides matching the ARE consensus sequence are bolded. (*B*) Infrared-based EMSA approach using nuclear extracts from Keap1:Alb-Cre- and Nrf2^−/−^-hepatocytes. A fluorophore (IR700)-labeled construct of the ctnnb1 promoter region, containing the ARE sequence and an unlabeled version (as competitor) were incubated with a nuclear protein extract of Keap1:Alb-Cre- and Nrf2^−/−^-hepatocytes. Lane 1 sample without competition, a shift was detected in the Keap1:Alb-Cre sample (black arrow), which was absent for Nrf2^−/−^ hepatocytes (lane 5). Lane 2 & 6 are controls without nuclear extract. To test specificity an intact (lane 3 and 7) and a mutant competitor (lane 4 and 8) were used. (*C*) A ChIP assay was performed with samples from Keap1:Alb-Cre hepatocytes. Separated DNA fragments were analyzed by PCR technique targeting the Ctnnb1 promoter. Glutathione S-transferase (Gst) promoter (containing a functional ARE sequence) served as positive control; the Actin promoter (without a functional ARE sequence) functioned as negative control. Input sample represents the DNA fragment pool prior to the pull-down process. Nrf2-IP are samples after the pull-down with an Nrf2 antibody. The IgG sample was incubated with an unspecific antibody that served as a pull-down negative control. Product size: Ctnnb1 (126 bp), Gst (120 bp), Actin (80 bp). (*D*) iHep-dKeap1 hepatocytes were co-transfected with vectors carrying a murine Ctnnb1 promoter reporter (*firefly* luciferase) construct, consisting of the ARE sequence (Ctnnb1-784) and a mutant version without functional ARE sequence (Ctnnb1-784-ΔARE) combined with phRL-TK vector for *Renilla* luciferase co-expression. Nrf2 was induced by 2,5 μM sulforaphane (SFN) treatment for 6 h. *Firefly* Luciferase activity was measured and normalized by *Renilla* activity. (*E*) Hepa 1–6 and AML-12 cells were co-transfected with vectors carrying the Ctnnb1-784 construct and phRL-TK. Nrf2 was activated with 2,5 μM SFN for 6 h. A one-way ANOVA test followed by a Bonferroni post-hoc test for selected groups were used. Results represent mean ± SEM, (biological replicates n = 5), n.s. = not significant, *: p < 0.05 vs. control, ***: p < 0.001 as indicated. (For interpretation of the references to color in this figure legend, the reader is referred to the Web version of this article.)

The ARE consensus sequence consists of two main binding motifs (TGA and GC) separated by four random nucleotides [49]. An *in silico* analysis indicated the existence of putative Nrf2 binding sites within the promoter region of the *β-catenin* (*Ctnnb1*) gene in vertebrates (*Homo sapiens*, *Mus musculus*, *Rattus norvegicus*, *Pan troglodytes* and *Bos taurus*) (Fig. 5A). We performed reporter gene assays as well as EMSA and ChIP experiments to validate the functionality of the ARE site in the promoter of the murine *ctnnb1* gene (at position −415).

For the EMSA experiment, we used a fluorophore-labeled (double stranded 5’ modification with IRDye700) construct of the ctnnb1 promoter region containing the ARE sequence and the 20 flanking nucleotides. An unlabeled unmodified sequence served as a competitor control oligonucleotide. Moreover, we applied an oligonucleotide that contained a mutated ARE consensus sequence, which dealt as non-competing mutant competitor control oligonucleotide. Oligonucleotides were incubated with nuclear protein extracts from primary Keap1-deficient hepatocytes (pHep-dKeap1) (Fig. 5B line 1 to 4) or pHep-dNrf2 (isolated from Nrf2^−/−^ mice; Fig. 5B line 5 to 8). Nuclear extracts from pHep-dNrf2 served as a negative control.

Incubation of the ctnnb1 oligonucleotide with pHep-dKeap1 nuclear extracts without competitor induced complex formation indicated by an arrow (Fig. 5B lane 1). In contrast, this shift was not observed when incubating nuclear extracts from Nrf2-deficient pHep-dNrf2 cells with ctnnb1 oligonucleotide (Fig. 5B lane 5). This difference indicates that the observed shift is due to a specific binding of Nrf2 to the *ctnnb1* promoter. To test for specificity to ctnnb1, competition experiments were performed using an intact and an mutant competitor, which results in a shift disappearance as well as a persistent complex formation in case of the mutated competitor (Fig. 3, Fig. 5 + 4 lane).

In addition, we performed ChIP experiments using pHep-dKeap1 to investigate the *in vivo* binding of Nrf2 to the *Ctnnb1* promoter. Therefore, all Nrf2-bound genomic DNA was pulled down and the obtained DNA fragments were subjected to subsequent PCR analysis with a specific primer targeting the ARE-containing region of the *Ctnnb1* promoter (Fig. 5C lane 1 to 4). Primers spanning the ARE sequence of the *glutathione S-transferase* (GST) promoter served as a positive control (Fig. 5C lane 5 to 8); primers corresponding to the *actin* promoter without ARE sequences served as a negative control (Fig. 5C lane 9 to 12). First, the control approaches clearly proved the specificity of the assay. While there was no PCR product for the ARE-lacking *actin* promoter, the ARE-containing *Gst* promoter region was clearly detected in the Nrf2-IP sample. The same was true for the *Ctnnb1* promoter, for which we detected a specific PCR product in the Nrf2-IP sample.

To intensify the promoter analyses, we also performed a dual luciferase reporter gene assay with immortal Keap1-deficient hepatocytes (iHep-dKeap1) cells. For this purpose, we co-transfected a pGL3-luciferase reporter gene-based construct containing parts of the murine *Ctnnb1* promoter (−784 to +1) that exhibit either an intact or a mutated ARE site with the internal control vector known as phRL-TK.

The ARE sequence is located −408 to −399 bp from the transcription start site (Fig. 5A). To induce Nrf2 activity, transfected cells were stimulated with 2.5 μM sulforaphane (SFN) for 6 h. Luciferase activity was measured thereafter. In cells transfected with the pGL3 ctnnb1-784 (with an intact ARE site), SFN treatment resulted in a significant increase of promoter activity (ratio Firefly/Renilla = 8) compared to the vehicle treated control (ratio Firefly/Renilla = 2.7). The second vector contained a dysfunctional ARE sequence caused by mutation of the TGA binding site (pGL3 ctnnb1-784-ΔARE). Cells transfected with this mutant ARE construct showed no increase in promoter activity after SFN stimulation (Fig. 5D). The intact *Ctnnb1* promoter construct was also tested in the established hepatic cell lines Hepa1-6 and AML-12 to verify our results obtained from iHep-dKeap1 cells (Fig. 5E).

Taken together, these data provide evidence that the *ctnnb1* promoter contains a functional ARE site to which Nrf2 is able to bind, resulting in transcriptional activation of β-catenin expression.

### 2.6 Keap1-knockout induces persistent proliferation and de-differentiation

**Fig. 6:**
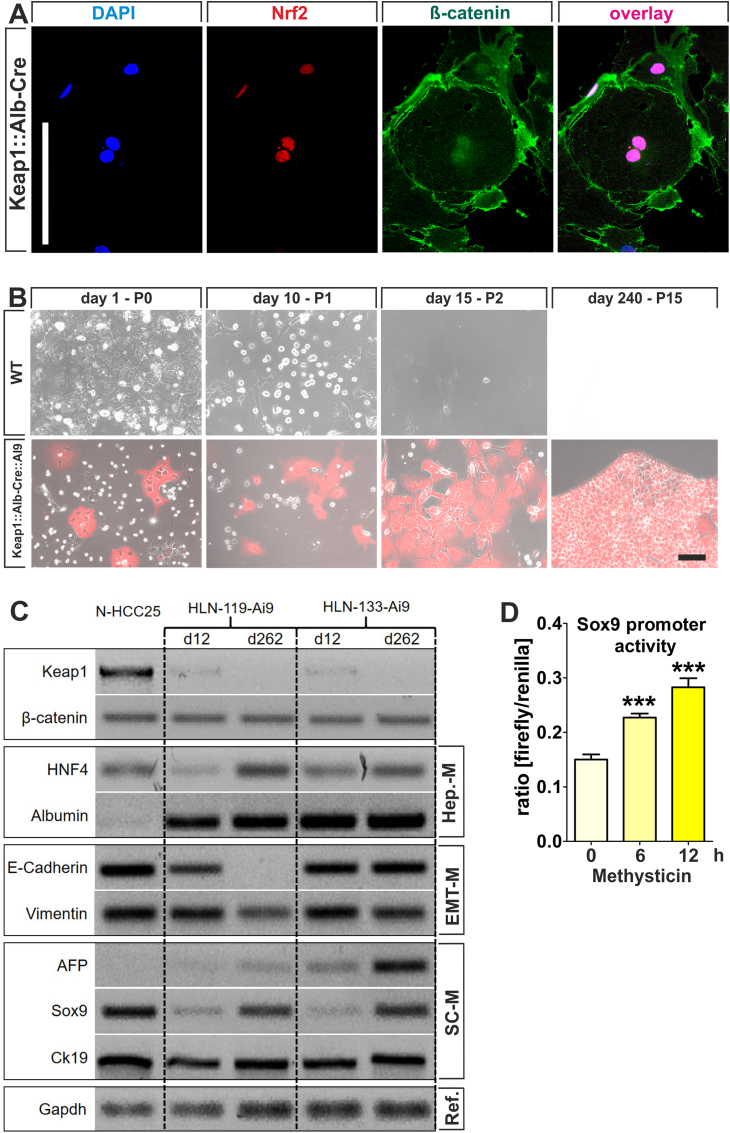
Characterization of cultivated Keap1:Alb-Cre hepatocytes. (*A*) Double immunofluorescence staining of Nrf2 (in red) and β-catenin (in green) from cultivated Keap1:Alb-Cre hepatocytes (iHep-dKeap1). Both Nrf2 and β-catenin were localized in the cell nucleus with additional β-catenin at the cell membrane. Nuclei were counterstained with DAPI. (Scale bar = 100 μm). (*B*) Primary hepatocytes isolated from 90-week-old wild-type (WT) and Keap1:Alb-Cre:Ai9 mice were cultivated; morphology was determined by phase contrast and Cre activity by fluorescence microscopy. Wild-type hepatocytes survived for a two-week period in culture (upper row). In contrast, cultivated Keap1:Alb-Cre:Ai9 hepatocytes survived for more than 240 days (lower row). (Scale bar = 50 μm). (*C*) Gene expression profiles for genes associated with hepatocyte origin (*Hnf4*, *Albumin*), EMT (*E-cadherin*, *Vimentin*) and LPC origin (*Afp*, *Sox9*, *Ck-19*) were analyzed in HLN-cells derived from Keap1:Alb-Cre:Ai9 mice (HLN-119-Ai9 and HLN-133-Ai9) cells via RT-PCR. (*D*) HLN-133-Ai9 cells were co-transfected with a vector carrying a human Sox9 promoter-driven *firefly* luciferase in combination with a phRL-TK vector for *Renilla* co-expression. Nrf2 was activated with 2,5 μM Methysticin for indicated time. A one-way ANOVA test followed by a Bonferroni post-hoc test for selected groups were used. Results represent mean ± SEM, (biological replicates n = 3), ***: p < 0.001. (For interpretation of the references to color in this figure legend, the reader is referred to the Web version of this article.)

The co-expression and nuclear co-localization of Nrf2 and β-catenin in iHep-dKeap1 cells was confirmed *in vitro* suggesting that our *in vitro* system may be useful for detailed analyses (Fig. 6A).

In particular, the fluorescence microphotographs of primary hepatocytes showed that only hepatocytes isolated from Keap1:Ai9:Alb-Cre mice clearly expressed the tdTomato fluorophore; cells isolated from WT did not (Fig. 6B). This indicates that the Keap1:Ai9:Alb-Cre hepatocytes express a Cre-recombinase and therefore qualify as Keap1 knockout.

Primary WT hepatocytes are known to have a restricted proliferation and survival time, which is usually limited to a few cell divisions [50]. In the present study WT cells could not be cultivated longer than 15 days (Fig. 6B upper row). Fascinatingly, Keap1:Alb-Cre hepatocytes, which were isolated from 90-week-old Keap1:Ai9:Alb-Cre mice, totally lost proliferation control and appeared virtually immortal (Fig. 6B, lower row).

The resulting cell line (HLN133-Ai9) was Keap1-deficient as indicated by the continuous expression of the Cre-dependent tomato fluorophore. Subsequently, this and a second cell line (HLN119-Ai9), which was also isolated from another Keap1:Ai9:Alb-Cre mouse, was analyzed for the expression of cell type specific markers by absence-presence RT-PCR (Fig. 6C). N-HCC25 cells, which originate from a NASH-HCC mouse model, served as reference for the investigated expression patterns. GAPDH serves as reference gene [51]. Here, we examined the expression of these markers after different durations of sub-cultivation (12 and 262 days after isolation). In both cell lines, *Keap1* mRNA could only be detected initially and at a very low level, maybe due to cross-contamination with other cell types such as fibroblast that might be involved in these early cultures. However, after 262 days of cultivation, *keap1* mRNA was no longer detectable. Additionally, al investigated cell lines expressed *ß-catenin*, but there was neither a difference observable between the cell lines nor time points. The expression of *HNF4α* and *albumin* was used to prove the cell's hepatocytic origin. *E-cadherin* and *vimentin* expression are indicators for a possible EMT. Analysis revealed that only the HLN119-Ai9 cell line loses the initial expression *of e-cadherin* and *vimentin* over time as would be expected after the EMT process. Further analyses on LPC markers revealed that both cell lines initially lacked or only slightly expressed *afp* and *sox9* in the early culture, but induced their expression in the following sub-culturing. At day 262 all three investigated LPC markers (*afp*, *sox9* and *ck-19*) were highly expressed, which was even more pronounced in the HLN-133-Ai9 cell line, indicating a possible dedifferentiation of these cells. Since Sox9 has been described as a crucial factor in liver progenitor cells regulated by both Nrf2 [31] and ß-catenin [52], we additionally conducted a reporter gene assay using a Sox9 promoter-driven luciferase construct und methysticin treatment to induce Nrf2 activity. Methysticin showed a time-dependent induction of Sox9 promoter activity after 6 h and even more pronounced after 12 h of treatment, indicating a possible transcriptional regulation of Sox9 by Nrf2 (Fig. 6D). These data showed that Keap1-knockout in hepatocytes induces persistent proliferation and de-differentiation.

### 2.7 Nrf2 knockdown decreased β-catenin expression and proliferation and initiated redifferentiation

**Fig. 7:**
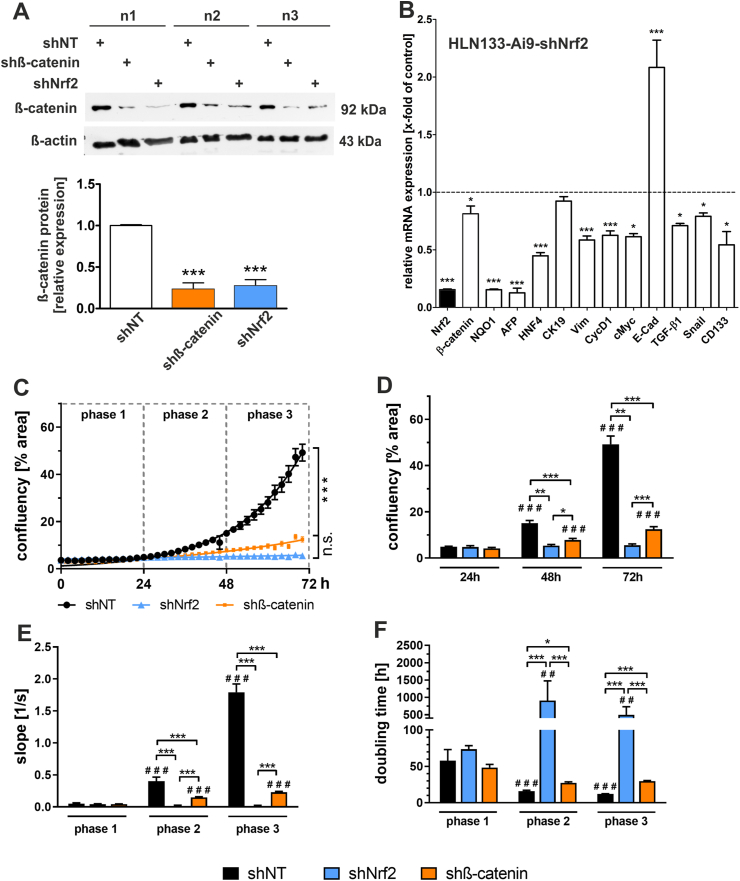
shRNA-mediated knockdown of Nrf2 or ß-catenin stops HLN-133-Ai9 cell de-differentiation and proliferation. (*A*) Western blot analysis of ß-catenin protein expression after shRNA-mediated Nrf2 or ß-catenin knockdown. Lentiviral shRNA delivery was conducted in three independent experiments (n = 3). Band densities were determined using QuantityOne software and the values of ß-catenin were related to the loading control ß-actin. Data were normalized to the biological control shNT and plotted as relative expression. Data = mean ± SEM; one-way ANOVA followed by Bonferroni selected multiple comparison post hoc testing; ***p < 0.001 vs. shNT. (*B*) RT-qPCR analysis of markers for the differentiation status of HLN133-Ai9 cells transduced with shRNA against Nrf2 (shNrf2). HLN133-Ai9 cells were treated with non-target shRNA (shNT) for control (represented as dashed line). data represent mean ± SEM. one-way ANOVA followed by the Tukey multiple comparison post-hoc test. * p < 0.05 & ***p < 0.001 vs. shNT. (*C*) Incucyte® proliferation analysis was performed with cultured HLN133-Ai9 cells that were transduced with shRNA either targeting Nrf2- or β-catenin mRNA or transduced with the shNT control construct. n = 3, data represent mean ± SEM. Real-Time course of the Incucyte measurement of cell confluence over a period of 72 h. Two-way repeated-measures ANOVA followed by the Tukey multiple comparison post-hoc test. n.s. = not significant, *** p < 0.001 as indicated. (*D*) Endpoint analyses of cell confluency at the indicated time points (24, 48 and 72h), (*E*) slope analyses and (*F*) doubling time analyses of indicated growth phases. Mixed-effects two-way repeated-measures ANOVA followed by the Tukey multiple comparison post-hoc testing. # # # p < 0.001 vs. same experimental group at 24h time point or phase 1 respectively; * p < 0.05, ** p < 0.01 and *** p < 0.001 as indicated by the brackets.

Using shRNAs against Nrf2 (shNrf2) and β-catenin (shβ-catenin) as well as non-target control shRNA (shNT), we sought to specifically knock down these two factors in the Nrf2-hyperactivated Keap1:Ai9:Alb-Cre hepatocytes (HLN133-Ai9) to investigate the effect on their proliferative behavior. Therefore, the cells were transduced in three independent experimental approaches designated as n1, n2 and n3 in Fig. 7A. For a better measurement of the WB bands, they were subjected to densitrometric analysis, and statistically evaluated (Fig. 7A). Each first line of n1–n3 shows the control experiment with non-target shRNA (and thus the initial amount of the target protein). Each second line of n1–n3 showed that the shRNA targeting *β-catenin* decreased *β-catenin* protein expression by 76%. Interestingly, the knockdown of Nrf2 (third line of n1–n3) was also accompanied by significantly decreased protein expression of β-catenin, which was comparable to β-catenin shRNA with a knockdown efficiency of 59% (Fig. 7A). This result once more hints that Nrf2 may play a role in regulating β-catenin expression.

Additional RT-qPCR experiments further strengthened our finding that the Nrf2/β-catenin axis might have an essential impact on HLN cell fate and behavior. shRNA targeting Nrf2 decreased the mRNA expression of *Nrf2*, of the established Nrf2 target gene *Nqo1* and similar to our results presented in Fig. 7A of *β-catenin*. We were able to show that the shRNA had distinct effects on several markers that we analyzed before (Fig. 7B, also compare Fig. 6C). shRNA targeting *Nrf2* reduced the expression level of *Hnf4α,* *Afp,* *Vimentin,* *CyclinD1,* *Cmyc,* *Tgfβ1,* *Snail* and *Cd133*. Such Nrf2 knockout-induced reduction in the expression of these markers is consistent with our assumption that Nrf2 has a direct effect on the differentiation status of these cells (Fig. 7B).

Once we had ascertained that the applied shRNAs had been effective, we performed a cell confluence-based proliferation assay over a period of 72h to measure the effect of β-catenin and Nrf2 knockdown on proliferative capacity. Similar to malignant tumor cells, Nrf2 hyperactive HLN133-Ai9 cells transduced with non-target shRNA (control shRNA) showed an exponential growth (Fig. 7C). This growth was significantly inhibited by knock down of either Nrf2 or β-catenin (Fig. 7C). For a detailed analysis, the obtained longitudinal data set was further analyzed. Therefore, we defined specific time points (Figs. 7D, 24 and 48 and 72h) for end point analyses of cell culture confluence, which simultaneously defined three distinct growth phases (Fig. 7E and F, phase 1–3) for slope as well as doubling time analyses. These analyses showed that Nrf2- and β-catenin knockdown-mediated growth inhibition was detectable as early as 48h after seeding, or that the inhibition already started wtihin growth phase 2. This is evidenced by the significantly reduced confluence and slope (Fig. 7D and E) and increased doubling time (Fig. 7F) of shNrf2 (blue bar) or shβ-catenin respectively (orange bar) compared to shNT cells (black bar). These effects were even more pronounced after 72h or in phase 3 respectively. It was also striking that these effects were significantly more profound in shNrf2 transduced cells than in shβ-catenin cells (Fig. 7D–F, blue vs. orange bar).

These *in vitro* data clearly showed that permanent Nrf2 activity induces hepatocyte proliferation and that β-catenin plays a crucial role in this process.

### 2.8 Nrf2 induced β-catenin expression is the main driver of HLN development

Thus, we confirmed that Nrf2 induces expression of β-catenin, and found an accumulation of both factors in HLN, we wanted to determine how the Nrf2–β-catenin axis affects tumor initiation, progression and outcome.

For this purpose we generated two different mouse lines. One with a liver-specific knockout for both Keap1 and β-catenin (Keap1:β-catenin:Alb-Cre); the other with a liver-specific knockout for Keap1 and total Nrf2 knockout (Keap1:Alb-Cre:Nrf2^−/−^). To determine the frequency of HLN, we quantified the livers of 28-week-old WT-, Keap1:Alb-Cre-, Keap1:β-catenin:Alb-Cre- and Keap1:Alb-Cre:Nrf2^−/−^-mice based on H&E histology after standardized sampling of the entire left lateral lobe.

**Fig. S5:**
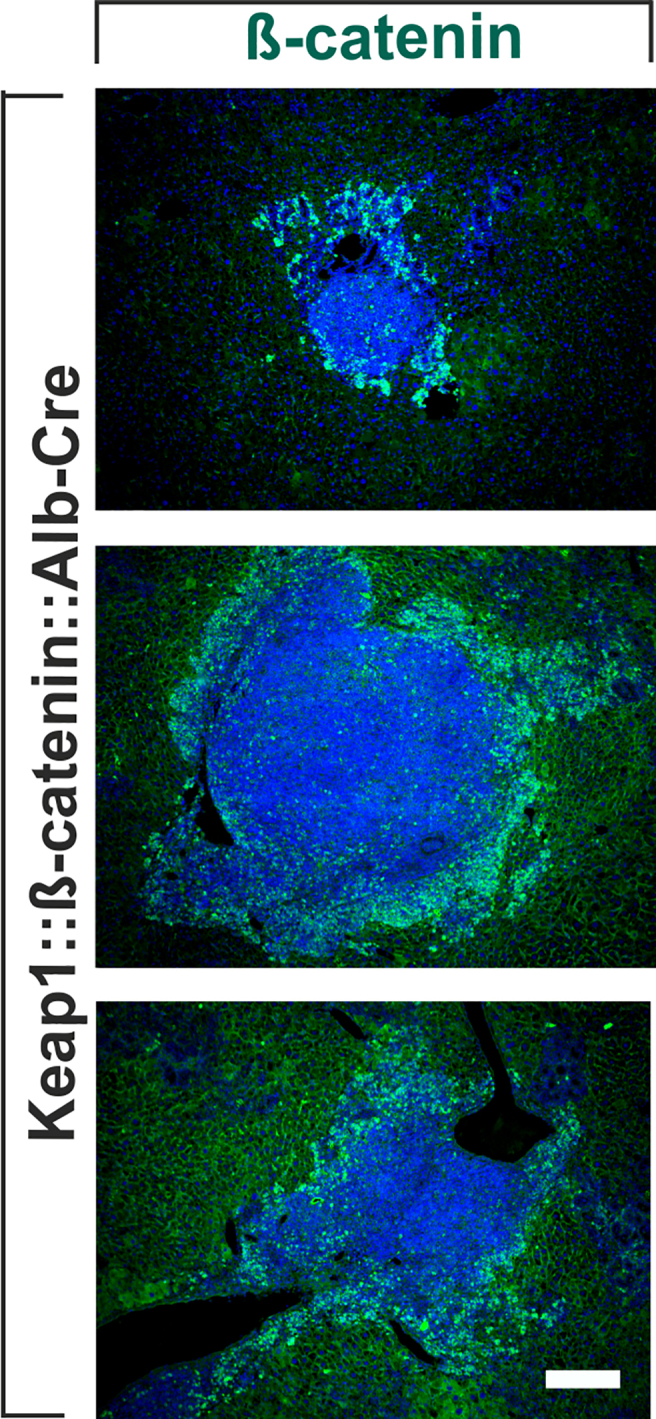
Liver sections from Keap1::β‑catenin::Alb-Cre mice were immunofluorescence-stained with antibodies against β‑catenin. (Scale bar = 200 µm).

Both WT- and Keap1:Alb-Cre:Nrf2^−/−^-mice were free from HLN. This finding clearly showed that over-activated Nrf2 induces HLN. In screening the Keap1:β-catenin:Alb-Cre livers, we obtained an initially unexpected result, as small but distinct neoplastic lesions were present in 3 out of 7 mice. This result did not show the anticipated clear association between Nrf2 and β-catenin signaling; indeed, almost half of the Nrf2-hyperactive mice developed HLN despite the absence of β-catenin. As reported by Sekine et al. in 2011, β-catenin-positive hepatocytes repopulate pericentral areas of older β-catenin:Alb-Cre mouse liver [53]. We therefore checked whether neoplasms in Keap1-β-catenin double-knockout mice might re-express β-catenin. Indeed, immunofluorescence staining confirmed β-catenin expression in all HLN found in Keap1:β-catenin:Alb-Cre mice (Supplementary Fig. 5), suggesting that β-catenin is essential for the initiation of HLN. In the Keap1:Alb-Cre mice, HLN lesions were present in 6 out of 7 animals (Table 1).

## 3 Discussion

Nrf2 has been described as a tumor suppressor due to its cytoprotective functions and is the most important cellular defense mechanism against exogenous and endogenous influences [54]. However, several recent genomic analyses have identified somatic gain-of-function mutations in the genes encoding for Keap1 or Nrf2. The molecular mechanisms underlying this Janus-headed character are still unknown. Our work offers a new perspective on the role of Nrf2 in stem cell regulation and cancer initiation. We report that continuous Nrf2 activation induces dedifferentiation and proliferation of a Wnt-responsive stem cell population located at the central veins of the liver. Nrf2 transforms these cells into invasively growing tumor cells, exhibiting features of human hepatoblastoma that metastasize into the lungs. While most lesional cells display an undifferentiated phenotype and reveal expression of stem cell markers (e.g. *tbx3, oct-4, lin28a*), there is evidence of foci showing either mesenchymal (*vimentin, α-sma, vimentin*) or epithelial differentiation. The latter may show an embryonal (HNF4α, AFP) or a cholangioblastic (pan-CK) pattern. Overall, the histology of hepatoblastoma-like neoplasia (HLN) reflects the heterogeneity known from human hepatoblastoma [55]. Regarding tumor development it should be noted that E-cadherin is able to inhibit Nrf2 activity and HNF4α can block β-catenin activity [28,56], explaining partial differentiation of HLN cells; a phenomenon known from many blastemal human tumor (in particular following chemotherapy).

Mechanistically, the strict perivenular location of HLN suggests that permanent Nrf2 activity leads to a reprogramming of the Wnt-responsive perivenular stem cell population primarily resulting in undifferentiated HLN. To address the question why the scenario is strictly limited to the stem and progenitor cell population at the central veins, we first focused on the zonation of Keap1 and Nrf2 in the hepatic lobules. In wild-type mice, the most intense Keap1 expression was found in the first layer of cells surrounding the central veins, which is also known to contain active β-catenin as suggested by restricted physiological GS expression in these cells. Nrf2 expression was undetectable throughout the hepatic lobule, suggesting a redox-stress-free environment in the wild-type liver. This clearly indicates a strict gradient of Nrf2 regulation across the hepatic lobule. Nrf2 appears to be regulated primarily by Keap1 at the central veins; this is surprising enough given the low oxygen pressure of this area. Other mechanisms, such as GSK3/β-TrCP at the parenchyma and portal field, also appear to regulate Nrf2 [27,57,58]. The assumption of a gradient of Nrf2 activation across the hepatic lobule is also supported by the finding that stabilized, active Nrf2 was found primarily at the central vein but not in the remaining parenchyma. These locally restricted Nrf2 activation leads to a proliferation of perivenular hepatocytes in Keap1-knockout mice.

Next, we deciphered the molecular mechanisms that enables Nrf2 to induce this process of dedifferentiation and proliferation. In previous studies, we have shown that Nrf2 can induce stem cell proliferation in muscle and liver to initiate and support tissue regeneration [6,7]. Research from the Nusse Laboratory has described a population of Wnt-responsive hepatocytes with stem cell characteristics surrounding the central veins [19]. In this context, it was obvious that a factor from the Wnt pathway might be involved and, in line with our findings, regulated by Nrf2. In fact, our data clearly demonstrated that β-catenin is upregulated directly by Nrf2. We could show that the promoter region of the gene for β-catenin in *Mus musculus* contains a complete ARE consensus region. This ARE sequence is also found in the human genome except for one base (Fig. 5 A), which suggests that the data presented here could also be transferred to humans. What do we already know about the interaction between the Nrf2 and Wnt pathways at the molecular level? The activation and inhibition of both factors seems interwoven. A complex of E-cadherin-bound β-catenin directly interacts with and inhibits Nrf2, whereas stimulating hepatocytes with Wnt-3A (an activator in canonical Wnt signaling) can activate the Nrf2 pathway [27,28,59]. This cooperation makes sense, because tissue homeostasis and regeneration involves both pathways.

Various clinical studies have analyzed genomic data sets from human hepatocellular carcinoma (HCC) and hepatoblastoma biopsies. Overall, they indicate a collaboration between Nrf2 and the Wnt/β-catenin signaling pathways in human cancers [11,21,22,24,60]. Mechanistic studies in animal models of liver carcinogenesis described a synergistic effect of Nrf2 and β-catenin in cancer initiation and progression. Most Recently, Mattu et al. showed that a gain of function mutation of the gene for Nrf2 is not mandatory for the development of chemically induced HCC in mice but not in human and rat [61]. Nevertheless, Zavattari et al. (2015) showed that Nrf2 is activated early in HCC formation and is probably essential for clonal expansion of preneoplastic hepatocytes. The authors assume that Nrf2 induces a pro-tumorigenic window that facilitates β-catenin-driven cancer development [23], whereas Comerford et al. (2016) described the opposite. Persistent β-catenin activity in hepatoblastoma generated a preneoplastic window by activating Nrf2, leading to hepatoblastoma formation [13]. Our results confirm and indeed resolve these two contradictory studies. The formation of HLN obviously requires both Nrf2 and β-catenin.

Nrf2 is known to have anti-inflammatory properties and to protect against toxins and oxidants. Recent publications suggest a third mode of action for Nrf2. Besides combating stressors and inflammation, Nrf2 appears to play a key role in initiating tissue regeneration by activating progenitor/stem cells [2,6,7,62,63]. Meanwhile, researchers have described a longer series of Nrf2 target genes involved in stem-cell activation in tissues. These include Notch [64], MyoD [6], VEGF [4] or AREG [3], as well as the Nrf2-driven expression of β-catenin described here. Nevertheless, this regenerative function of Nrf2 carries risks. Gain-of-function mutations in the genes that encodes for the Nrf2 pathway could lead to a permanent activation of these pro-proliferative target genes, such those for β-catenin; resulting in carcinogenesis. In addition, chronic liver diseases such as NASH or viral hepatitis lead to persistent Nrf2 activation [65]. Nrf2 in such cases improves the patients’ survival but at the same time generates a pro-tumorigenic environment that facilitates tumor development. Our results represent a turning point in our understanding of the role of Nrf2 in regeneration and liver carcinogenesis.

## 4 Materials and methods

### 4.1 Mouse strains

Conditional AlbCre:Keap1^loxP/loxP^ knockout mice (referred to as Keap1:Alb-Cre) were generated by crossing Keap1^loxP/loxP^ mice with ones expressing Cre recombinase under the control of the albumin (Alb) promoter [66]. In these mice, Keap1 deletion is limited to hepatocytes, oval cells and biliary epithelial cells [46]. To visualize Cre expression, we crossbred the Keap1:Alb-Cre mice with B6.Cg-Gt(ROSA)26Sortm9(CAG-tdTomato)Hze/J: (JAX labs stock No: 007909, also called Ai9 or Ai9(RCL-tdT)) mice to obtain AlbCre:Keap1:Ai9 mice. Ai9 mice harbor a targeted mutation of the Gt(ROSA)26Sor locus with a loxP-flanked STOP cassette preventing transcription of a CAG promoter-driven red fluorescent protein variant (tdTomato). Nrf2^−/−^ mice were generated as previously described [67]. The Keap1/Nrf2 double-knockout (designated as Keap1:Alb-Cre:Nrf2^−/−^) strain was generated by crossbreeding Keap1:Alb-Cre with Nrf2^−/−^ mice. The Keap1:β-catenin:Alb-Cre mice were established by crossing Keap1:Alb-Cre mice with Ctnnb1^tm2Kem^ mice that were kindly provided by Prof. Dr. Birchmeier of the Max Delbrück Center for Molecular Medicine in Berlin, Germany [68]. WT littermates of Nrf2^−/−^ mice were used as WT control group. All mice used in this study were generated on a C57BL/6J background.

All mice were maintained in our animal facilities under specific pathogen-free conditions. Mice were housed in 12h light/dark cycles with free access to food and water.

All experiments were conducted in accordance with German legislation governing animal studies. The Principles of Laboratory Animal Care were followed and experiments were approved by the ‘‘Landesamt fuer Natur, Umwelt und Verbraucherschutz Nordrhein-Westfalen’’.

### 4.2 Sample isolation

Liver samples were snap-frozen in liquid nitrogen and stored at −80°C for isolation of RNA and proteins. For histology, histochemical and immunofluorescent analyses, the samples were either fixed in 4% formaldehyde and embedded in paraffin or unfixed embedded in Tissue-Tek® O.C.T. Compound (Cat.#: 4583, Sakura Finetek, Staufen, Germany) on dry ice.

### 4.3 Xenogen IVIS imaging

The mice were sacrificed and their abdomens opened. Fluorescence measurement and imaging was performed on a Xenogen IVIS Lumina 100 imaging device with a DsRed filter set (Exc/Em = 551nm/583 nm) and using the following measurement parameters: FOV = 12.5 cm; binning = 2; exposure time = 1 s.

### 4.4 Histology, immunohistochemistry and immunofluorescence

Liver samples were embedded in paraffin, sectioned (5 μm) and stained with haematoxylin and eosin (H&E) for histological evaluation.

For immunohistochemical analysis the paraffin-embedded samples were sectioned (5 μm), deparaffinized in xylene, and rehydrated in dilutions of ethanol and deionized water. Antigens were retrieved by heating sections in antigen retrieval solution (15 mL of 1 M sodium citrate and 15 mL of 1 M citric acid in deionized water, pH 6.0) in a microwave for 20 min. Slides were washed in phosphate buffered saline (PBS) and 0.05% Tween for 5 min. Samples were treated with 5% bovine serum albumin (BSA) in PBS for 30 min to block non-specific binding.

For Immunofluorescence staining cryopreserved liver tissue sections (5 μm) were fixed with 4% PFA (Roth, Karlsruhe, Germany) or ice-cold acetone, washed in PBS containing 0.02% sodium-azid (Roth, Karlsruhe, Germany) and blocked using 2% BSA in PBS-azid or a 1:5 dilution of blocking reagent (Promega, Mannheim) before antibody incubation. Targets were detected via Alexa-488 or Alexa-555labeled secondary antibodies (Molecular Probes/Invitrogen, Karlsruhe).

To characterize lesions in the liver of aged Keap1:Alb-Cre mice (Fig. 2: hepatoblastoma-like neoplasia, HLNs), we performed 21 IHC or IF staining. In the analyzed mice, the HLNs were too small to be found on enough consecutive liver sections to investigate all markers in one liver. However, we identified a specimen from which we were able to get enough sections to make up a total of 7 IHC stains on consecutive sections. We therefore used this specimen to stain at least one marker of each category investigated in Fig. 2. As further controls, we included WT liver samples and no primary controls (NPCs) for these staining, which are also shown in Fig. S3.

Pictures of each group were taken using a Keyence BZ-9000 microscope. Detailed information regarding antibodies is given in Table S1.

tdTomato fluorescence of cryopreserved sections (Keap1:Alb-Cre:Ai9 mice) was recorded using a LSM710 together with an Airyscan (Carl Zeiss Microscopy, Jena, Germany). Resulting images of tdTomato fluorescence were color coded for signal intensity using a custom Lookup Table (LUT: 0–1 - > RGB 0,0,50–0,0,152 [Blue]; LUT: 2–37 - > RGB 0,50,0–0,249,0 [Green]; LUT: 38–255 - > RGB 0,50,50-0,254,254 [Cyan]) for image J 1.46 (NIH, USA). Identical settings were used for all tdTomato fluorescence images taken.

### 4.5 Western blot analysis

Ice-cold RIPA buffer including a protease inhibitor cocktail (Roche, Basel, Switzerland) was added to 30 mg of frozen liver tissue. Lysis was intensified by mechanical force in a Precellys® homogenizer for 20 s at 5000 rpm to obtain total protein homogenates. Protein concentration was determined using a routine bicinchoninic acid (BCA) protein assay kit as recommended by the manufacturer (Thermo Fisher Scientific Inc., Rockford, IL, USA). An amount of 25 μg of protein was heat-denaturized in double-strength sodium dodecyl sulphate sample buffer containing dithiothreitol before SDS-PAGE separation using 10% polyacrylamide gels. Subsequently, the separated proteins were transferred on PVDF membranes by semi-dry electro-blotting (Trans-Blot® Turbo™, Bio-Rad, Hercules, CA, USA). After blocking in 5% BSA TBS-T for 1 h at RT, the membranes were incubated with primary antibody overnight at 4 °C and subsequently with secondary antibody solution for 1 h at RT under continuous gentle shaking. Immunoreactivity was visualized using the chemiluminescence reagent Immobilon™ Western (Millipore, Darmstadt, Germany) as recommended by the manufacturer. Luminescence signals were detected on photosensitive Hyperfilm™ ECL (GE Healthcare Life Science, Chalfont St. Giles, United Kingdom) in a light-excluded dark room. The antibody complexes were removed with Roti® Free Stripping Buffer (Carl Roth, Karlsruhe, Germany) for 15 min at 60 °C, the membrane was blocked again, and the primary antibodies used for reference (GAPDH or actin) were applied. The secondary antibodies used in this work were either HRP-conjugated anti-mouse immunoglobulin G (1:20,000; cat.#: A2304; Sigma Aldrich, Frankfurt, Germany) or HRP-conjugated anti-rabbit immunoglobulin G (1:20,000; cat.#7074; CST, Danvers, MA, USA). For densitometry, the density of the Western blot bands was captured using a flatbed scanner; intensities were determined applying the Quantity One software version 4.6.9 (Biorad; Hercules, CA, USA). The ratios between band intensities of the target protein and the corresponding band of GAPDH or actin were normalized to the untreated control groups and represented graphically. All applied antibodies are listed in Table S1.

### 4.6 RNA isolation, cDNA synthesis and qPCR

The RT-(q)PCR studies were conducted in compliance with the MIQE guidelines [69]. Total RNA was isolated from 30 mg of liver tissue or from cultured cells using the NucleoSpin® RNA kit as recommended by the manufacturer (Macherey Nagel, Düren, Germany). Nucleic acid quantity and purity were determined spectrophotometrically (A260/A280 & A260/A230) using the NanoDrop®ND-1000 device (Thermo Scientific, Waltham, MA, USA). RNA integrity was ensured by MOPS buffered denaturizing RNA gel electrophoresis (28S/18S rRNA ratio). Reverse transcription was performed according to the supplier's instructions using Maxima Reverse Transcriptase (Thermo Fisher Scientific, Waltham, MA USA) with mixed priming (oligo-(dT)18:random hexamer, 3:1, v/v) and 2 μg total RNA per reaction. Real-time PCR was conducted on an ABI StepOne Plus system using PowerSYBR® Green PCR Master Mix (Thermo Fisher Scientific, Waltham, MA USA). Both, qPCR as well as conventional absence/presence PCR, were performed with primer-specific pre-evaluated annealing temperatures and a standard protocol (cycles: Dissociation-Annealing-Elongation). Primer specificity was determined by both melt-curve analysis and TAE-buffered DNA agarose gel electrophoresis in qPCR and by TAE-buffered DNA agarose gel electrophoresis of the PCR product in conventional absence/presence PCR experiments.

For the quantification of qPCR data, the amplification efficiency was calculated with LinRegPCR 2016.0 software (Heart Failure Research Center, Amsterdam, The Netherlands) as described by Ramakers et al. [70]. Prior to the actual study, a reference gene evaluation including ten representative samples and 10 potential reference targets was conducted with geNorm calculations (part of the Biogazelle qbase+ software) to establish a sufficient reference gene index for data normalization. In short, this evaluation indicated that a combination of succinate dehydrogenase complex subunit A (SDHA) with glyceraldehyde 3-phosphate dehydrogenase (GAPDH) would be the most suitable normalization strategy for this experimental setup. Inter-run variation was corrected by the use of inter-run calibrators in each PCR run. The relative fold-change of gene expression was calculated with qbase+ according to the recommended efficiency-corrected ΔΔCq method, as reported by Pfaffl (2001) [71]. All applied qPCR primers are listed in Table S2 in the supplements.

### 4.7 Cell culture

Hepatocytes were obtained from ten or ninety week old Keap1:Alb-Cre, Keap1:Ai9:Alb-Cre, Nrf2^−/−^ and WT mice. Before laparotomy, the mice are treated with a mixture of 10% ketamine solution and 2% Rompun solution (Xylazin) (final concentration: 100 mg/kg ketamine and 10 mg/kg Rompun). Thereafter, a 22G BD Insyte™ cannula was inserted into the vena cava inferior and fixed. The portal vein was cut and the liver was perfused with Earle's balanced salt solution without Ca2^+^-and Mg^2+^ [EBSS Ca^2+^- Mg^2+^-] (Gibco) until the liver was completely bloodless. The buffer was then changed to EBSS [Ca^2+^ Mg^2+^] and HEPES (10 mM) pH 7.5. In the final step, the liver was perfused with 100 mL EBSS containing Ca^2+^, Mg^2+^,10 mM HEPES, 0.3 mg/mL collagenase and 0.04 mg/mL trypsin inhibitor. The liver was harvested and the cells were dispersed in William's Eagle medium. The suspension was filtered through a 70 μm cell strainer and centrifuged at 50×*g* for 5 min. Cells were washed twice in cold William's Eagle medium, checked for viability via trypan blue staining and finally resuspended to a concentration of 1 × 10^6^ cells per 100 μL and plated on 10 cm dishes.

In contrast to primary WT hepatocytes, cells with Keap1 deficiency were subculturable, so that we were able to establish immortal cell lines. To clearly distinguish these newly generated cell lines, they were named as follows:

• cells from 10-week-old Keap1:Alb-Cre mice that were used without subculturing were named “**pHep-dKeap1**” (primary hepatocytes deficient for Keap1)
• cells from 10-week-old Keap1:Alb-Cre mice that were subcultured (up to 100 days) were named “**iHep-dKeap1**” (immortal hepatocytes deficient for Keap1)
• cells from 90-week-old Keap1:Ai9:Alb-Cre mice that were subcultured (up to 240 days) were named “**HLN119-Ai9**” and “**HLN133-Ai9**” (hepatoblastoma-like neoplasia cells from mouse 119/133 with tomato)

Mouse non-transformed hepatocyte AML12 cell line and the hepatoma cell line Hepa1-6 was purchased (American Type Culture Collection, Manassas, VA) and cultured in DMEM/F-12 medium or DMEM, respectively, supplemented with 10% FCS and 1% penicillin/streptomycin, at 37 °C with 5% CO_2_ used to derive stable humidified incubators.

### 4.8 Lentiviral short hairpin RNA (shRNA) delivery

Lentiviral gene delivery was used to transduce the HLN133-Ai9 cells with a pLKO.1 shRNA construct (MISSION™ shRNA assortment, Sigma-Aldrich, Germany) directed against Nrf2 (TRC clone id: TRCN0000054658) or β-catenin respectively (TRC clone id: TRCN0000012690). In order to exclude variations due to the vector backbone and to prove that observed effects are exclusively dependent on the specific shRNA, we also transduced hepatocytes with a shNonTarget (shNT) control construct. To verify the knockdown efficiency of the shRNA constructs, we performed qPCR and Western blot analyses.

## 5 Dual luciferase reporter gene assay

### 5.1 Plasmid construction

The pGL3-Ctnnb1-784- and pGL3-Ctnnb1-784-ΔARE reporter plasmid consisted of 784 bases directly upstream from the murine gene for β-catenin cloned into the pGL3 Luciferase Reporter Vector (Promega) Mutagenesis of the ARE-Sequence was performed by replacing the TGA binding site into CTG to produce a dysfunctional ARE sequence. Additionally, the human proximal SOX9 promoter, 1034/+67 relative to the transcriptional start site were cloned into a pGL3 reporter plasmid.

### 5.2 Transient transfection

Transfection approaches were performed with the jetPRIME™ transfection kit. 3 × 10^5^ iHep-dKeap1 cells were plated on 6 well plates. The next day, cells were co-transfected with 1.66 μg vector plasmid DNA of the β-catenin promotor construct with or without a functional ARE sequence (Ctnnb1 874; Ctnnb1 874 –ΔARE) or Sox9 promoter sequence and 0.33 μg of the vector system expressing Renilla reniformis luciferase for data normalization. The transfection medium was replaced with fresh William's E medium after 6 h Cells were used for dual luciferase reporter gene assays 48 h after transfection.

iHep-dKeap1 cells, which were transfected with reporter gene constructs, were stimulated with 2.5 μM sulforaphane (SFN) or methysticin (Meth) to potentiate Nrf2 signaling. Cells were transferred, at a concentration of 3 × 10^4^ cells/well, onto a 96-well plate and incubated for 24 h in William's E medium with 5% FCS. Then, Hepatocytes were stimulated with SFN for 6 h or with Meth as specified. Unstimulated controls were treated with the same amount of DMSO, which served as vehicle control. After the stimulation, cells were washed with sterile PBS before lysis was achieved by adding 100 μL of passive lysis buffer (Promega, Madison, WI, USA) to each well of the 96-well plate; 50 μL of lysate was transferred onto a white 96-well microtiter plate for luminescence measurement in a GLOMAX Navigator luminometer (Promega), as described previously [5]. Firefly luciferase activity was normalized to that of Renilla luciferase.

### 5.3 Chromatin immunoprecipitation assay (ChIP)

ChIP assay was performed to test whether Nrf2 binds directly to the ARE sequence in the β-catenin promoter. 4*10^6^ primary hepatocytes isolated from 10 week old Keap1:Alb-Cre mice (pHep-Keap1-KO) were cultivated on 10 cm dishes. After 48h, cells were fixed for 15 min by adding 1% formaldehyde to the medium. Afterwards, the reaction was stopped by admix 125 mM glycine for 5 min; the medium was discarded, and cells were harvested using a cell scraper. Cell pellet was washed two times with ice-cold PBS containing protease inhibitors and treated with membrane extraction buffer. To cut the DNA into 400–500 bp fragments, micrococcal nuclease (MNase) was added and incubated for 15 min at 37 °C. To separate nucleic DNA fragments, the nuclear membrane was broken by incubating the nuclei in nucleus extraction buffer (NEB) on ice on a shaker for 30 min. Of this,15 μL were saved as input DNA and stored at −20 °C until needed. To the remaining lysate, 5 μg of Nrf2 antibody (Santa Cruz H300x) were added, and it was incubated overnight on a head-over-heel rotator at 4 °C. The next day, 20 μL of magnetic beads with antibody binding affinity (Pierce™ Magnetic ChIP Kit, ThermoFisher Scientific) were added and incubated for 2 h at 4 °C. Using a magnetic bead separation rack, the specific Nrf2/nucleic acid complexes were isolated and transferred to the elution buffer. After 30 min incubation at 65 °C, samples were treated with protein kinase K to release the protein/DNA complex. The pure DNA was cleaned up using DNA-binding columns. 2 μL of DNA was used for PCR and subsequent agarose gel separation. For the PCR, specific primers against the ARE sequence in the Ctnnb1 promoter were used. The well-characterized ARE sequence of glutathione S-transferase (GST) promoter functioned as a positive control, whereas primer against the promoter of the actin gene without an ARE-sequence served as a negative control (Supplemental Table 3: List of primers used for ChIP-Assay).

### 5.4 Electrophoretic mobility shift assay (EMSA)

Electrophoretic mobility shift assays are a technique for evaluating the DNA-binding properties of a protein. They operate on the principle that DNA-protein complexes migrate more slowly through a non-denaturing polyacrylamide gel than free unbound DNA does. We used the fluorescence-based Odyssey® Infrared EMSA Kit distributed by LI-COR. Four million primary hepatocytes isolated from Keap1:Alb-Cre (pHep-dKeap1) and Nrf2^−/−^ (pHep-dNrf2) mice were cultured in 10 cm dishes for 48h, and nuclear protein was isolated by using NE-PER™ Nuclear Cytoplasmic Extraction Reagent as described by the manufacturer (Pierce, ThermoFisher Scientific). IRDye-700-labeled oligonucleotides (50 bp) corresponding to the ARE sequence of the murine Ctnnb1 promoter and the competitor nucleotides were synthesized by MWG Eurofins (Supplemental Table 4: Oligonucleotides used for EMSA). 5 μg of nuclear protein was added to the working solution and incubated for 30 min with 0.1 nM labeled oligonucleotides in the presence as well as absence of the competitor (Table S5). Electrophoresis was conducted at 70 V for 40 min under non-denaturing conditions (0.5 M TBE buffer pH 8). DNA detection was conducted with the LI-COR Odyssey imaging system at a resolution of 84 μm and an intensity of 5.0 at high quality.

### 5.5 Cell proliferation assay

In order to test whether β-catenin or Nrf2 knockdowns affect the proliferation behavior of HLN133-Ai9 cells, a proliferation assay was performed with an Incucyte® ZOOM system. The Incucyte® system is a live cell imaging system consisting of a fully automated compact microscope system that fits inside a standard tissue culture incubator. The software enables its users to image living cells and acquire data on cell proliferation. The system's software thus permitted imaging of the same site in each culture plate every 2h for 72h using phase contrast imaging without cell labeling. Cell proliferation was calculated with an integrated confluence metric serving as a surrogate for the cell count. The experiment was designed to have eight technical replications for each different shRNA sample. For statistical analysis, the proliferation test was performed in three independent experiments on different days.

### 5.6 Statistics

Variance homogeneity was checked with Bartlett's test. The Shapiro-Wilk test was used to test for normal distribution. Box-Cox-Y transformation was conducted to achieve homoscedasticity if necessary. Parametric data were basically analyzed using ANOVA-based test procedures. The exact details which tests were applied in each case are shown in the corresponding figure legend. Data are shown as mean ± SEM. Statistical analyses were performed with GraphPad Prism 9.2 (GraphPad, La Jolla, USA) and JMP 10 (Böblingen, Germany).

## Author contributions

A.F. and J.S. designed and performed the experiments, analyzed the data and co-wrote the paper; N. S. carried out immunohistochemical examinations; M.W., T.N. and T.C. carried out the *in vitro* experiments; S.K. carried out the confocal laser scan analysis; E.B. and P.R. analyzed the data and contributed to the experimental design; T.L. analyzed the tumor histology and pathology; C.J.W. designed the experiments, analyzed the data and wrote the paper with contributions from T.P., A.M., C.T. and K.L.S.

## Declaration of competing interest

The authors declare no competing interests.

## Data Availability

Data will be made available on request.
