# Supplementary material for: Nrf2 induces malignant transformation of hepatic progenitor cells by inducing β-catenin expression
Source: Redox Biol. 2022 Sep 13;57:102453. doi: 10.1016/j.redox.2022.102453 (PMC9618468; doi:10.1016/j.redox.2022.102453)
Supplement: Multimedia component 1 [file mmc1.pdf]

NPCs – 10x lens

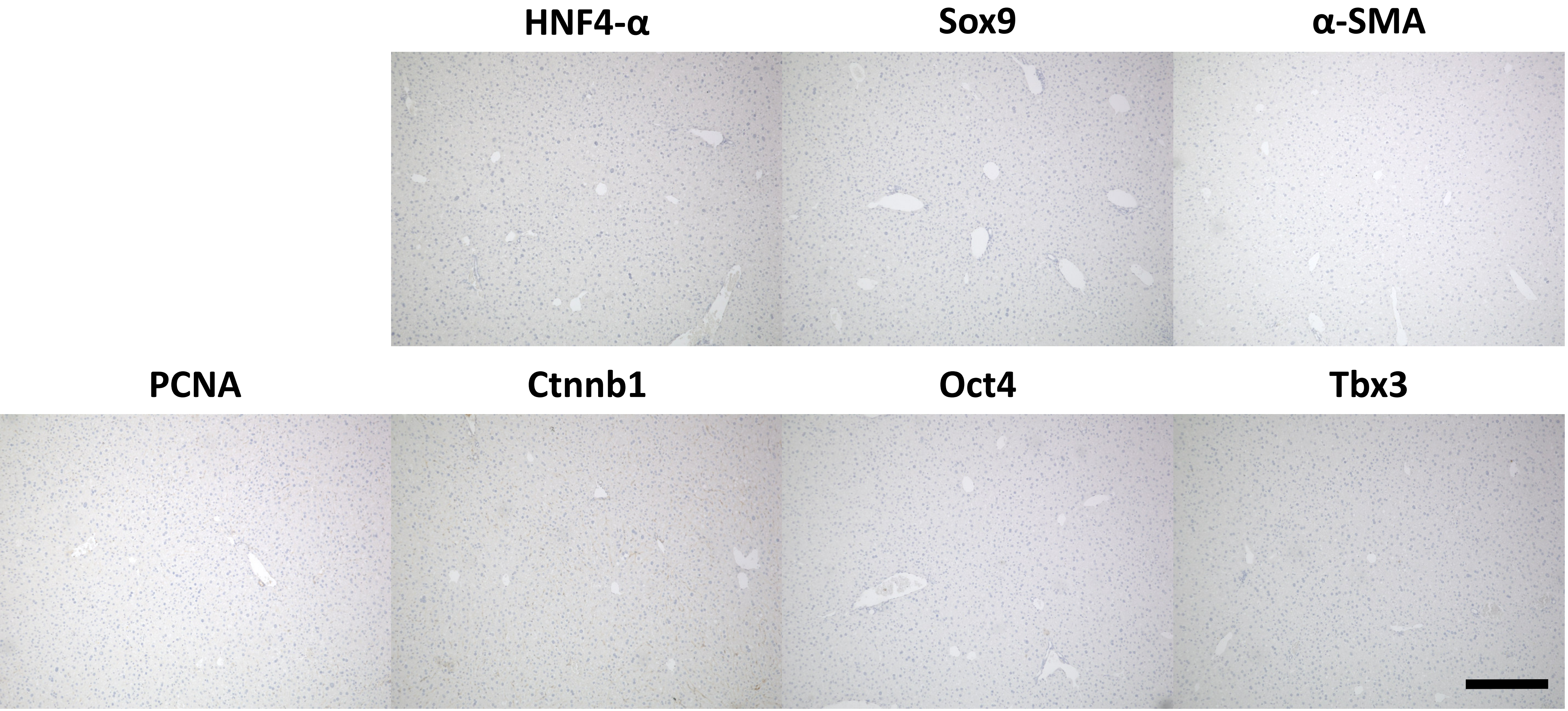

scale = 100 µm

NPCs – 20x lens

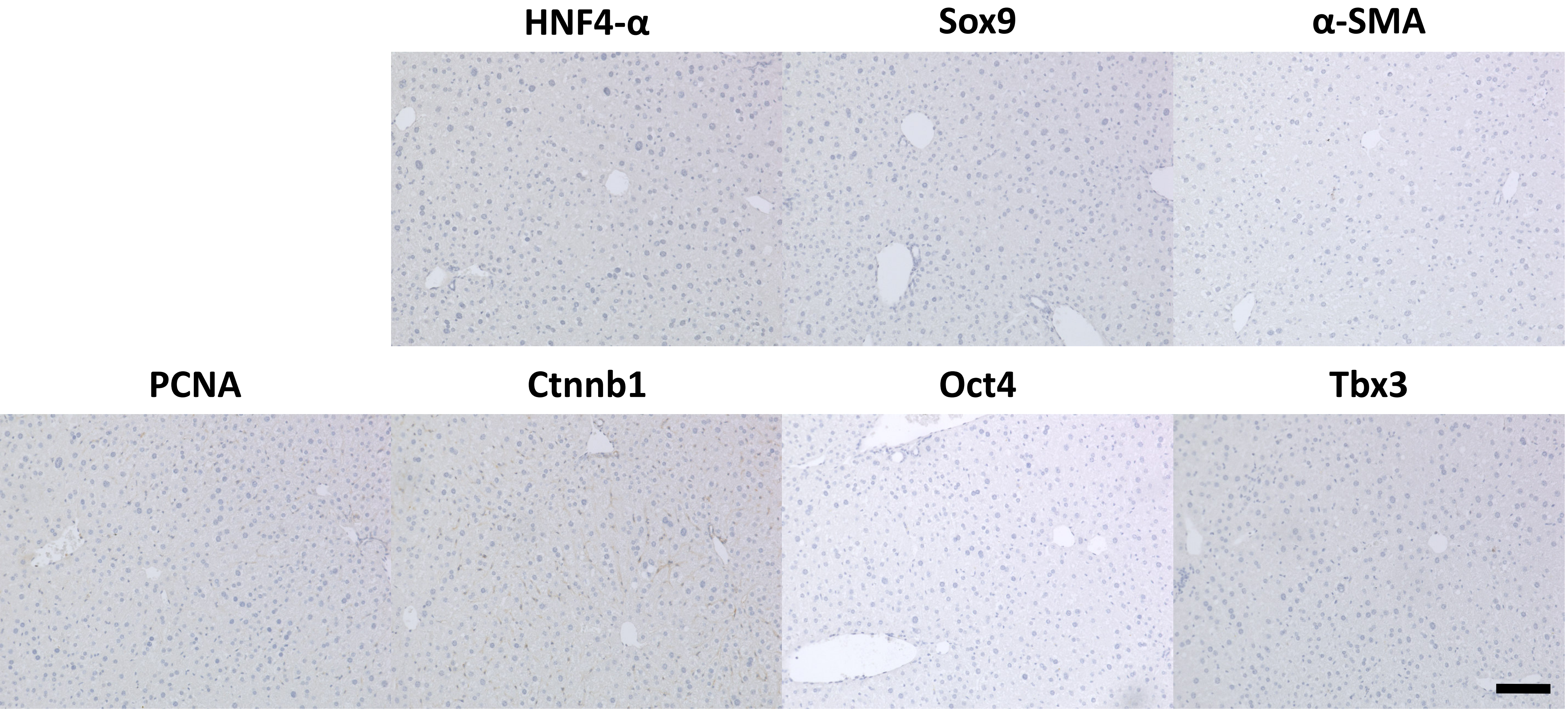

scale = 100 µm

WT – 10x lens

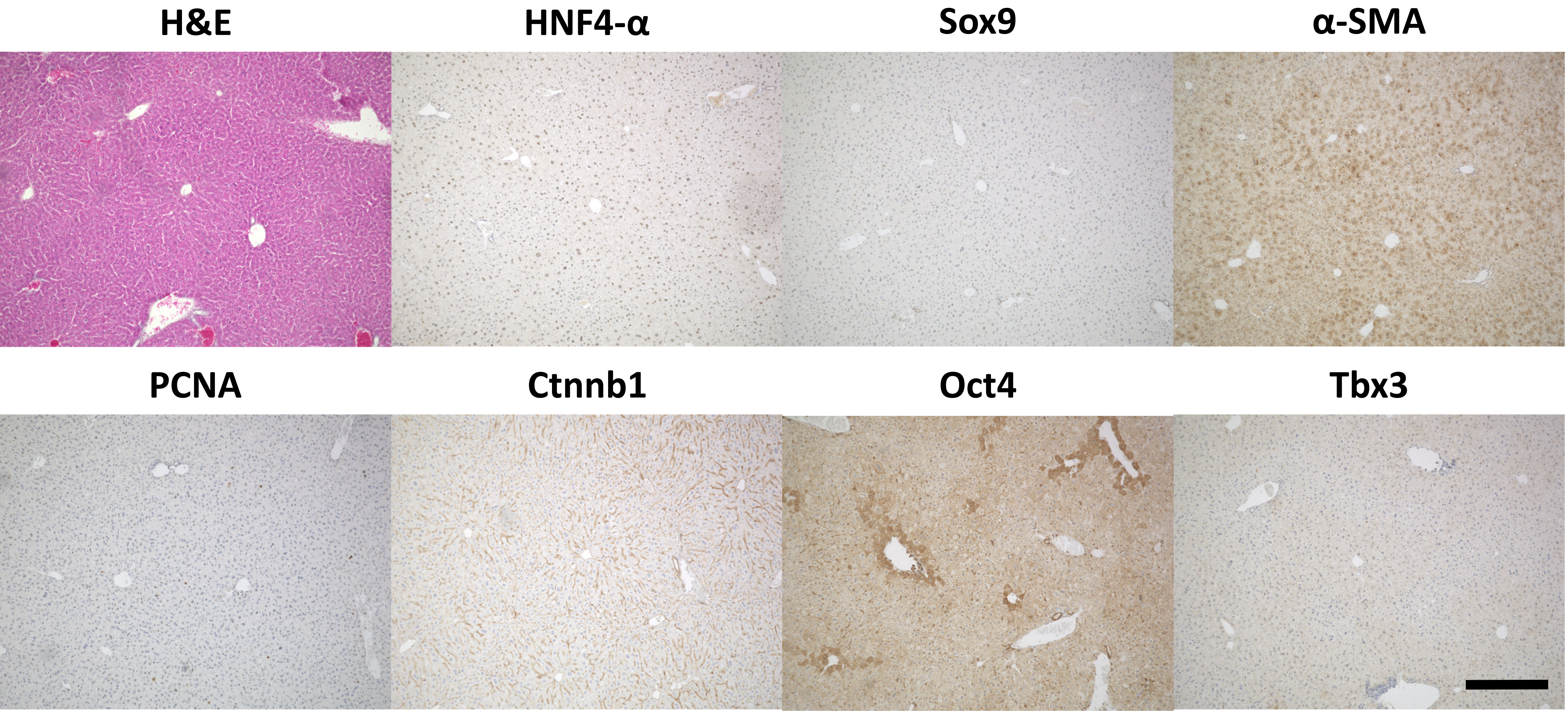

scale = 100 μm

WT – 20x lens

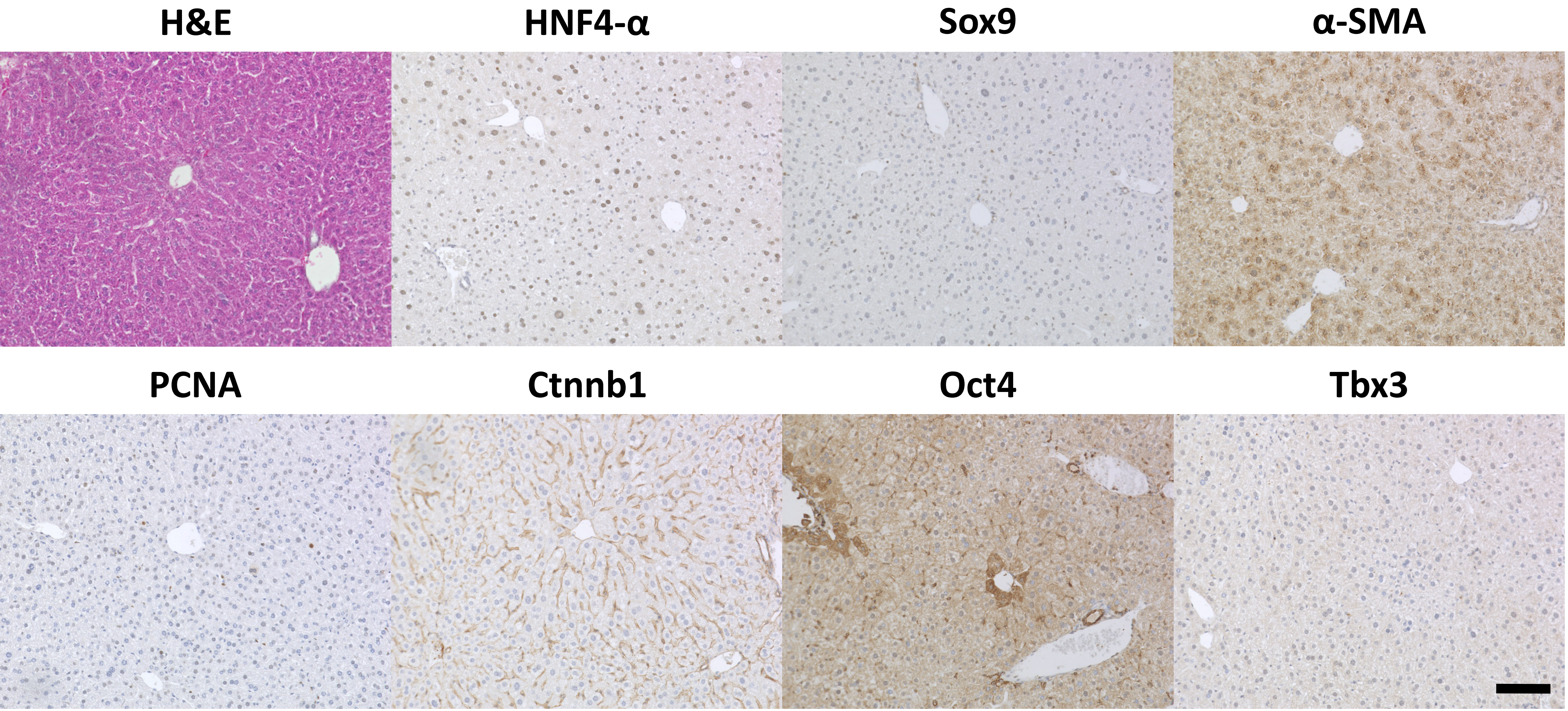

scale = 100 µm

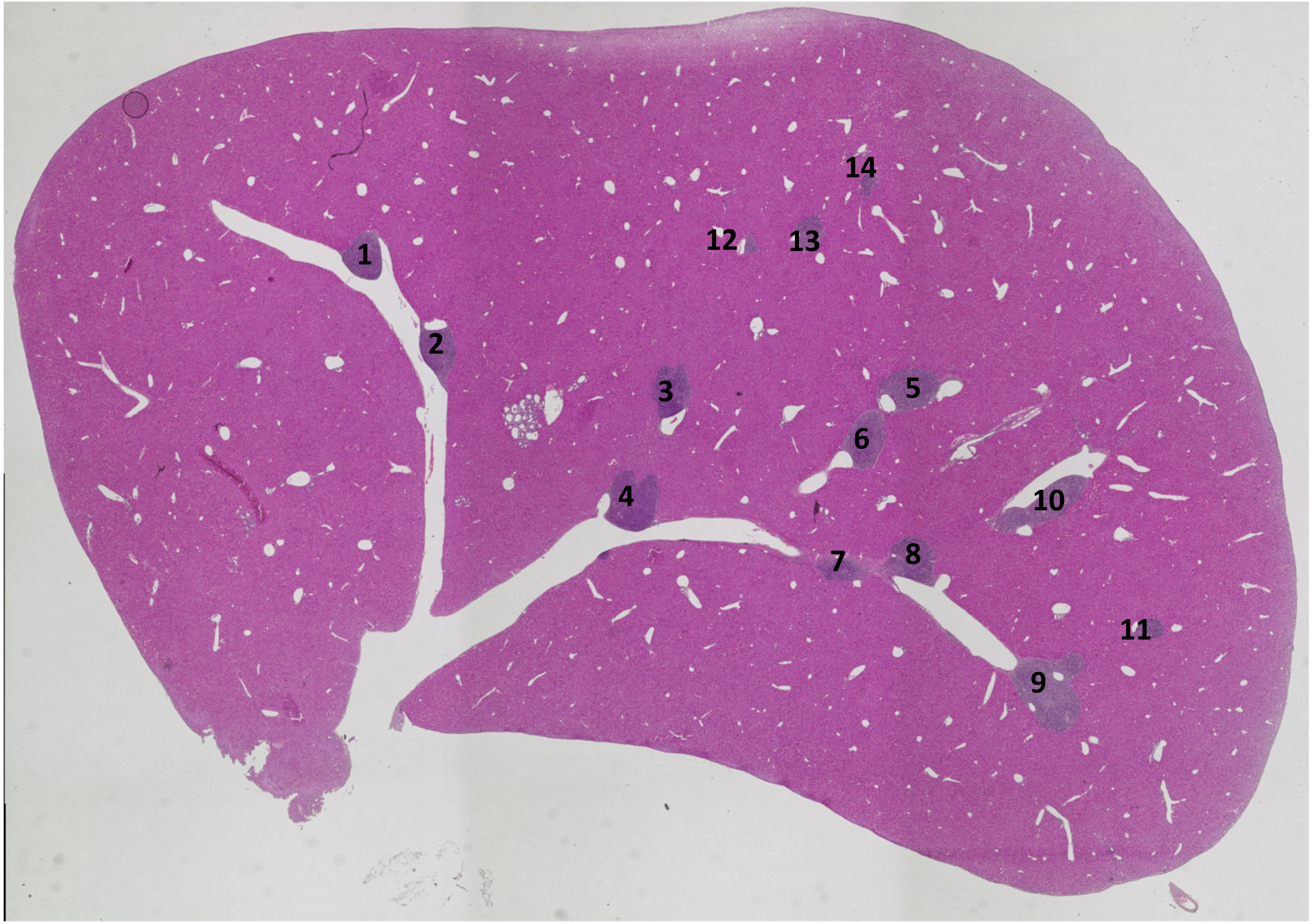
1
2
3
4
5
6
7
8
9
10
11
12
13
14

HLN 1 – 10x lens

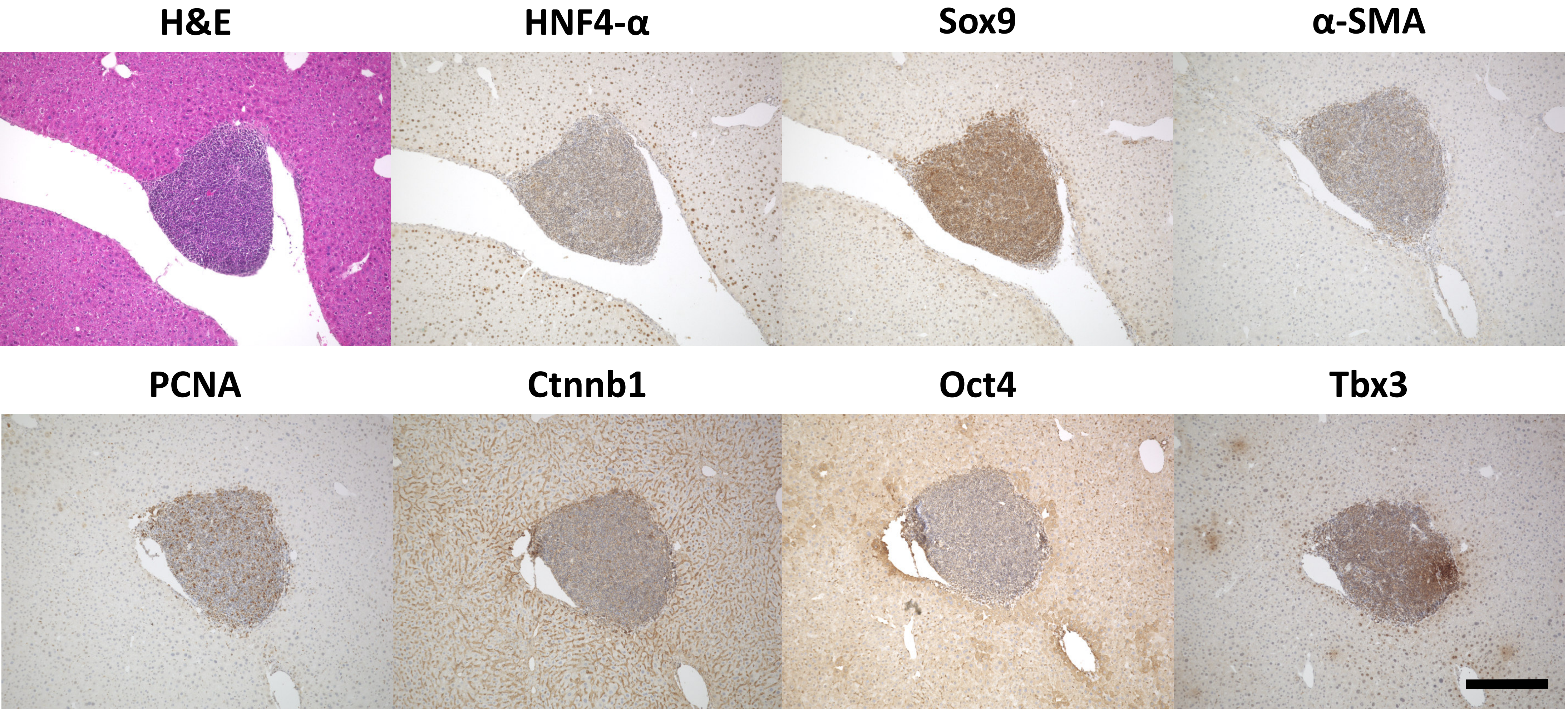

scale = 100 µm

HLN 1 – 20x lens

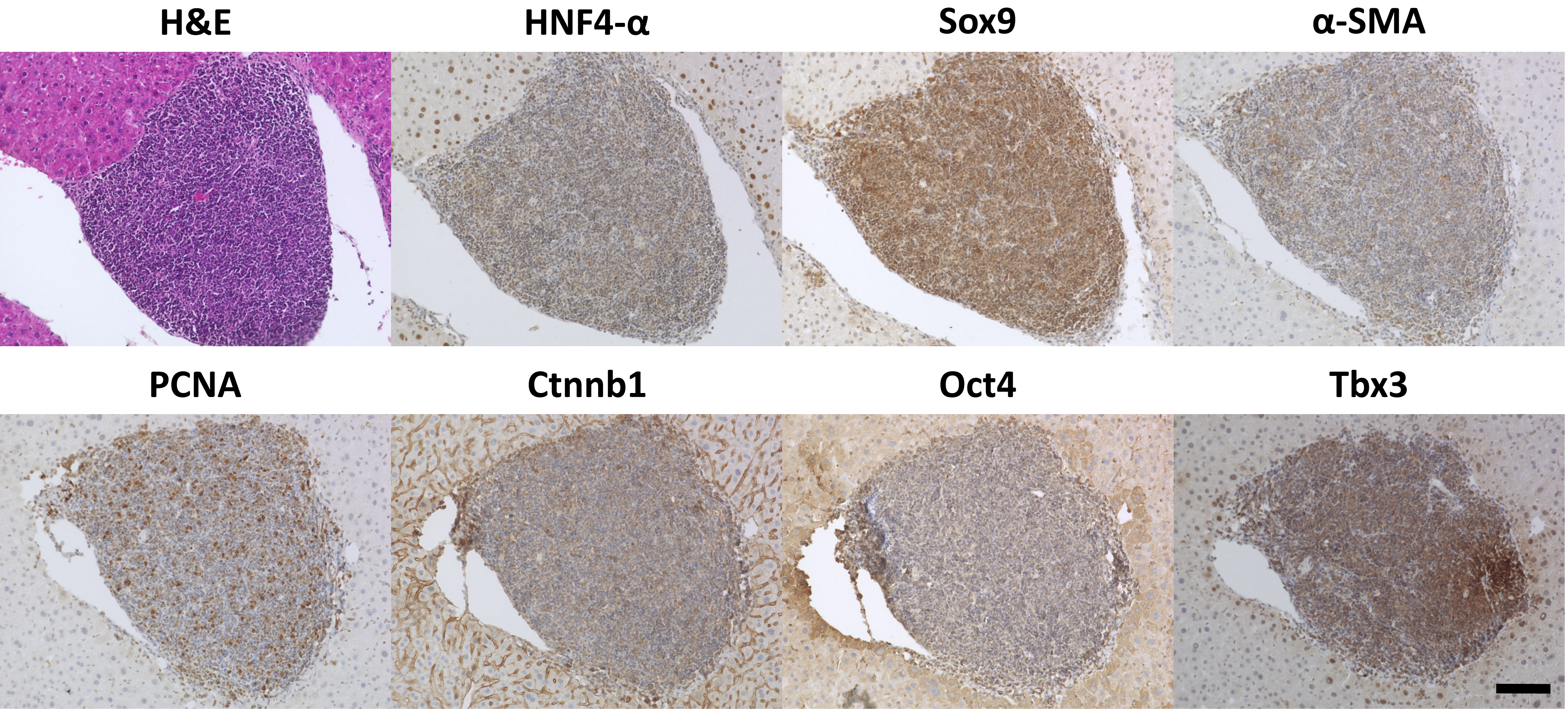

scale = 100 µm

HLN 2 – 10x lens

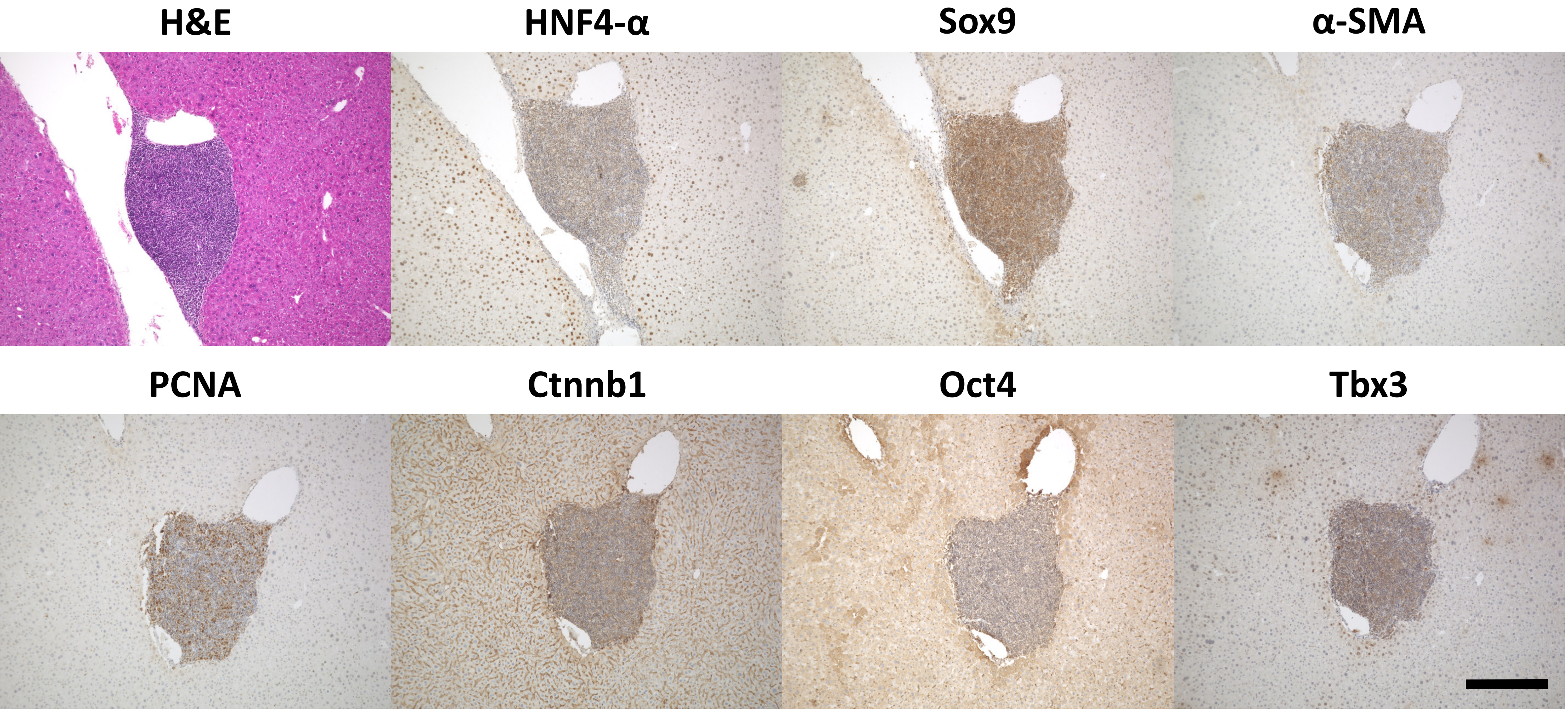

scale = 100 µm

HLN 2 – 20x lens

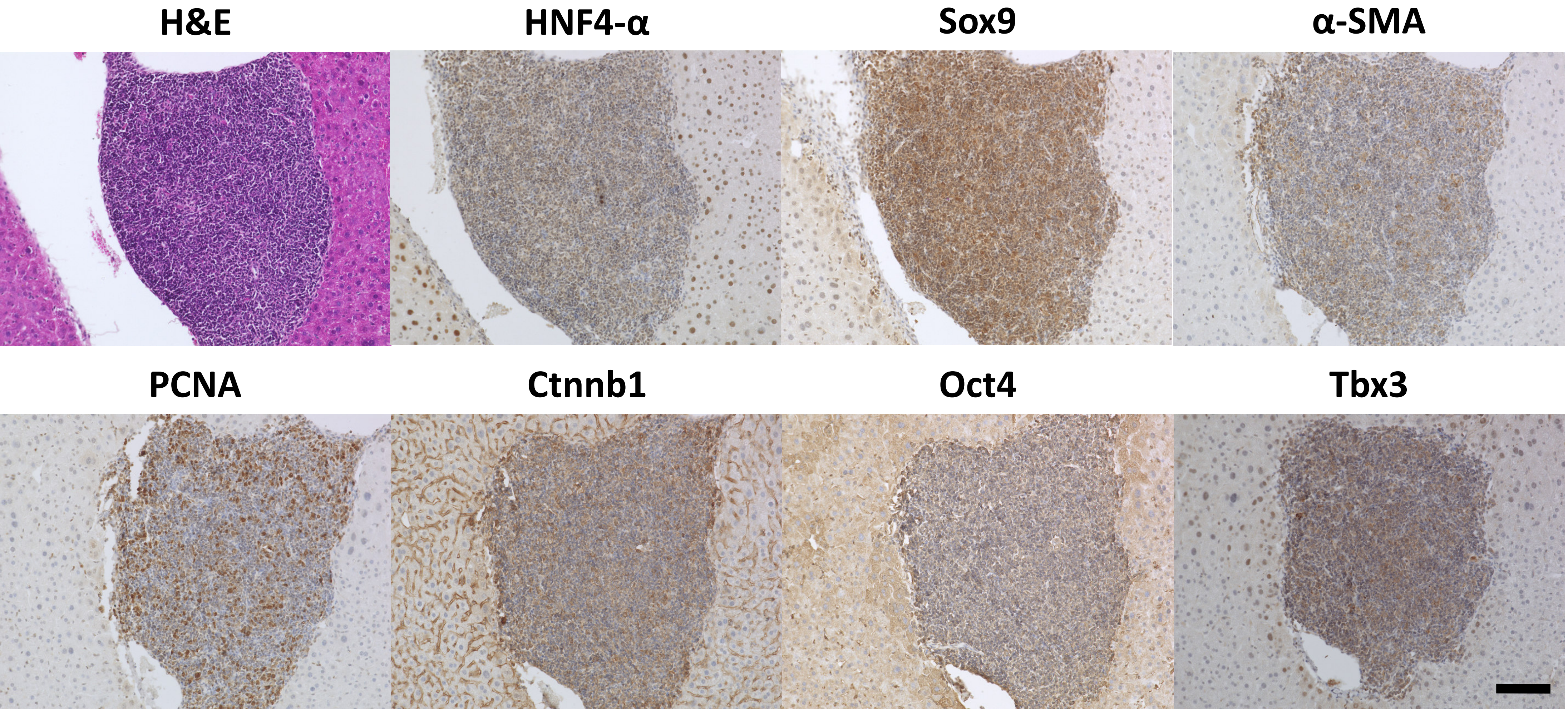

scale = 100 µm

HLN 3 – 10x lens

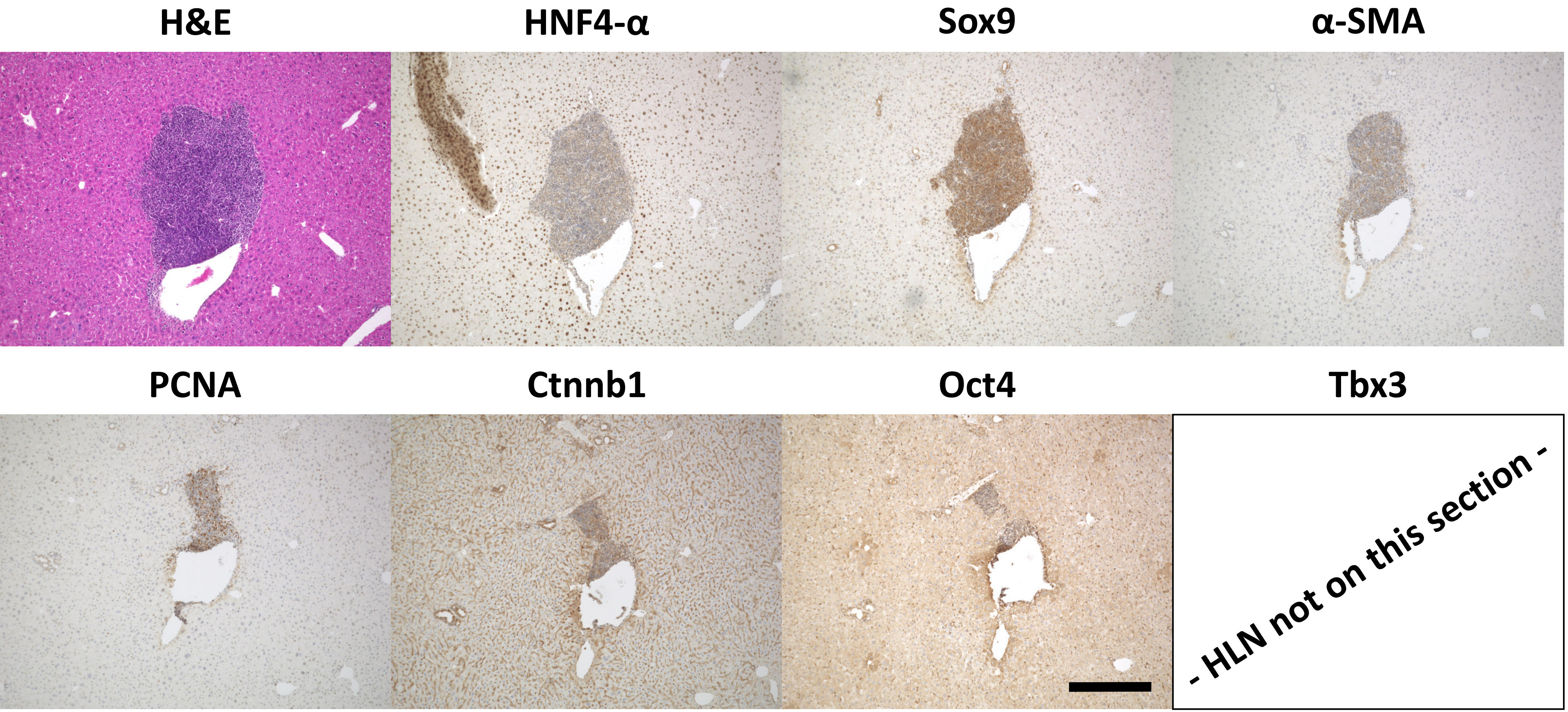

scale = 100 µm

HLN 3 – 20x lens

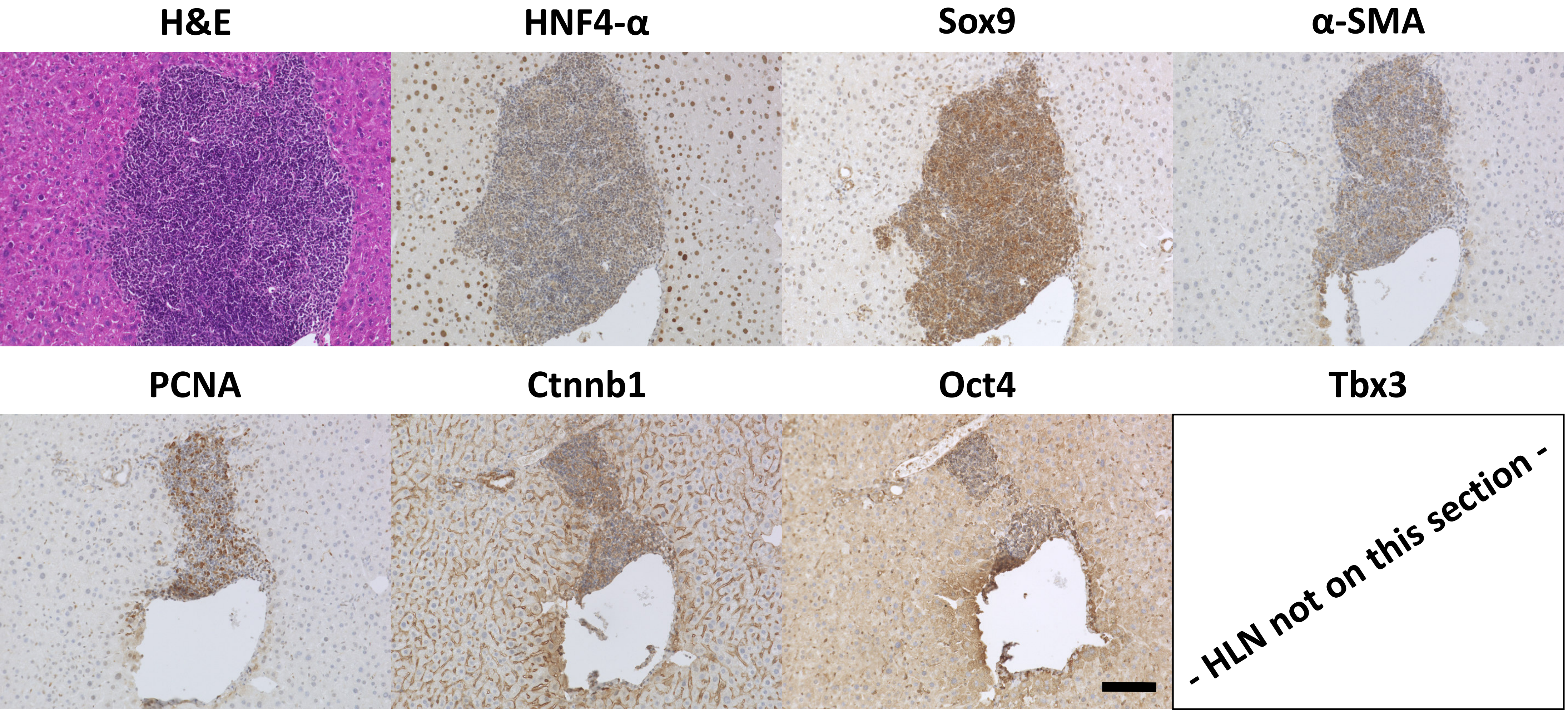

scale = 100 µm

HLN 4 – 10x lens

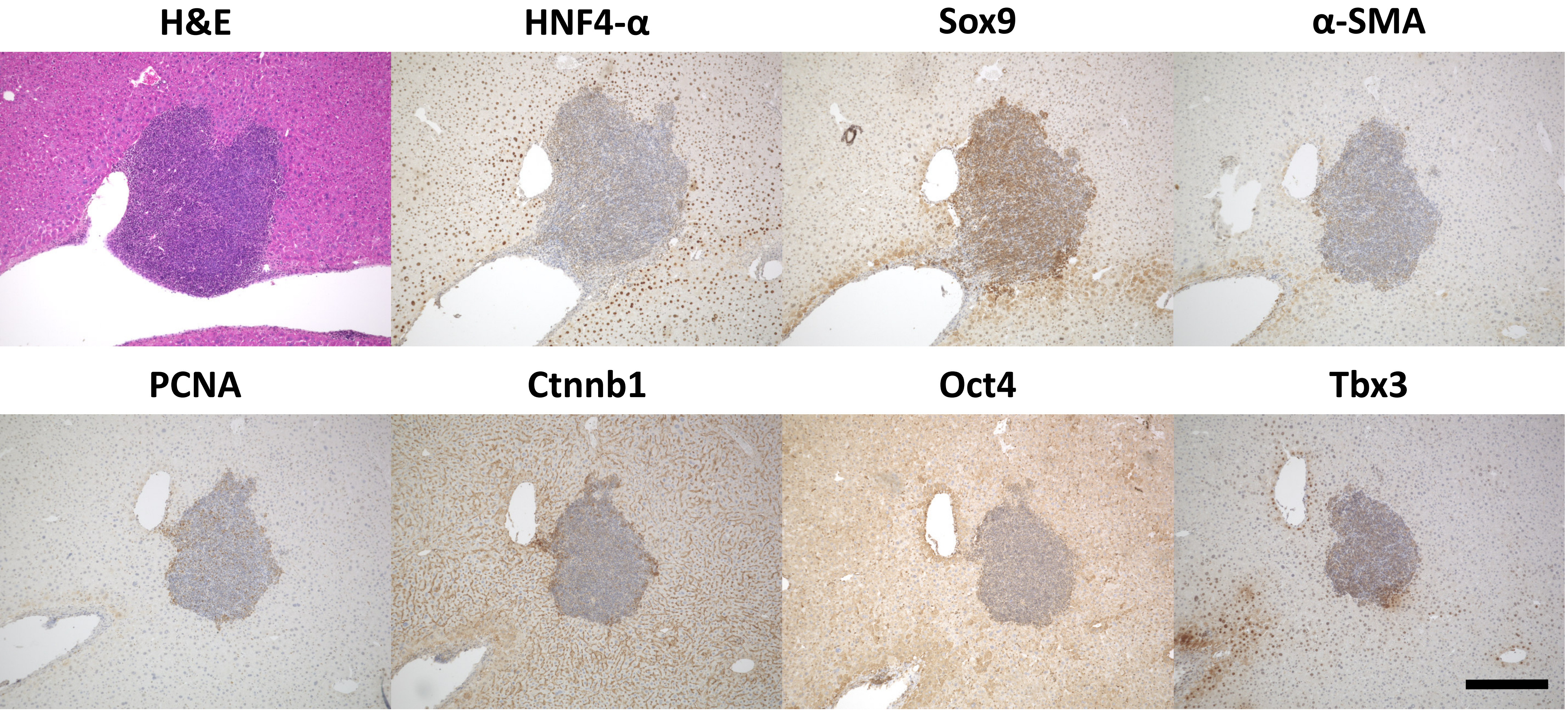

scale = 100 µm

HLN 4 – 20x lens

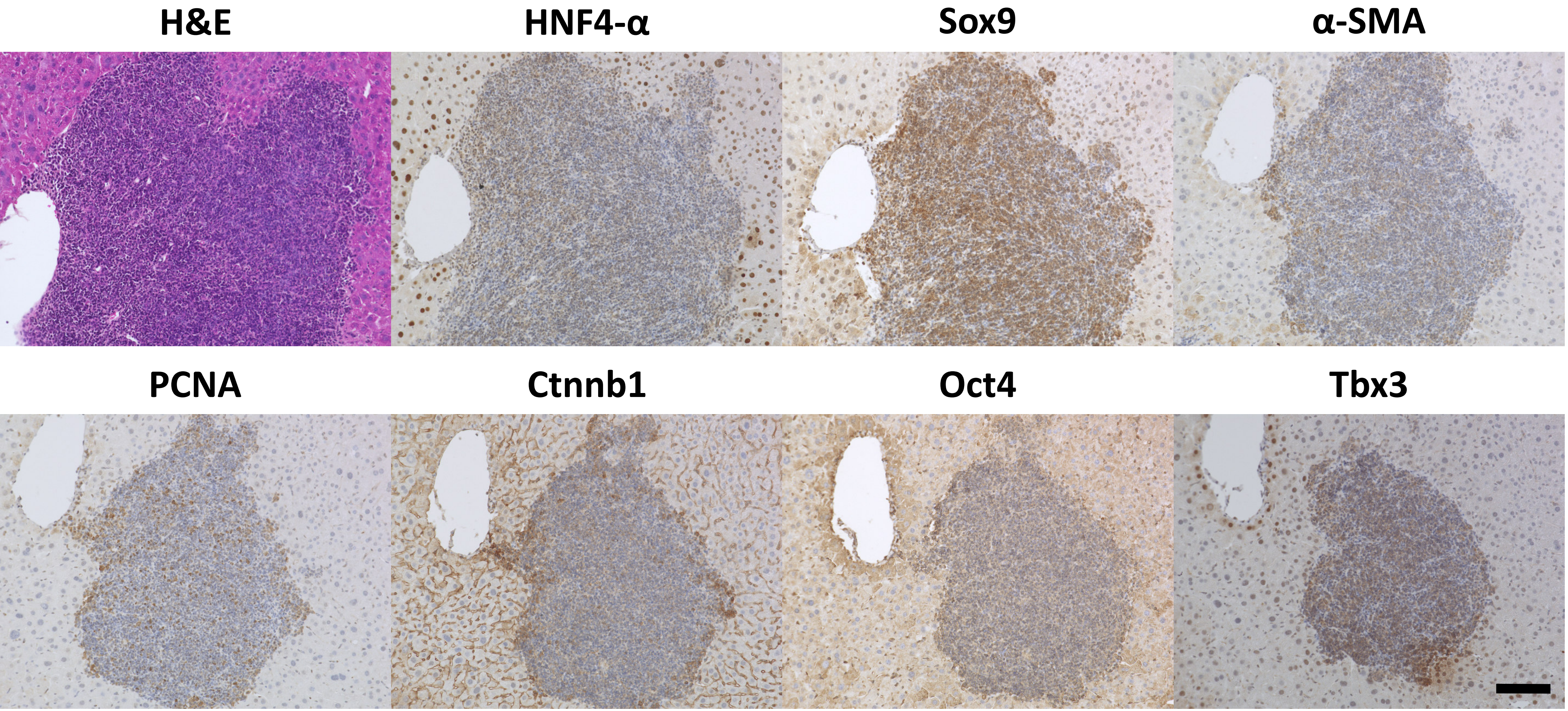

scale = 100 µm

HLN 5 – 10x lens

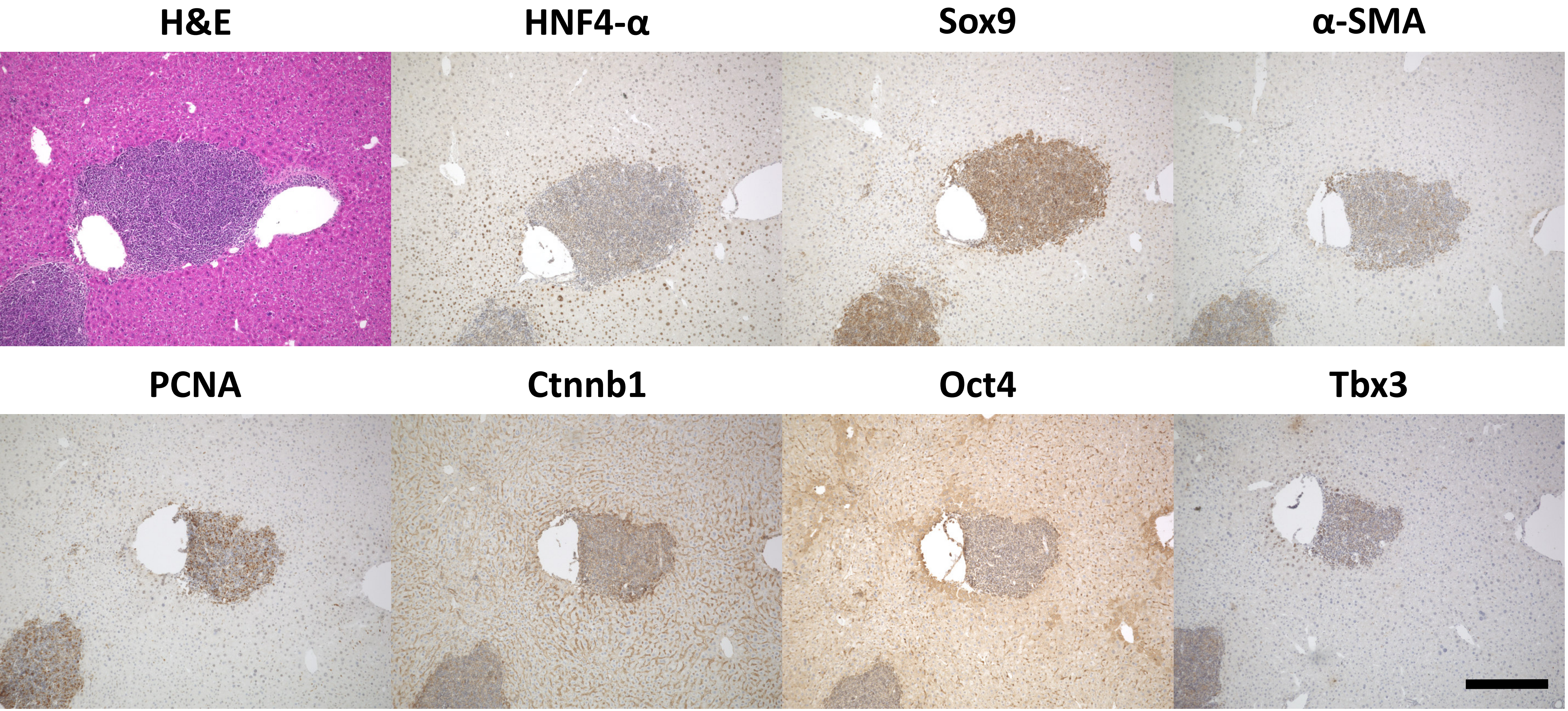

scale = 100 µm

HLN 5 – 20x lens

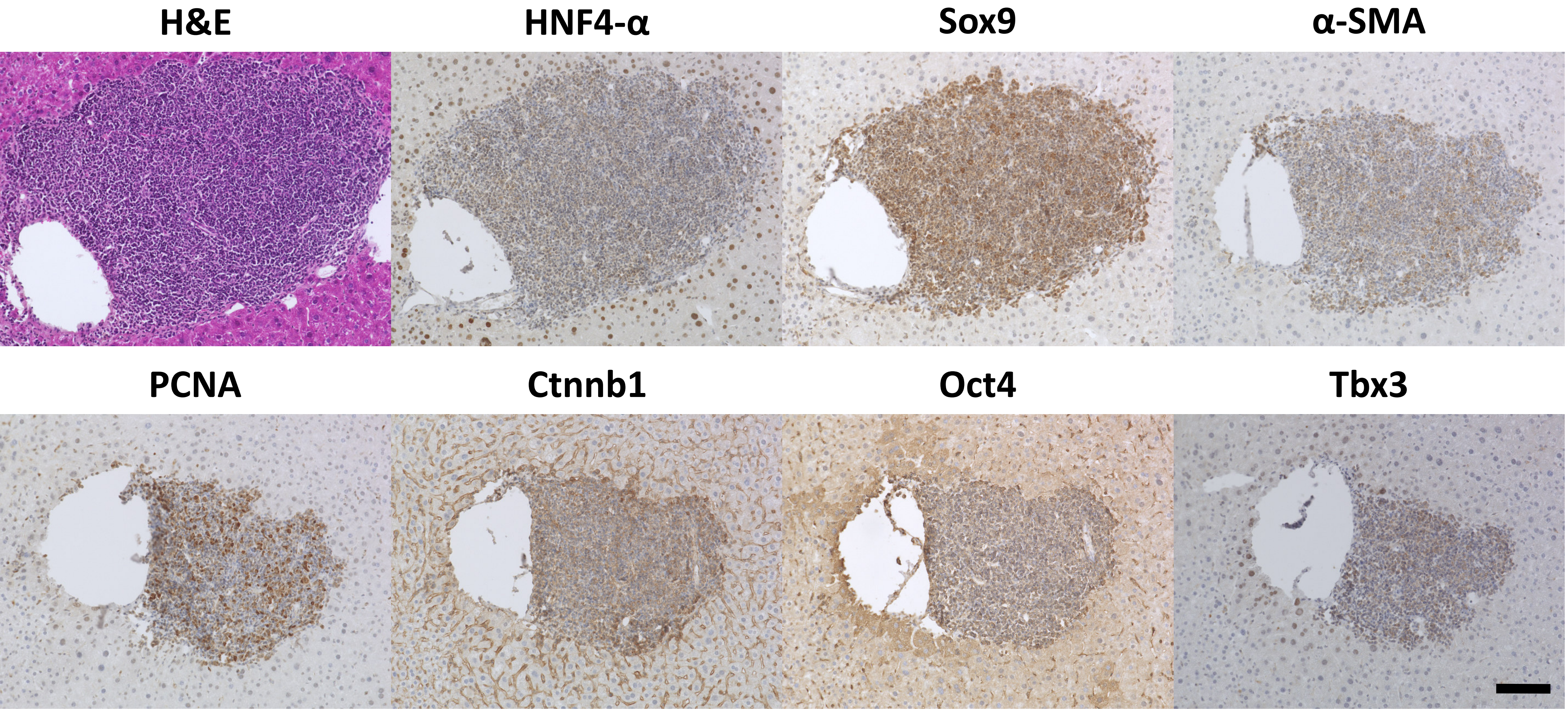

scale = 100 μm

HLN 6 – 10x lens

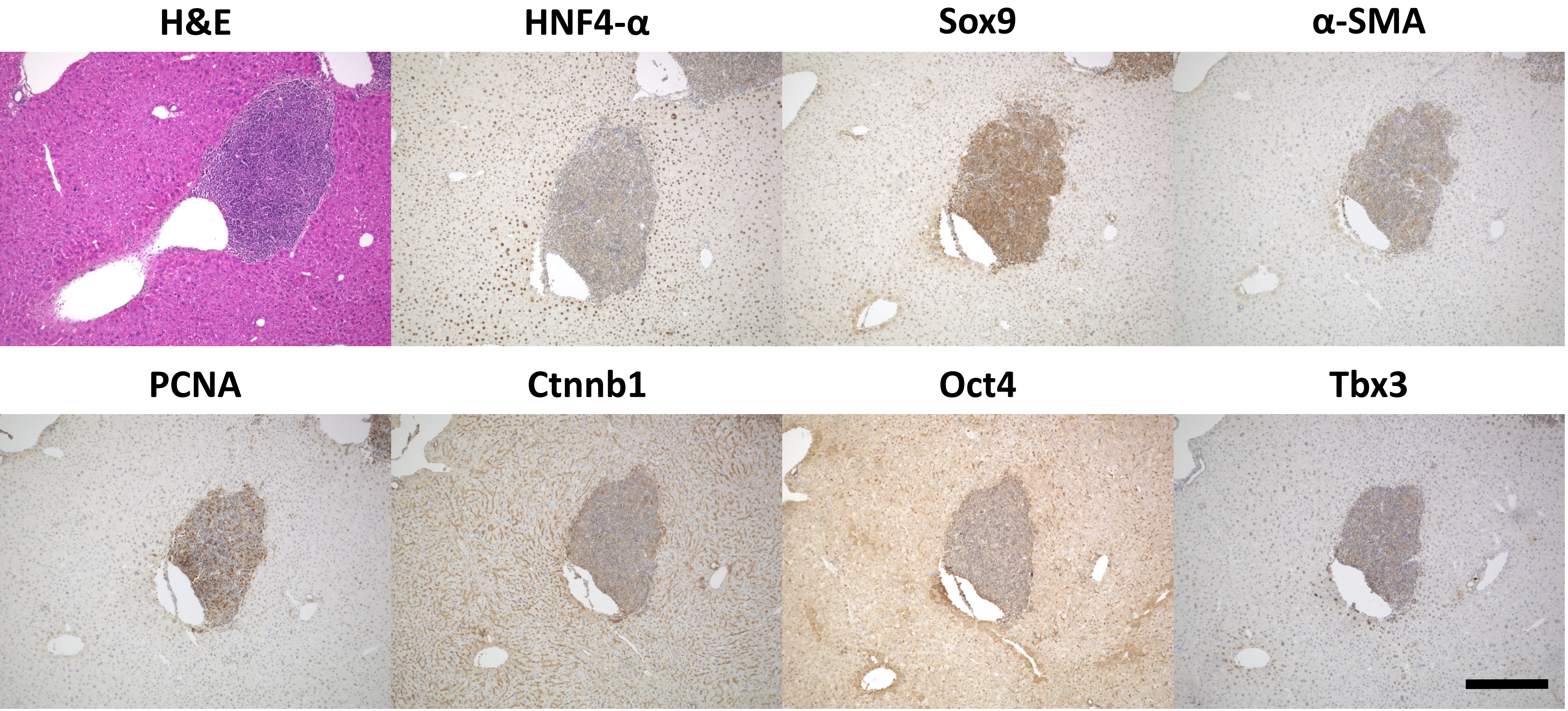

scale = 100 µm

HLN 6 – 20x lens

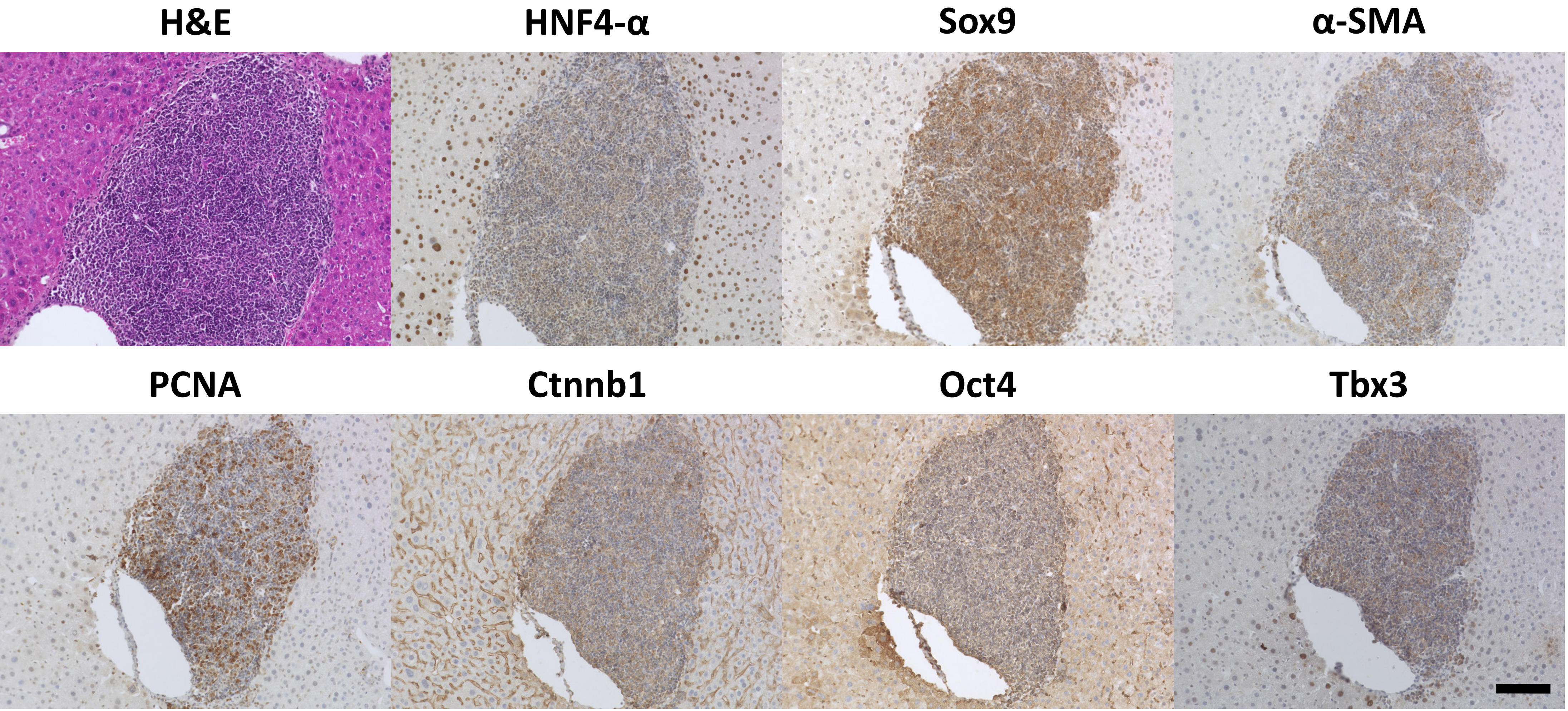

scale = 100 µm

HLN 7 – 10x lens

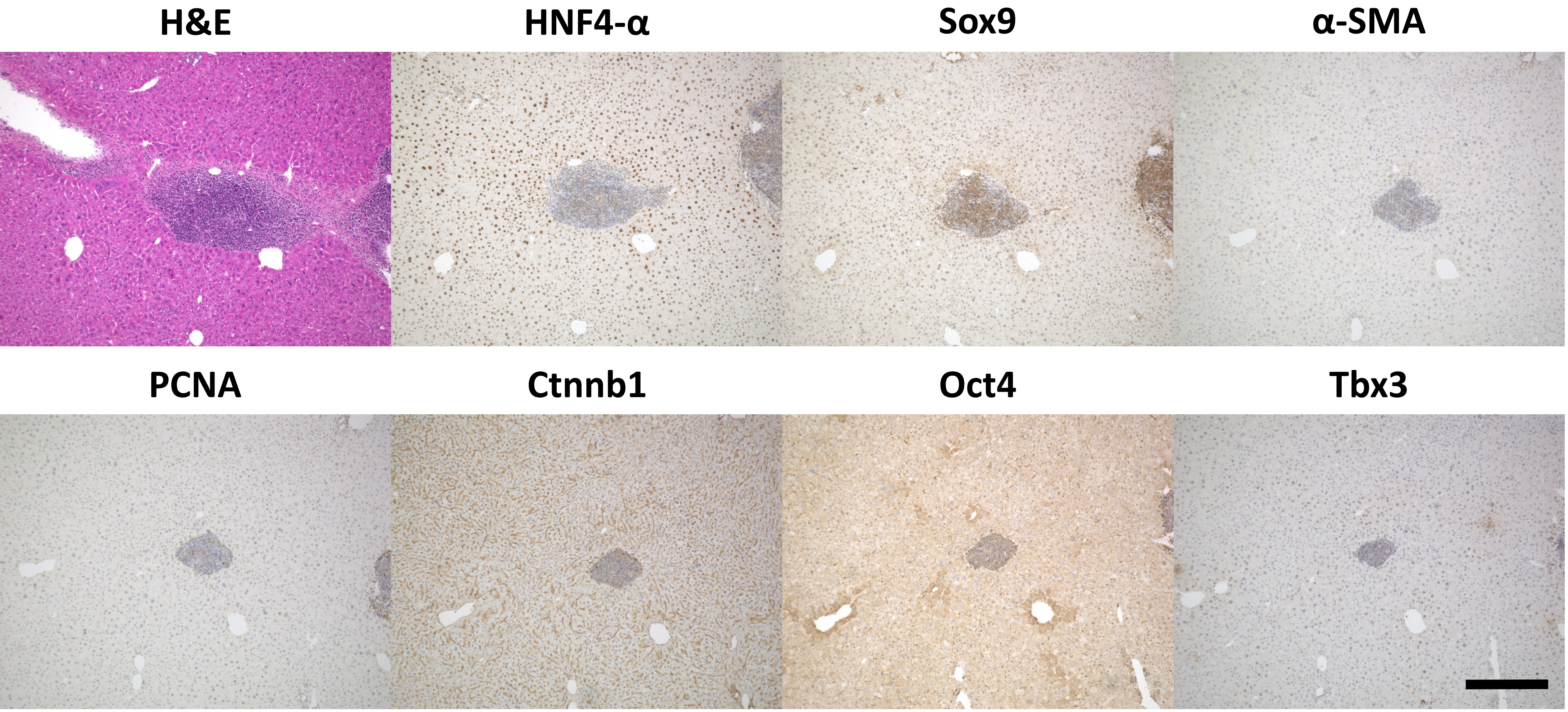

scale = 100 µm

HLN 7 – 20x lens

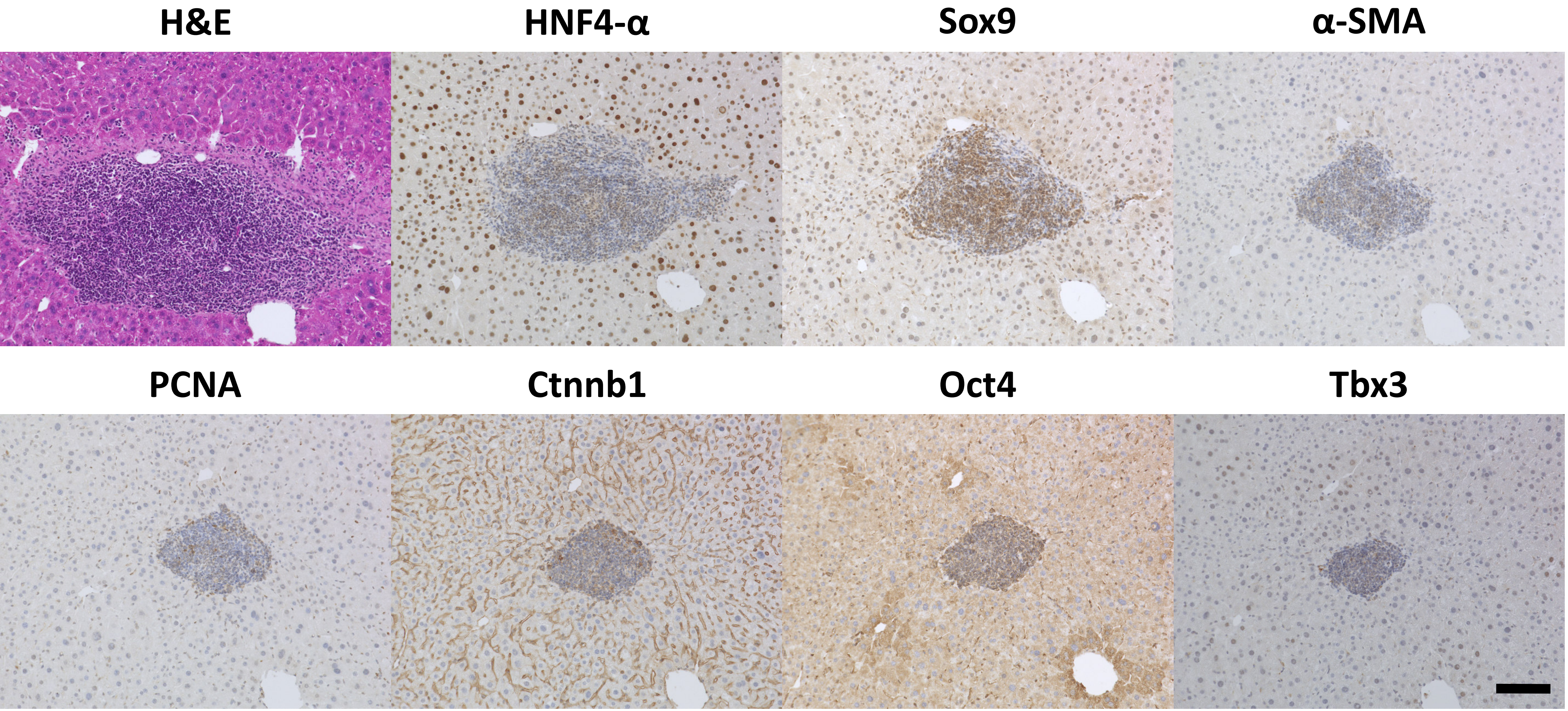

scale = 100 µm

HLN 8 – 10x lens

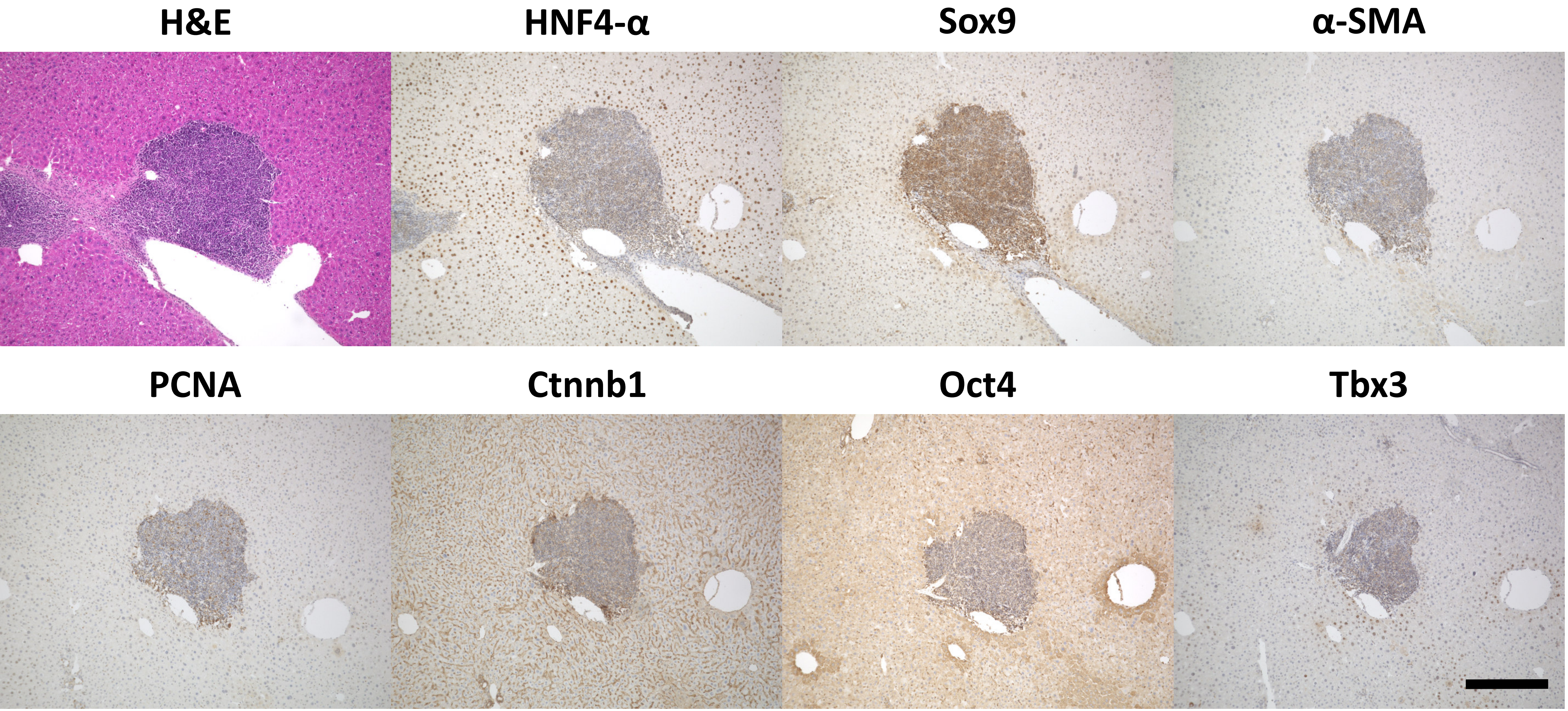

scale = 100 µm

HLN 8 – 20x lens

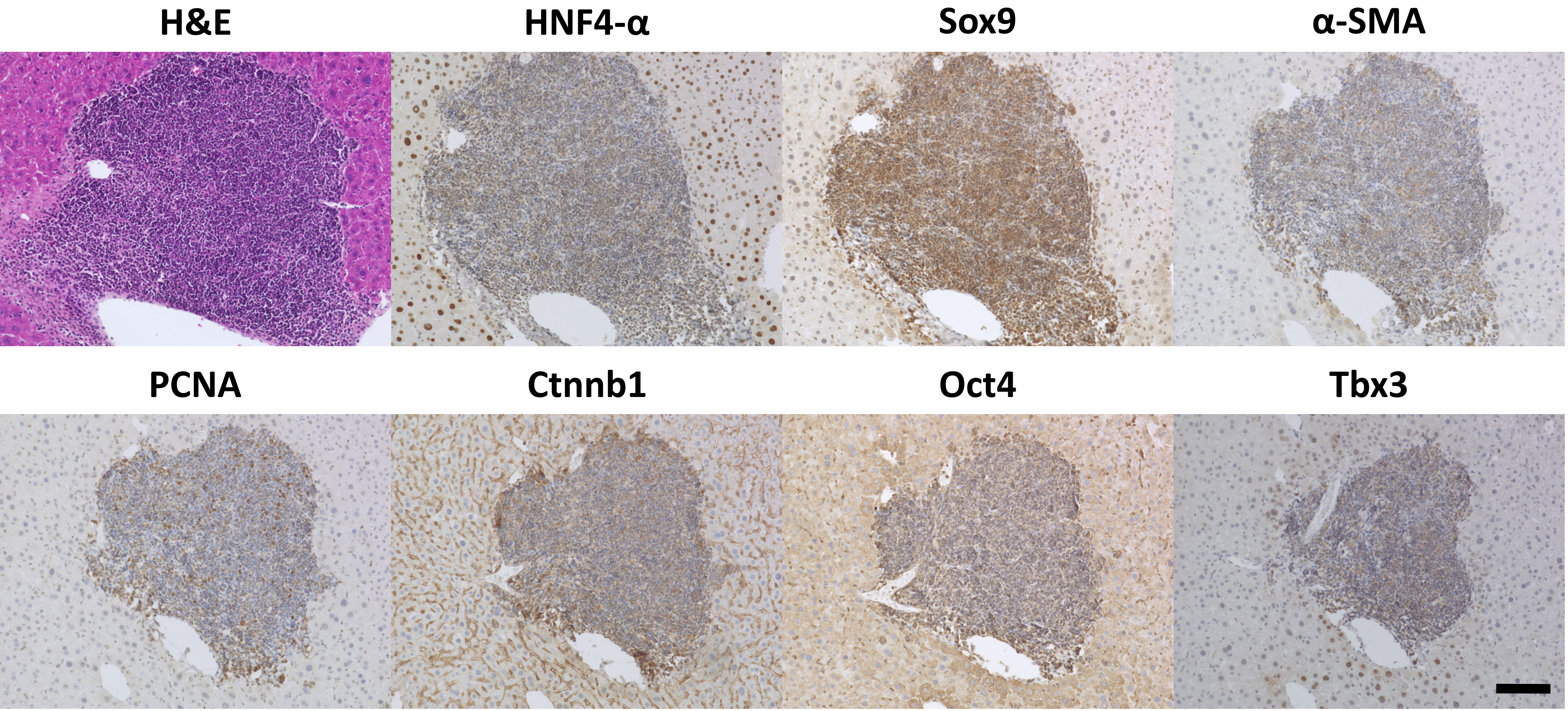

scale = 100 µm

HLN 9 – 10x lens

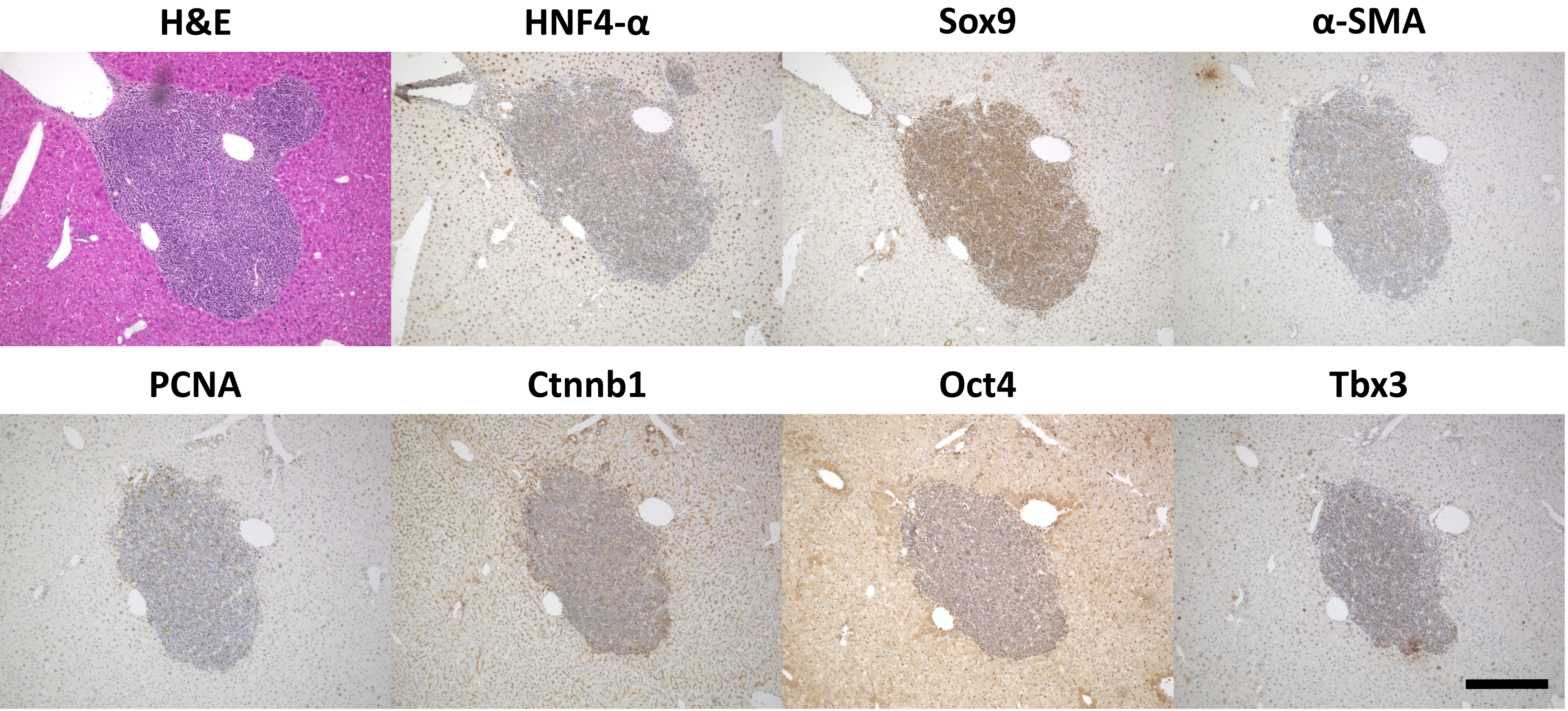

scale = 100 µm

HLN 9 – 20x lens

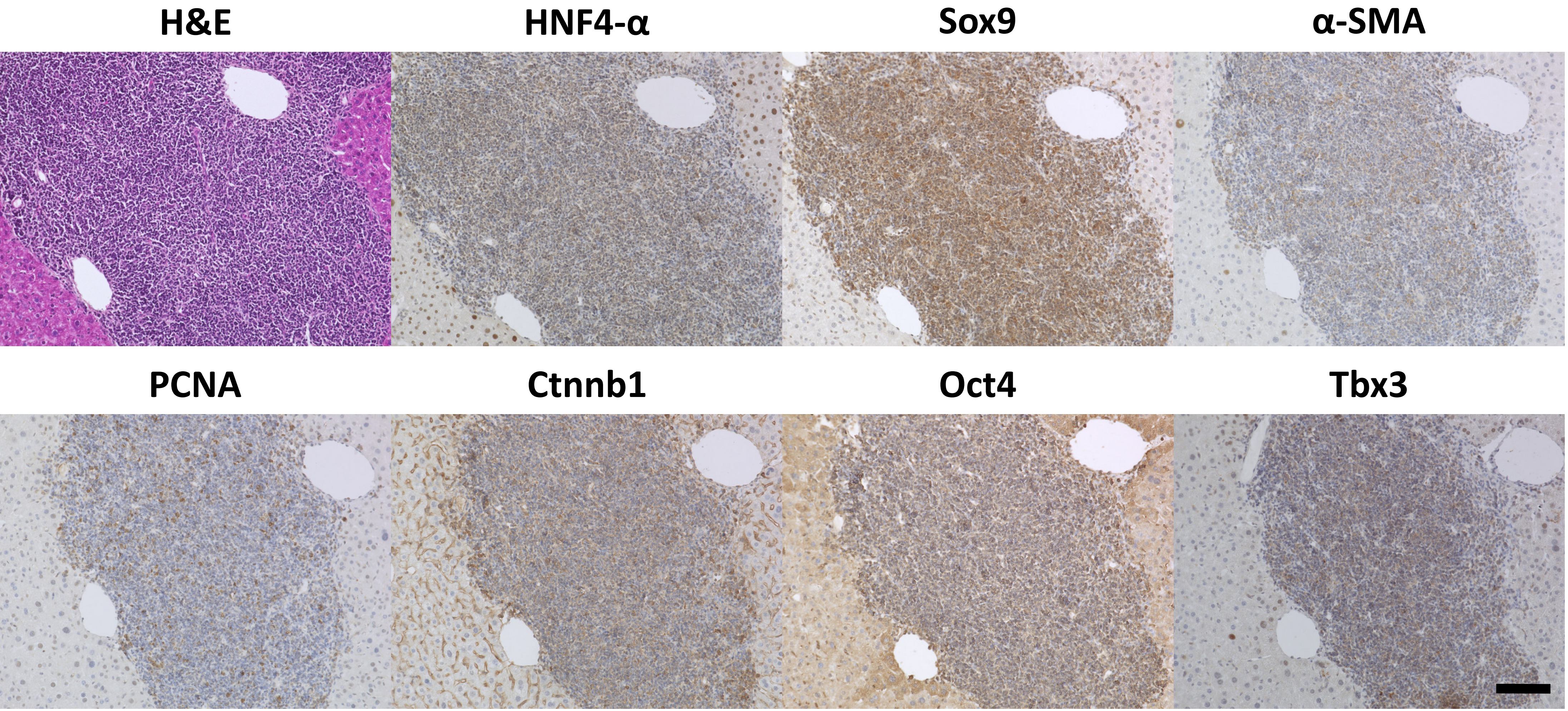

scale = 100 µm

HLN 10 – 10x lens

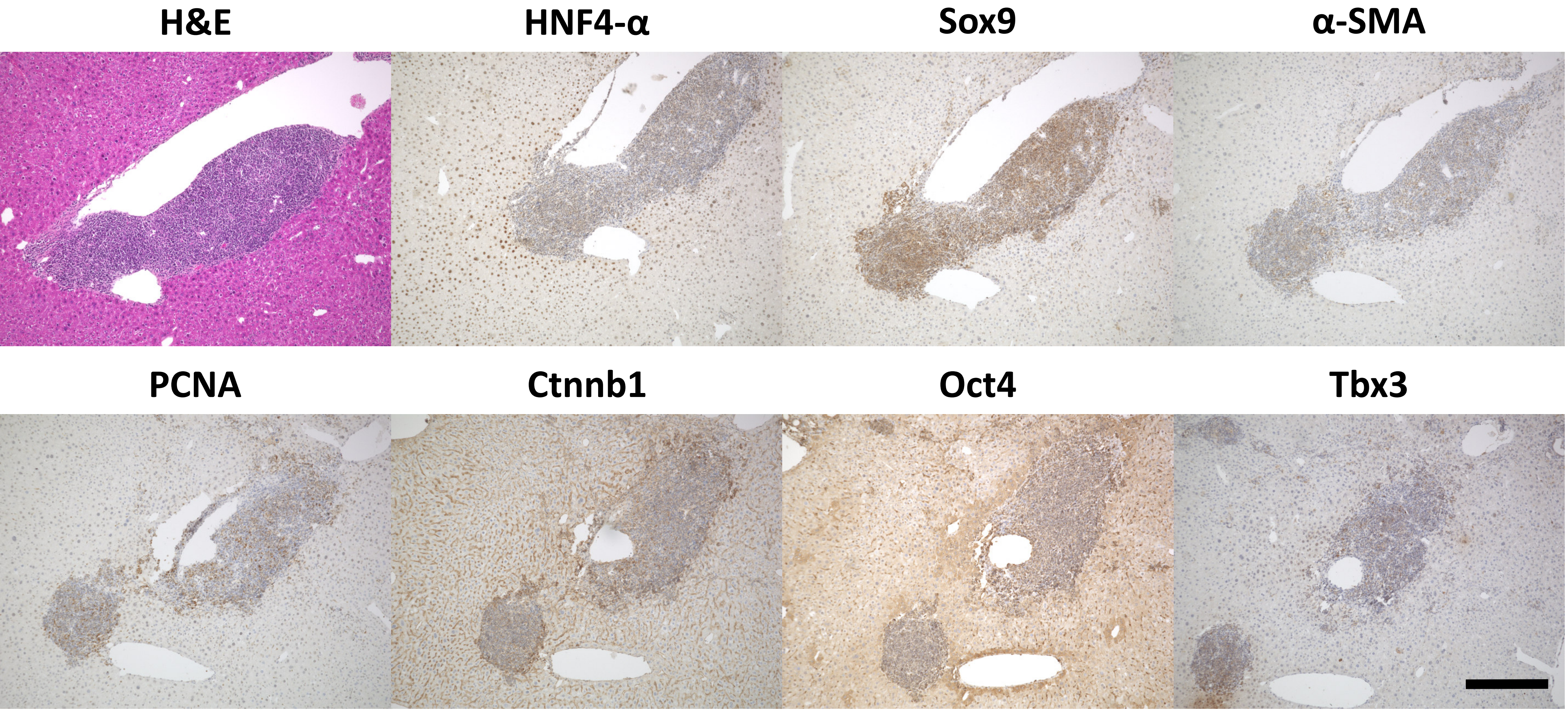

scale = 100 µm

HLN 10 – 20x lens

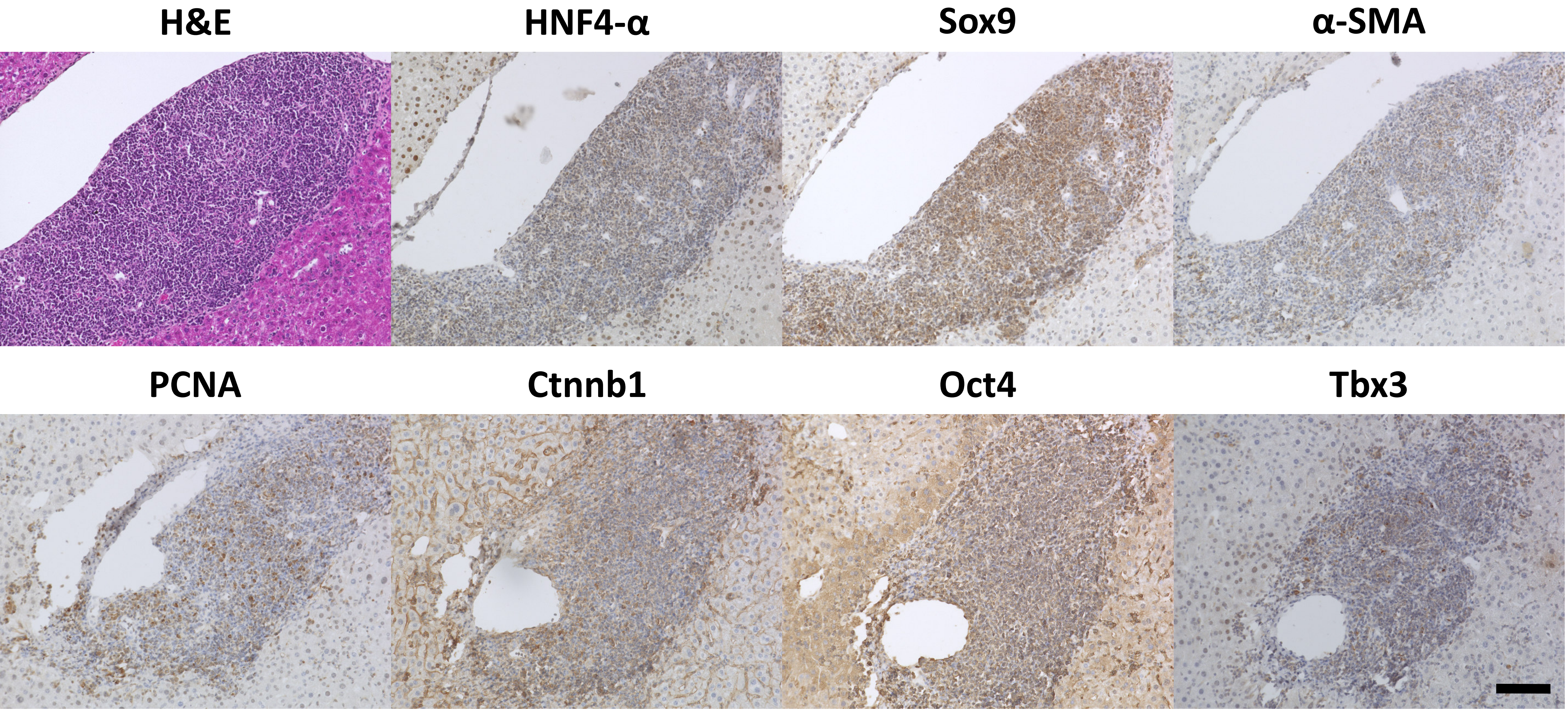

scale = 100 μm

HLN 11 – 10x lens

H&E	HNF4-α	Sox9	α-SMA

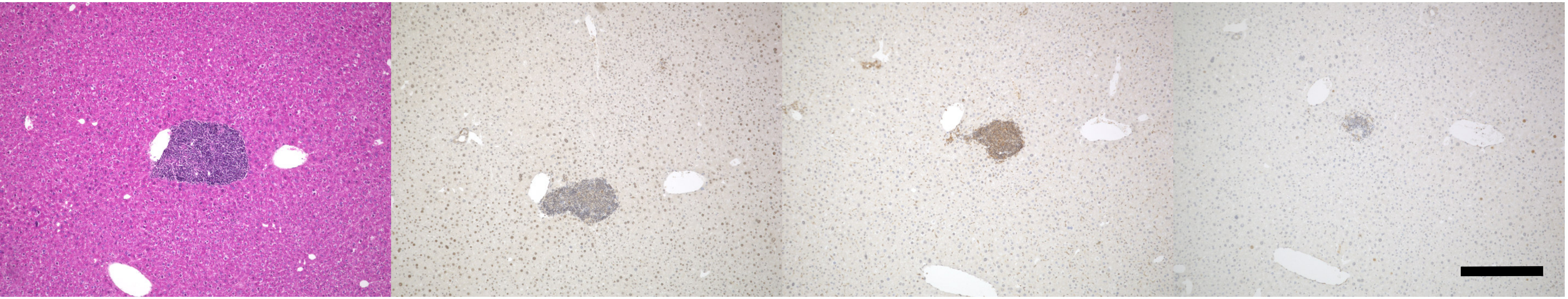

PCNA	Ctnnb1	Oct4	Tbx3
- HLN not on this section -	- HLN not on this section -	- HLN not on this section -	- HLN not on this section -

scale = 100 µm

HLN 11 – 20x lens

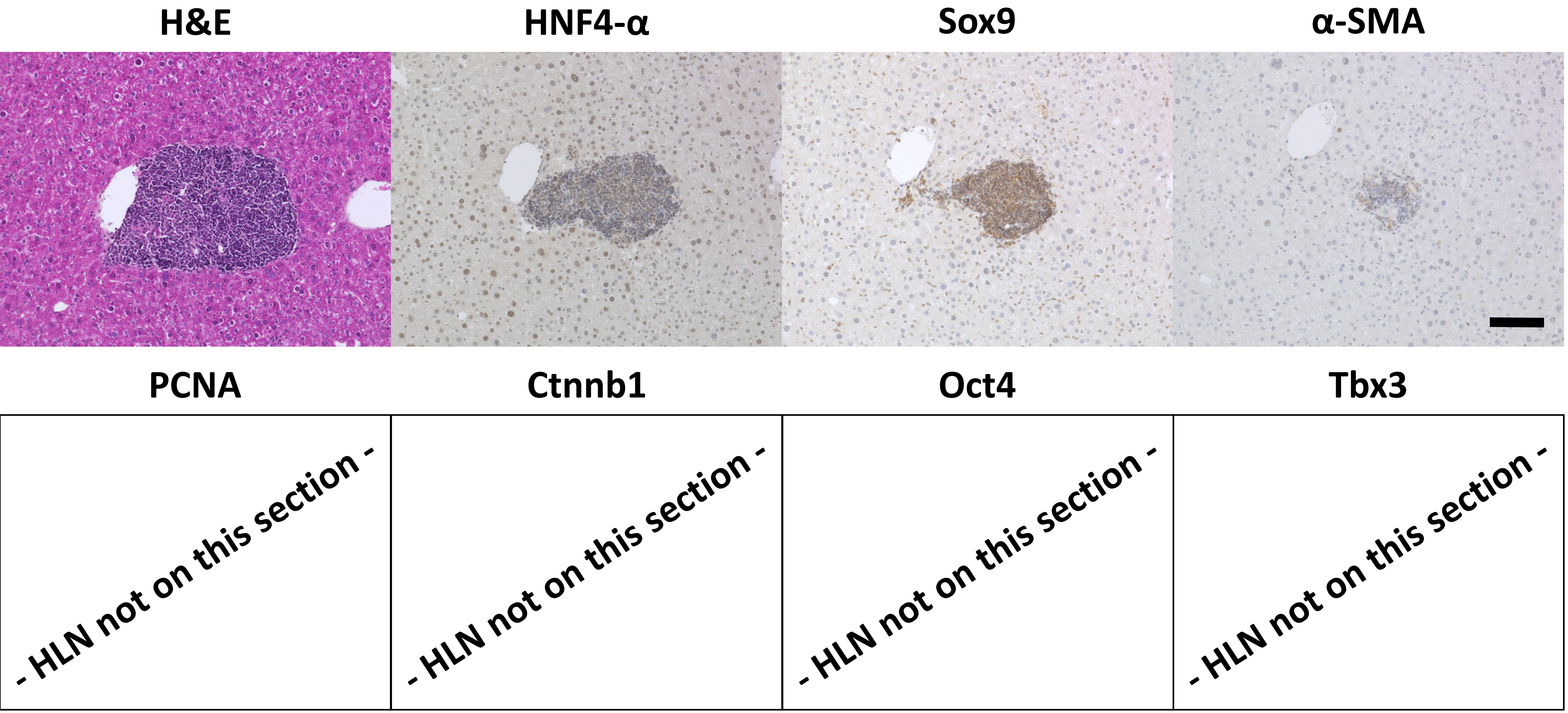

scale = 100 µm

HLN 12 – 10x lens

H&E	HNF4-α	Sox9	α-SMA

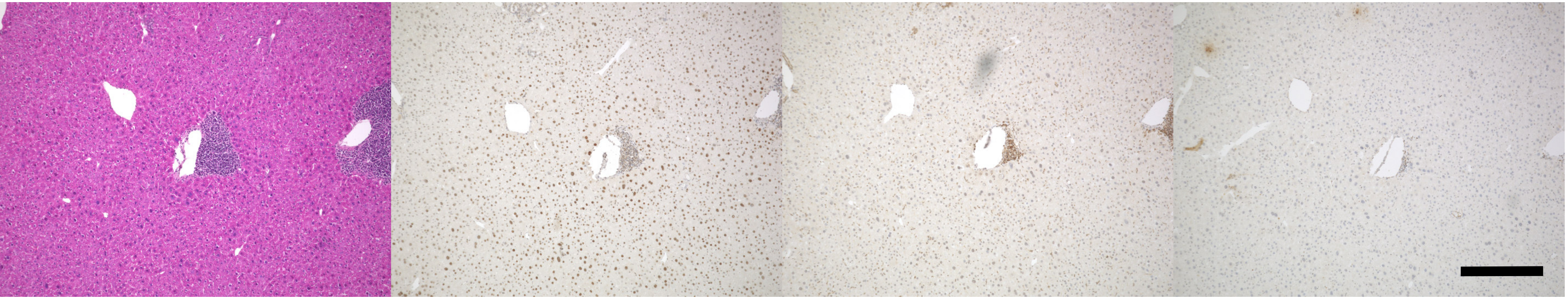

PCNA	Ctnnb1	Oct4	Tbx3
- HLN not on this section -	- HLN not on this section -	- HLN not on this section -	- HLN not on this section -

scale = 100 µm

HLN 12 – 20x lens

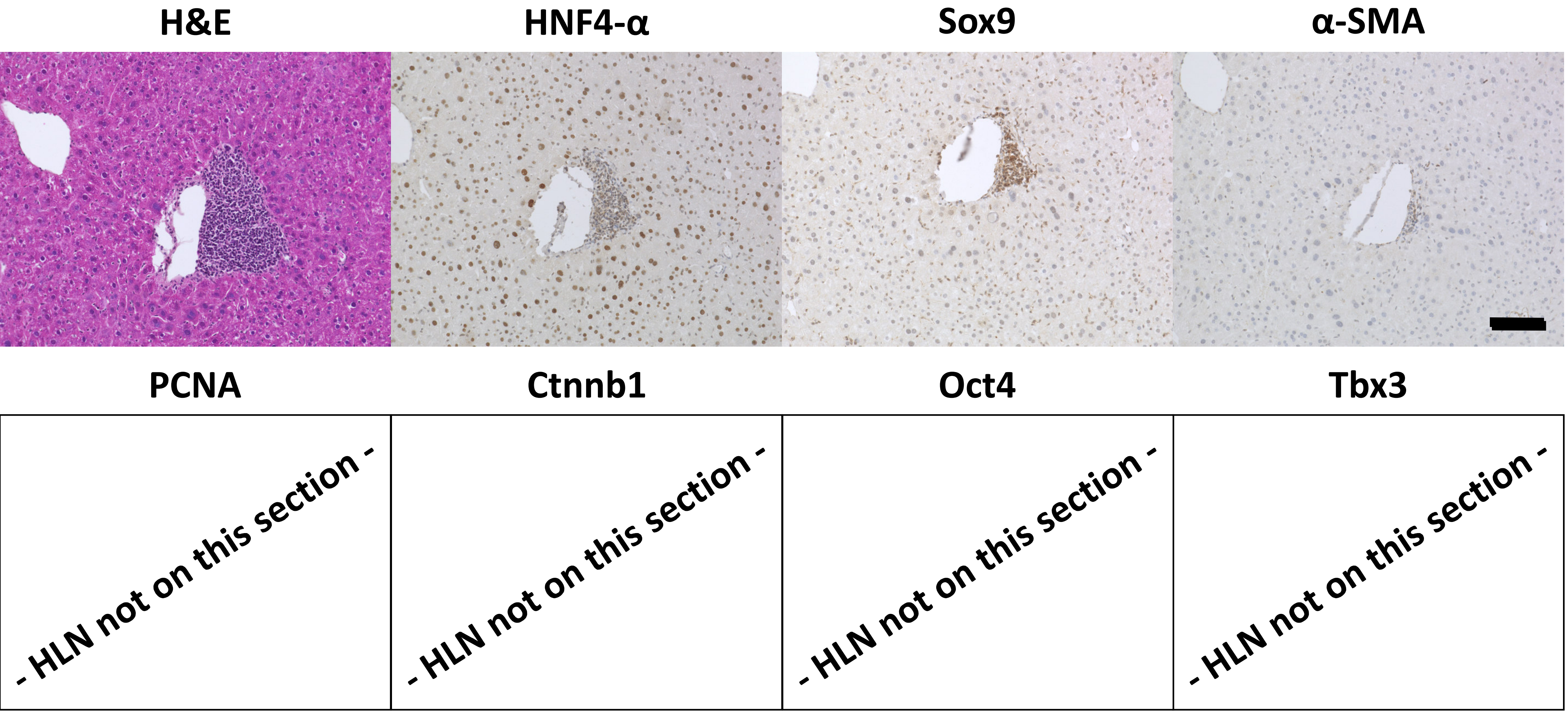

scale = 100 µm

HLN 13 – 10x lens

H&E | HNF4-α | Sox9 | α-SMA

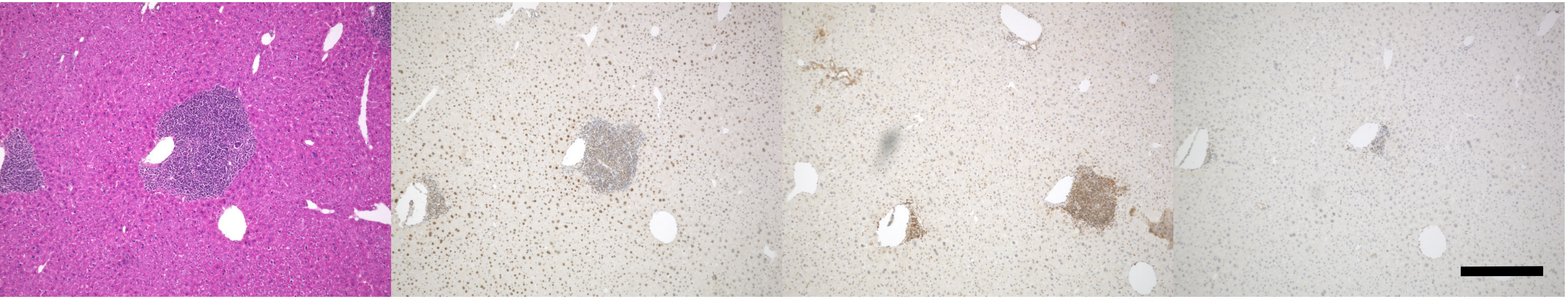

PCNA | Ctnnb1 | Oct4 | Tbx3

- HLN not on this section -

- HLN not on this section -

- HLN not on this section -

- HLN not on this section -

scale = 100 µm

HLN 13 – 20x lens

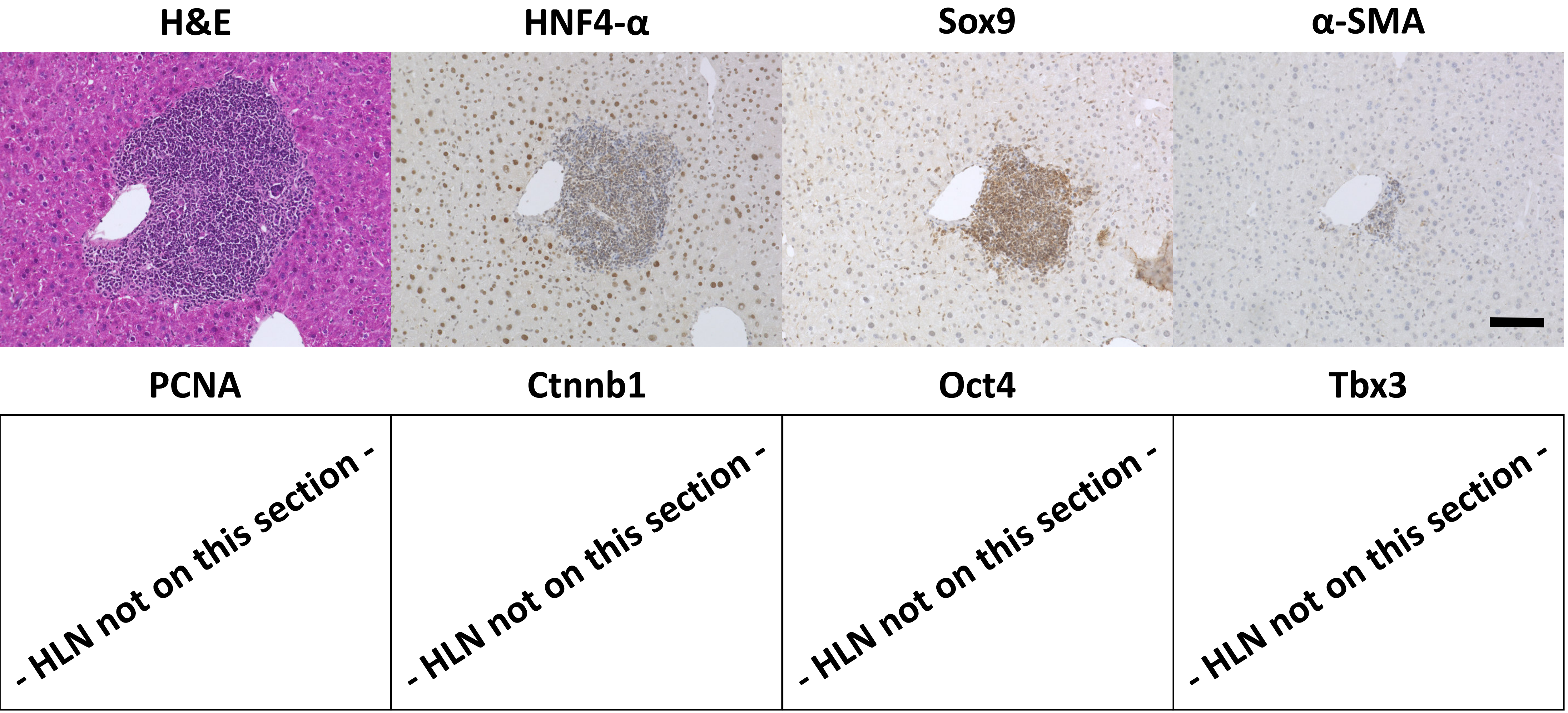

scale = 100 µm

HLN 14 – 10x lens

H&E

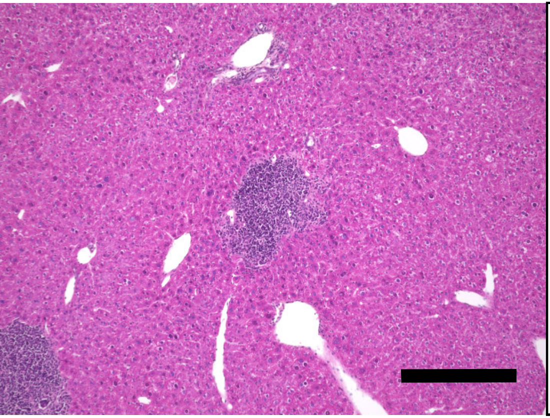

HNF4-α

- HLN not on this section -

Sox9

- HLN not on this section -

α-SMA

- HLN not on this section -

PCNA

- HLN not on this section -

Ctnnb1

- HLN not on this section -

Oct4

- HLN not on this section -

Tbx3

- HLN not on this section -

scale = 100 µm

HLN 14 – 20x lens

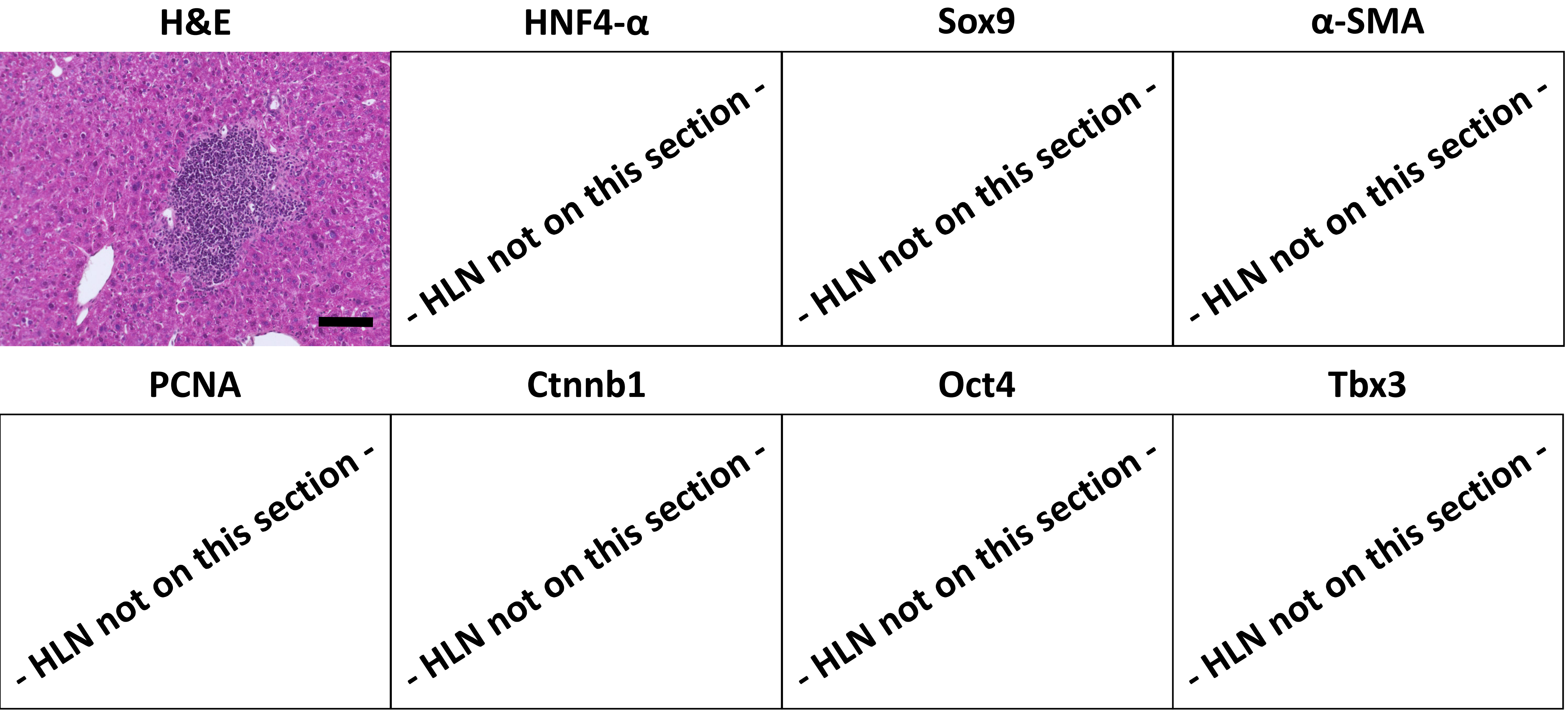

scale = 100 µm
