# Supplementary material for: Nrf2 induces malignant transformation of hepatic progenitor cells by inducing β-catenin expression
Source: Redox Biol. 2022 Sep 13;57:102453. doi: 10.1016/j.redox.2022.102453 (PMC9618468; doi:10.1016/j.redox.2022.102453)
Supplement: Multimedia component 2 [file mmc2.docx]

Nrf2 induces malignant transformation of hepatic progenitor cells by inducing β-catenin expression

Athanassios Fragoulis^1#^, Julia Schenkel^1#^, Nicole Schröder^1^, Elisa Fabiana Brandt^2^, Mathias Weiand^1^, Tabita Neu^1^, Pierluigi Ramadori^3^, Tim Caspers^1^, Sebastian Kant^4^, Thomas Pufe^1^, Antje Mohs^2^, Christian Trautwein^2^, Thomas Longerich^5^, Konrad Ludwig Streetz^2^, and Christoph Jan Wruck^1*^

**Supplementary Material**

**Supplementary Figures:**


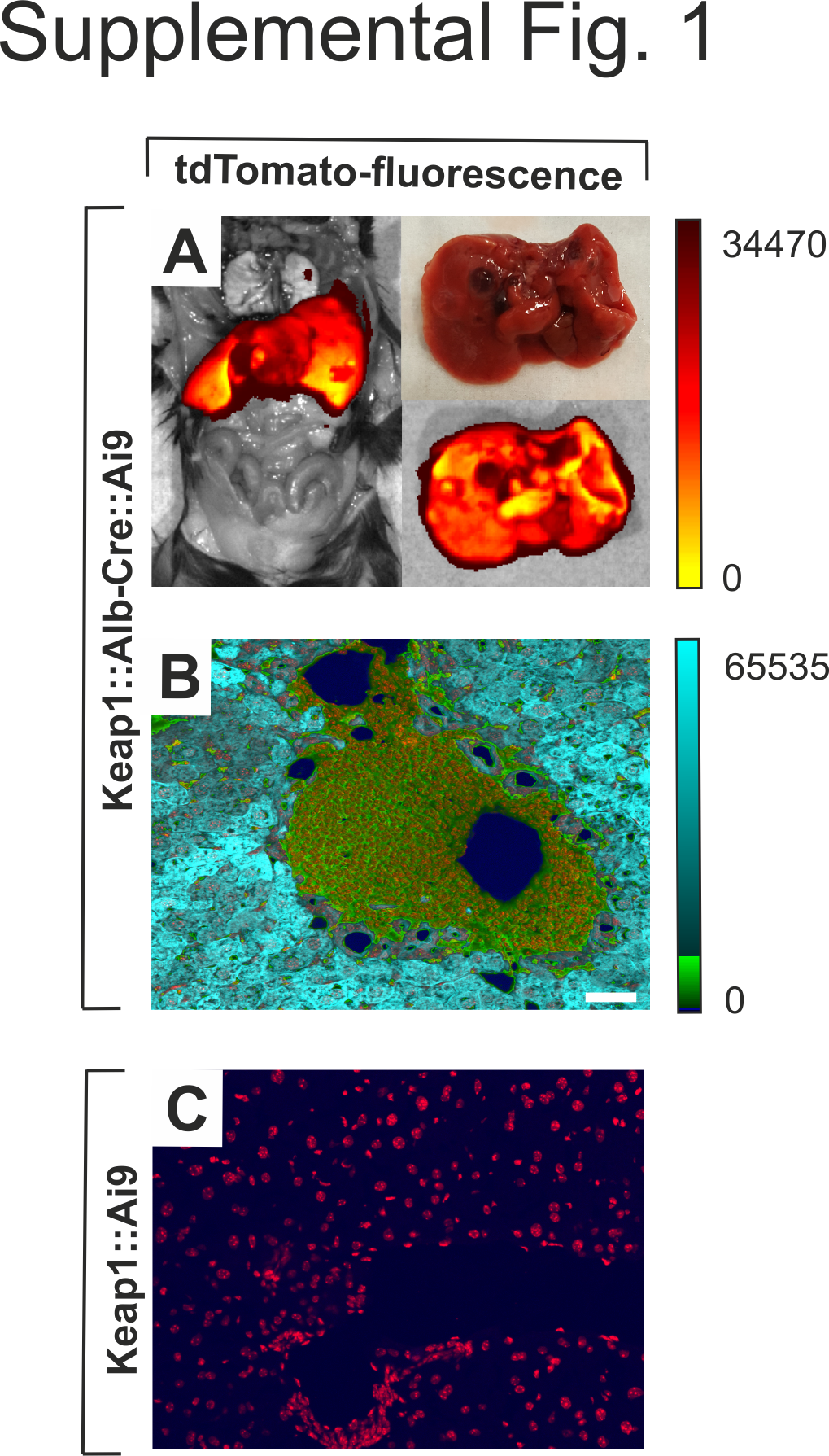


**Supplemental Figure 1.** (*A*) Representative image of *post mortem* tdTomato fluorescence measurements from Keap1::Alb-Cre::Ai9 mice after opening the thoracic and abdominal cavities as well as after preparation of the liver. (n=3). Fluorescence intensities (relative fluorescence units = RFU) are shown using a false color scale. These analyses confirmed that Cre recombinase is expressed exclusively in mouse liver, as no tdTomato signal was detected in other tissues. (*B*) Representative image of fluorescence measurements of liver tissue sections derived from Keap1::Alb-Cre::Ai9 mice (n=3). Resulting confocal images were color-coded blue for no, green for low and cyan for high tdTomato fluorescence. Nuclei are shown in red. Please note that all hepatocytes show high intensity of tdTomato fluorescence and HLN-cells presented a low intensity of tdTomato fluorescence. This indicates at least some Alb-Cre activity within HLN-cells. (Scale bar = 50μm). (*C*) Fluorescence imaging of liver tissue sections derived from Keap1::Ai9 mice without Cre-expression (Cre-negative). Color-coding and imaging parameters were identical to parameters in B. Please note the lack of dtTomato fluorescence in this control image.


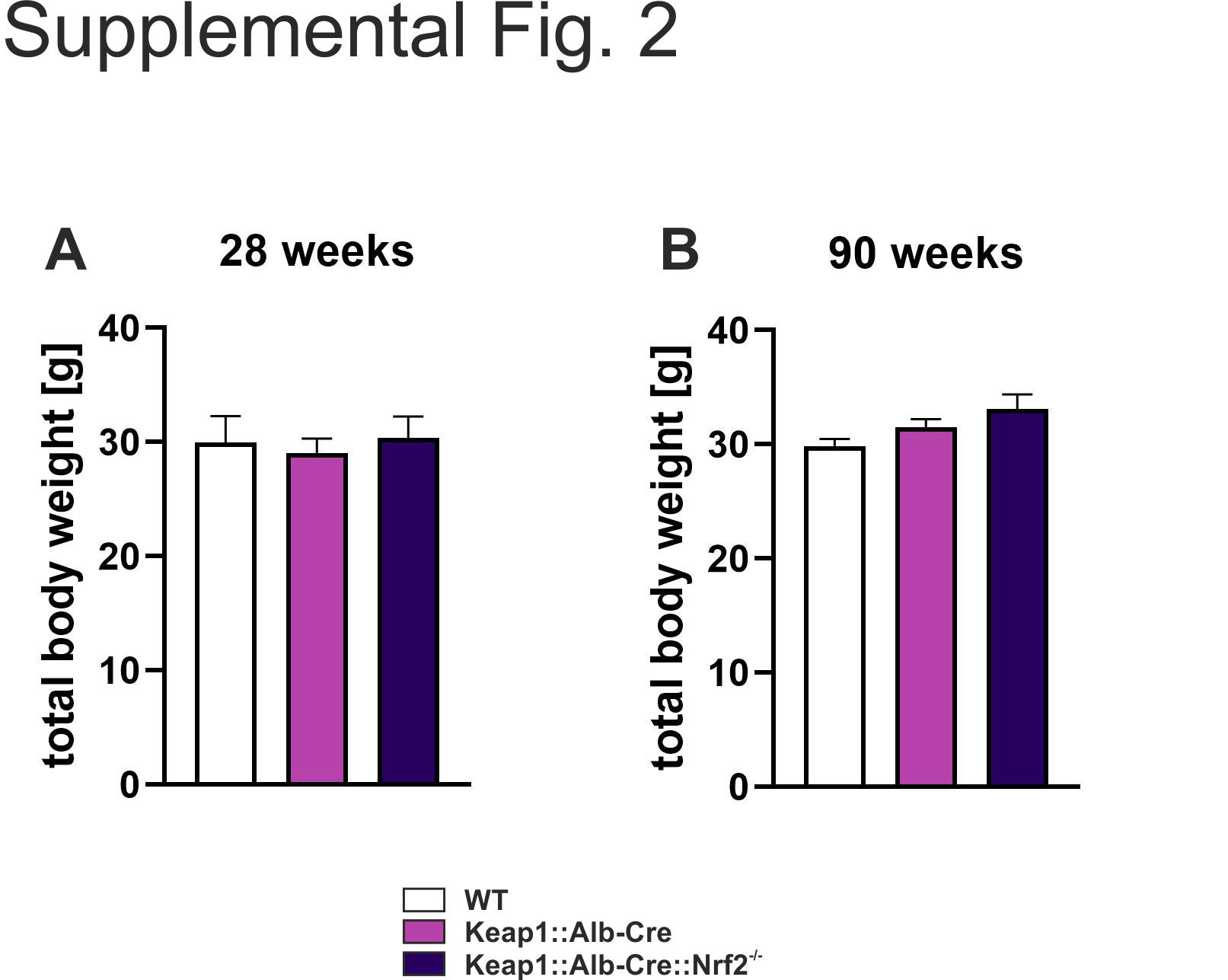


**Supplemental Figure 2.** Statistical analysis of the total body-weight of 28 *(A)* - and 90 *(B)* -week-old wild-type (WT), Keap1::Alb-Cre and Keap1::Alb-Cre::Nrf2^-/-^ mice. Data represent mean ± SEM, one-way ANOVA with the Bonferroni post hoc test; biological replicates were WT mice: n = 21; Keap1::Alb-Cre mice: n = 27; Keap1::Alb-Cre:Nrf2^-/-^ mice: n = 7, * p < 0.05, ** p < 0.005 *** p < 0.001 as indicated.


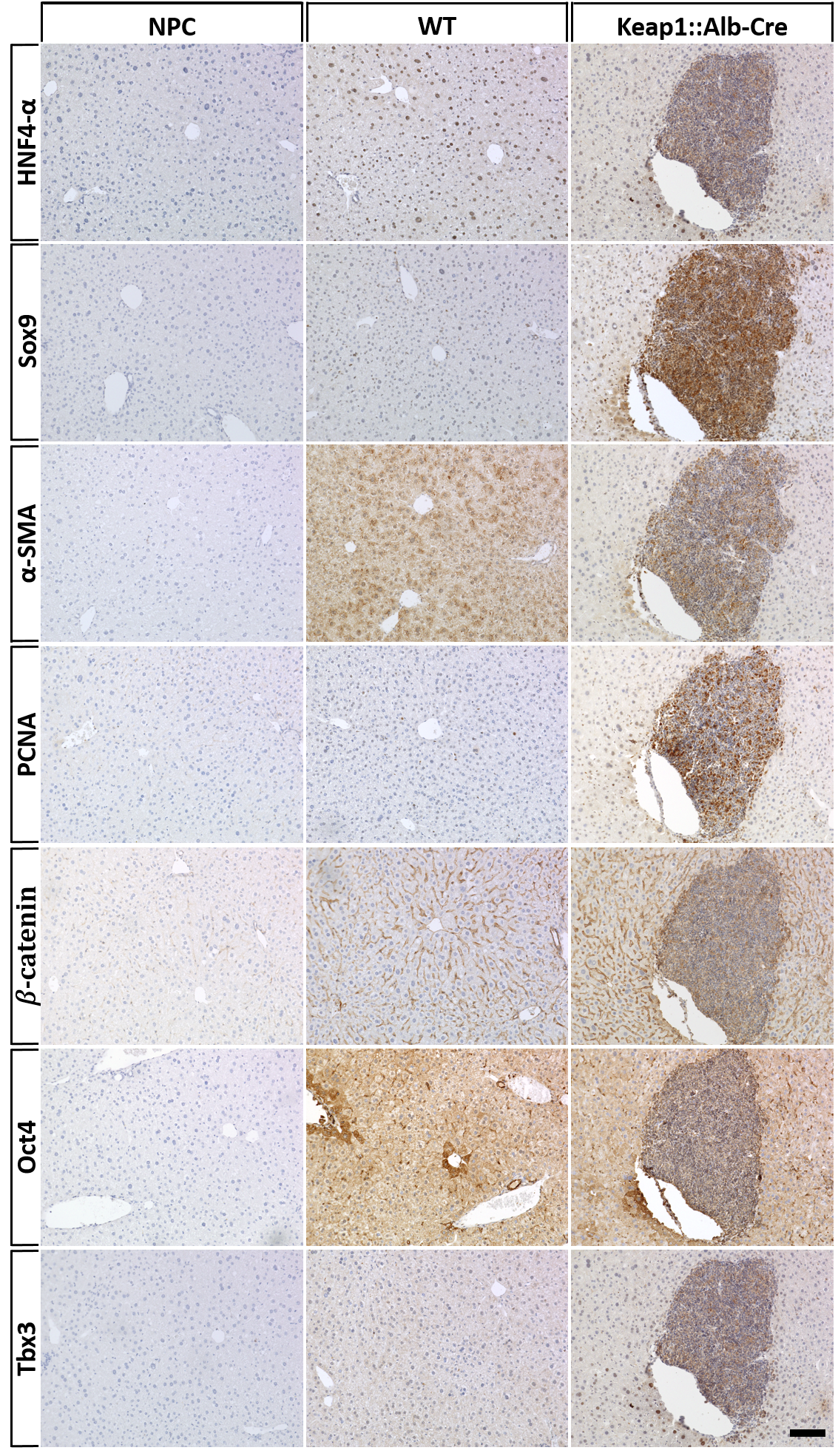


**Supplemental Figure 3.** Additional immunohistochemical analyses for representative markers of each category shown in Fig. 2 (epithelia: HNF4-α; stem and progenitor cells: Sox9, Oct4 and Tbx3; mesenchyme: α-SMA; proliferation: PCNA; hepatoblastoma: β-catenin). Due to the use of consecutive sections, these analyses confirm the co-expression of investigated markers in the same HLN.


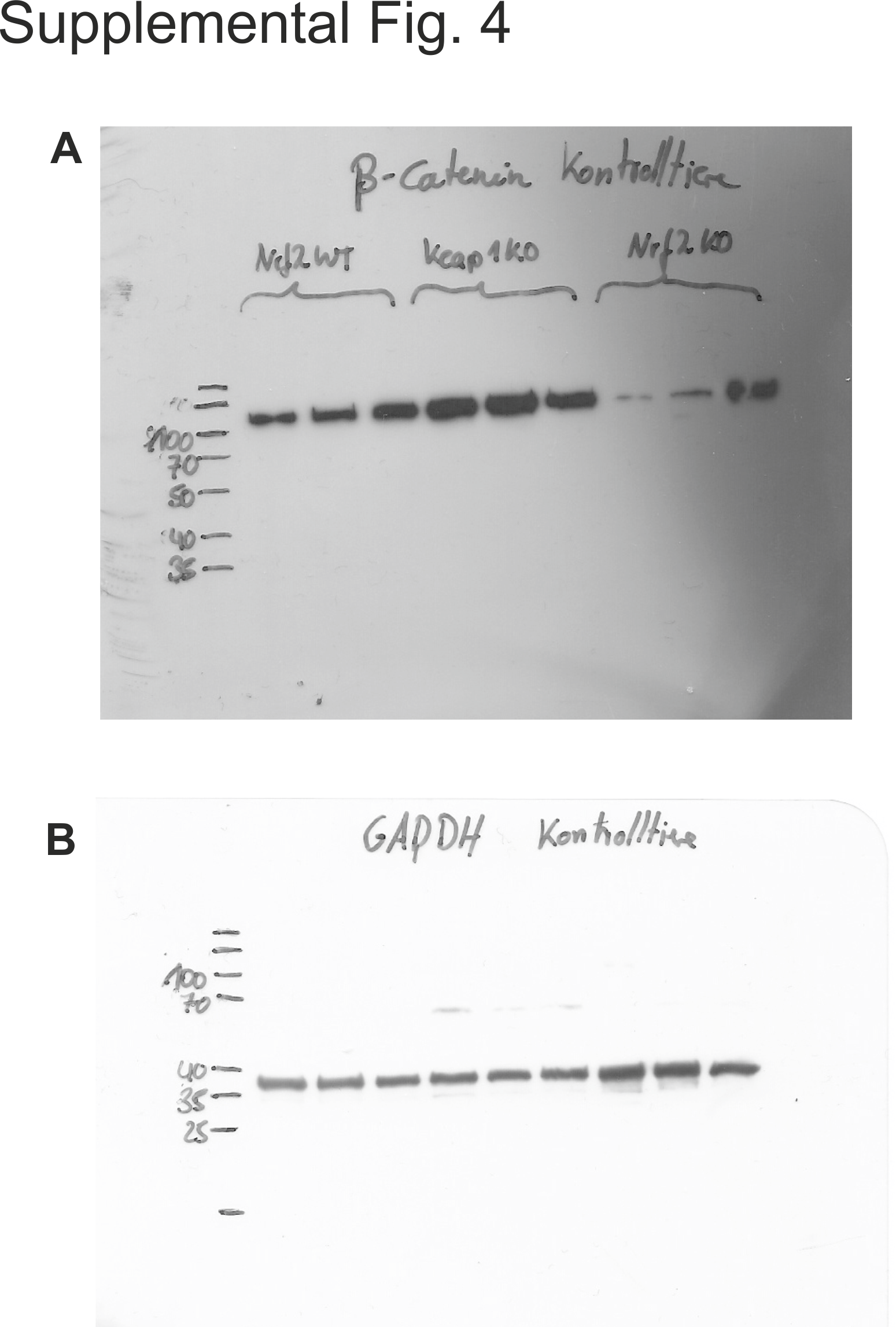


**Supplemental Figure 4.** *(A)* Full blot images of whole liver extracts for β‑catenin (92 kD) protein expression from WT (Nrf2 WT), Keap1::Alb-Cre (Keap1 KO), and Keap1::Alb-Cre::Nrf2^-/-^ (Nrf2 KO) mice. (B) Full blot images for GAPDH (36 kD) protein expression used as loading control.


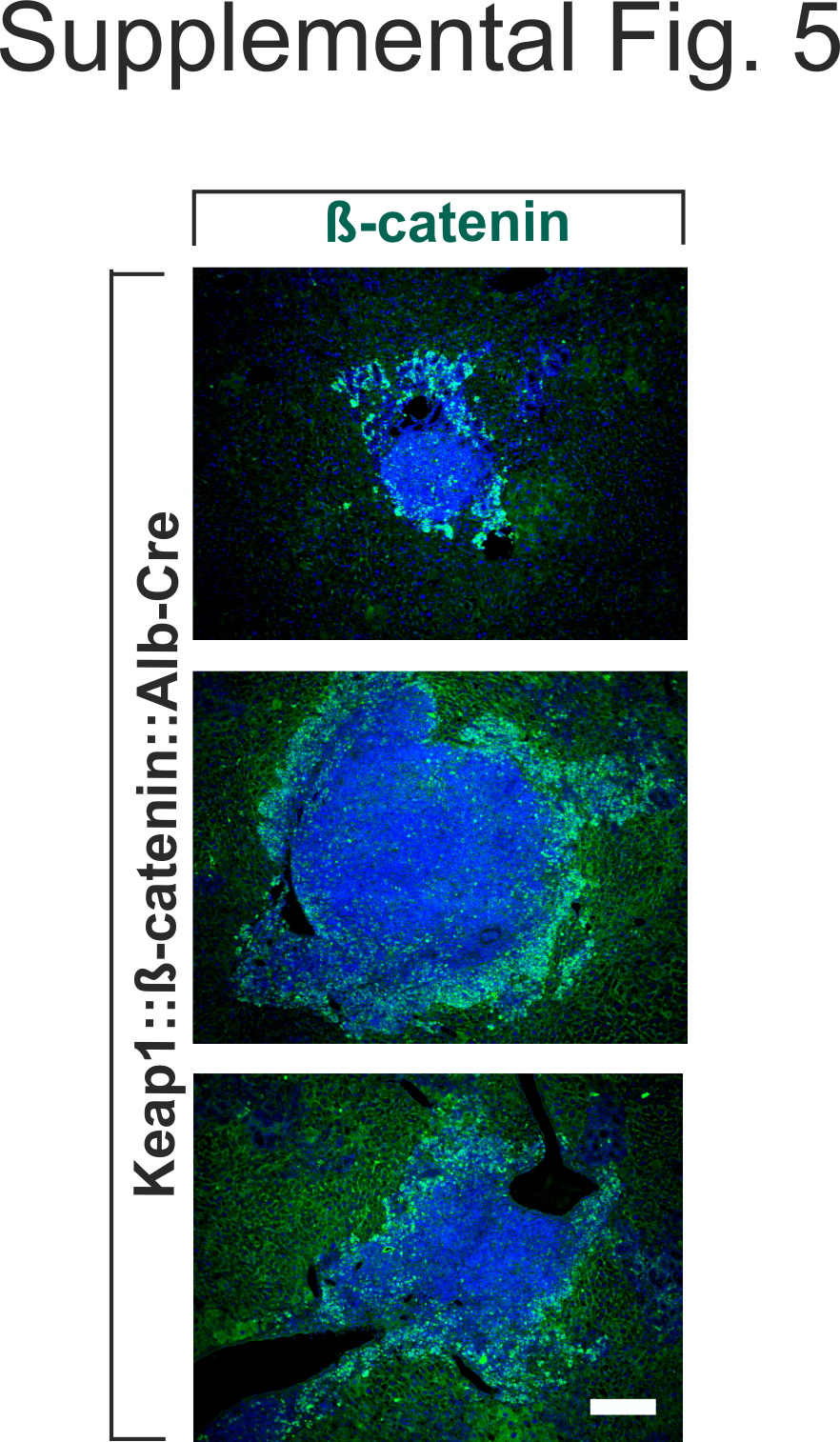


**Supplemental Figure 5.** Liver sections from Keap1::β‑catenin::Alb-Cre mice were immunofluorescence-stained with antibodies against β‑catenin. (Scale bar = 200 µm).

**Supplementary tables:**

| **primary antibody** | **company** | **Cat.#** | **application** | **dilution** |
| --- | --- | --- | --- | --- |
| A6 | provided by Dr. Valentina Factor |  | IHC | 1:100 |
| Actin | Santa Cruz | SC-1616 | WB | 1:4000 |
| α-fetoprotein | Santa Cruz | Sc-8108 | IHC | 1:500 |
| αSMA | Abcam | ab5694 | IHC | 1:500 |
| β‑catenin | BD Biosciences | 610154 | WB  IHC | 1:000  1:250 |
| E-Catherin | Progen Biotech | CD324 | IHC | 1:200 |
| GAPDH | Genetex | GTX627408-01 | WB | 1:4000 |
| Glutamine Synthetase | Merk | G2781 | IHC | 1:200 |
| HNF4α | Santa Cruz | Sc6556 | IHC | 1:400 |
| Keap1 | Proteintech | 10503-2-AP | IHC | 1:200 |
| Lin28A | Genetex | GTX300027 | IHC | 1:600 |
| Nrf2 [EPR25A] | Abcam | ab172730 | IHC | 1:1000 |
| Nrf2 [H-300x] | Santa Cruz | sc-13032 X | WB  ChIP | 1:750  5µg |
| Oct4 | Genetex | GTX300027 | IHC | 1:200 |
| Pan-CK | DAKO | ZO622 | IHC | 1:400 |
| PCNA [PC10] | Abcam | Ab29 | IHC | 1:20000 |
| Sox9 | Bioworld | BS1597 | IHC | 1:500 |
| Tbx3 | Thermo Fischer | 42-4800 | IHC | 1:250 |
| Vimentin | MyBiosource | MBS535183 | IHC | 1:100 |

**Table S1:** List of Antibodies used in this study

| Table S2: List of primers used for RT-(q)PCR | | | | | | |
| --- | --- | --- | --- | --- | --- | --- |
| Target type | Gene (synonym) | Sequence accession number (LRG) | Direction | Sequence | Annealing temperature [°C] | Amplicon  length  [bp] |
| REF | Actb | NM_007393.5 | for | CACTGTCGAGTCGCGTCC | 60.0 | 89 |
|  |  |  | rev | TCATCCATGGCGAACTGGTG |  |  |
|  | B2m | NM_009735.3 | for | TTCTGGTGCTTGTCTCACTGA | 61.0 | 104 |
|  |  |  | rev | CAGTATGTTCGGCTTCCCATTC |  |  |
|  | Eef2 | NM_007907.2 | for | TCACAATCAAATCCACCGCC | 60.0 | 122 |
|  |  |  | rev | ATGGCCTGGAGAGTCGATGA |  |  |
|  | Gapdh | NM_008084.3 | for | CATGGCCTTCCGTGTTCCTA | 60.0 | 74 |
|  |  |  | rev | ACTTGGCAGGTTTCTCCAGG |  |  |
|  | Hprt | NM_013556.2 | for | TCAGTCAACGGGGGACATAAA | 61.0 | 142 |
|  |  |  | rev | GGGGCTGTACTGCTTAACCAG |  |  |
|  | Rpl13a | NM_009438.5 | for | GCGGATGAATACCAACCCCT | 61.0 | 179 |
|  |  |  | rev | CCACCATCCGCTTTTTCTTGT |  |  |
|  | Sdha | NM_023281.1 | for | GGAACACTCCAAAAACAGACCT | 60.0 | 106 |
|  |  |  | rev | CCACCACTGGGTATTGAGTAGAA |  |  |
|  | Tbp | NM_013684.3 | for | ATCTACCGTGAATCTTGGCTGT | 61.0 | 183 |
|  |  |  | rev | GATTGTTCTTCACTCTTGGCTC |  |  |
|  | Tubb4b | NM_146116.2 | for | TCTTCTACAGCTGTTCCGCAG | 61.0 | 143 |
|  |  |  | rev | GTGGTAAGTGCCAGTGGGAT |  |  |
|  | Ywhaz | NM_011740 | for | GAAAAGTTCTTGATCCCCAATGC | 62.0 | 134 |
|  |  |  | rev | TGTGACTGGTCCACAATTCCTT |  |  |
| GOI | Afp | NM_007423.4 | for | AGAACCTGCCGAGAGTTGC | 60.0 | 199 |
|  |  |  | rev | GGATGCTCTCTTTGTCTGGAAGC |  |  |
|  | Alb  (Albumin) | NM_009654.4 | for | TCCTGGGCACGTTCTTGTAT | 58.5 | 270 |
|  |  |  | rev | TGCTTTCTGGGTGTAGCGAA |  |  |
|  | Cd44 | NM_001039151.1 | for | CAGAGGCGACTAGATCCCTC | 59.0 | 762 |
|  |  |  | rev | GAGTCACAGTGCGGGAACTC |  |  |
|  | Cd133 | NM_001163577.1 | for | CTCCCATCAGTGGATAGAGAACT | 60.0 | 81 |
|  |  |  | rev | ATACCCCCTTTTGACGAGGCT |  |  |
|  | Ccnd1  (CyclinD1) | NM_007631.2 | for | GCGTACCCTGACACCAATCT | 60.0 | 160 |
|  |  |  | rev | CACAGACCTCCAGCATCCAG |  |  |
|  | Cdh1 (E-Cadherin) | NM_009864.3 | for | CAGACCCCACGACCAATGAT | 60.0 | 70 |
|  |  |  | rev | TATTGCTGCTTGGCCTCAAA |  |  |
|  | Ctnnb1 (b-Catenin) | NM_001165902.1 | for | CTAGCTGGTGGACTGCAGAAA | 59.0 | 212 |
|  |  |  | rev | TTCAGCACTCTGCTTGTGGT |  |  |
|  | Dlk1 | NM_010052.5 | for | AGTGCGAAACCTGGGTGTC | 60.0 | 148 |
|  |  |  | rev | GCCTCCTTGTTGAAAGTGGTCA |  |  |
|  | EpCAM | NM_008532.2 | for | CATTTGCTCCAAACTGGCGT | 59.0 | 125 |
|  |  |  | rev | TTGTTCTGGATCGCCCCTTC |  |  |
|  | Hnf4 | NM_008261.2 | for | AAGGTGCCAACCTCAATTCATC | 60.5 | 177 |
|  |  |  | rev | CACATTGTCGGCTAAACCTGC |  |  |
|  | Keap1 | NM_001110305.1 | for | GGAATGAGTGGCGGATGATCAC | 59.0 | 398 |
|  |  |  | rev | GCTTCAGCAGGTACAGTTTTG |  |  |
|  | Klf4 | NM_010637.3 | for | TATCAAGAGCTCATGCCACCGGG | 60.0 | 215 |
|  |  |  | rev | TTCCACCCACAGCCGTCCCA |  |  |
|  | Krt19 (Ck-19) | NM_008471.3 | for | GGTGAAGATCCGCGACTGG | 60.0 | 193 |
|  |  |  | rev | GTGTTCTGTCTCAAACTTGGTTCT |  |  |
|  | Myc (cMyc) | NM_001177352.1 | for | GTTGGAAACCCCGCAGACAG | 60.5 | 94 |
|  |  |  | rev | ATAGGGCTGTACGGAGTCGT |  |  |
|  | Nqo1 | NM_008706 | for | AGAGAGTGCTCGTAGCAGGAT | 61.5 | 103 |
|  |  |  | rev | CTACCCCCAGTGGTGATAGAAA |  |  |
|  | Nrf2 | NM_010902.4 | for | CCCAGCAGGACATGGATTTGA | 60.5 | 106 |
|  |  |  | rev | AGCTCATAGTCCTTCTGTCGC |  |  |
|  | Snai1 (Snail) | NM_011427.2 | for | TCTGCACGACCTGTGGAAAG | 60.0 | 125 |
|  |  |  | rev | GTTGGAGCGGTCAGCAAAAG |  |  |
|  | Snai2 (Slug) | NM_011415.2 | for | AGAAGCCCAACTACAGCGAA | 59.5 | 124 |
|  |  |  | rev | ATAGGGCTGTATGCTCCCGA |  |  |
|  | Sox9 | NM_011448.4 | for | GTGAAGAACGGACAAGCGGA | 60.0 | 148 |
|  |  |  | rev | GATTGCCCAGAGTGCTCGC |  |  |
|  | Tgfb1 | NM_011577.1 | for | ACCGCAACAACGCCATCTAT | 59.5 | 232 |
|  |  |  | rev | GTATCAGTGGGGGTCAGCAG |  |  |
|  | Twist1 | NM_011658.2 | for | GCCGGAGACCTAGATGTCATTG | 60.5 | 151 |
|  |  |  | rev | CCACGCCCTGATTCTTGTGA |  |  |
|  | Vim (Vimentin) | NM_011701.4 | for | CCGCTTTGCCAACTACATCG | 62.0 | 239 |
|  |  |  | rev | CCTCCTGCAATTTCTCTCGCA |  |  |

**Table S3:** List of primers used for ChIP

| **primer** |  | **sequence (5‘ → 3‘)** |  | **Product size** | **Target type** |
| --- | --- | --- | --- | --- | --- |
|  |  |  |  |  |  |
| Ctnnb1 ARE | for | CACGCCTGGTTCTACTGACC |  | 126 bp | Unknown |
|  | rev | TGGAGTCCCCAAGAGTGTCT |  |  | sample |
| GST ARE | for | ACTTGGCAGGAAGGATCAGT |  | 120 bp | positive |
|  | rev | TGCTCTAGGTCTCAGTGCAG |  |  | control |
| Actin | for | CCGGTCGAGTCGCGTCCACC |  | 80 bp | negative |
|  | rev | GGCGAACTGGTGGCGGGTGT |  |  | control |

**Table S4:** List of oligos used for EMSA

| **oligo** |  | **sequence (5‘ → 3‘)** |
| --- | --- | --- |
| Ctnnb1 ARE  IRDye700 | s | CTGGTGAACGCAGTGGTGGA**GC**TGAA**TCA**CTTGTTAATAGATGTTCGCAG |
|  | as | CTGCGAACATCTATTAACAAG**TGA**TTCA**GC**TCCACCACTGCGTTCACCAG |
| Ctnnb1 ARE  competitor | s | CTGGTGAACGCAGTGGTGGA**GC**TGAA**TCA**CTTGTTAATAGATGTTCGCAG |
|  | as | CTGCGAACATCTATTAACAAG**TGA**TTCA**GC**TCCACCACTGCGTTCACCAG |
| ARE mutant competitor | s | CTGGTGAACGCAGTGGTGGA**TA**TGAA**GTC**CTTGTTAATAGATGTTCGCAG |
|  | as | CTGCGAACATCTATTAACAAG**GAC**TTCA**TA**TCCACCACTGCGTTCACCAG |

ARE sequence marked in red, mutations are highlighted by yellow backgrounds

**Table S5:** Pipetting scheme for EMSA

| **contents** | **no competitor** | **no nuclear extract** | **competitor** | **Mutant competitor ARE** |
| --- | --- | --- | --- | --- |
| Protein Extract  (5 µg/µL) | 3 µL | --- | 3 µL | 3 µL |
| 10x Binding Buffer | 2 µL | 2 µL | 2 µL | 2 µL |
| 25 mM DTT/  2,5 % Tween 20 | 2 µL | 2 µL | 2 µL | 2 µL |
| Poly (dI-dC)  1 µg/µL | 1 µL | 1 µL | 1 µL | 1 µL |
| 1 M KCl | 1 µL | 1 µL | 1 µL | 1 µL |
| competitor oligo | --- | --- | 1 µL | --- |
| mutant competitor oligo | --- | --- | --- | 1 µL |
| IRDye 700 labeled oligo | 1 µL | 1 µL | 1 µL | 1 µL |
| Ultra-Pure water | up to 20 µL | up to 20 µL | up to 20 µL | up to 20 µL |
